# The minimal work cost of information processing

**DOI:** 10.1038/ncomms8669

**Published:** 2015-07-07

**Authors:** Philippe Faist, Frédéric Dupuis, Jonathan Oppenheim, Renato Renner

**Affiliations:** 1 Institute for Theoretical Physics, ETH Zurich, Wolfgang-Pauli-Str. 27, Zurich 8093, Switzerland; 2 Department of Computer Science, Aarhus University, IT-Parken, Aabogade 34, Denmark 8200; 3 Faculty of Informatics, Masaryk University, Botanická 68a, Brno 612 00, Czech Republic; 4 Department for Physics and Astronomy, University College of London, Gower Street, WC1E 6BT UK

## Abstract

Irreversible information processing cannot be carried out without some inevitable thermodynamical work cost. This fundamental restriction, known as Landauer's principle, is increasingly relevant today, as the energy dissipation of computing devices impedes the development of their performance. Here we determine the minimal work required to carry out any logical process, for instance a computation. It is given by the entropy of the discarded information conditional to the output of the computation. Our formula takes precisely into account the statistically fluctuating work requirement of the logical process. It enables the explicit calculation of practical scenarios, such as computational circuits or quantum measurements. On the conceptual level, our result gives a precise and operational connection between thermodynamic and information entropy, and explains the emergence of the entropy state function in macroscopic thermodynamics.

## Abstract (web summary)

Irreversible computation cannot be performed without a work cost, and energy dissipation imposes limitations on devices' performances. Here the authors show that the minimal work requirement of logical operations is given by the amount of discarded information, measured by entropy.

## Graphical abstract

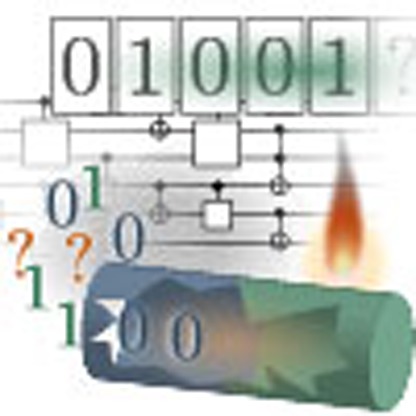

Thermodynamics in essence is an information theory—its purpose is to make statements about systems for which we only have certain partial information, such as a gas of many particles for which only macroscopic quantities like temperature, volume and pressure are accessible. Following this point of view, Jaynes showed that the entropy function derived in statistical mechanics corresponds to the information-theoretic entropy of the gas associated with a macroscopic observer who is maximally ignorant of the microscopic degrees of freedom^1^, resorting to Shannon's mathematical theory of information^2^ developed in the context of telecommunications.

When the observers have access to knowledge about microscopic quantities, such as positions and velocities of particles in a gas, the second law of thermodynamics seems to break down, as was illustrated by Maxwell's demon. To address this problem, Szilard^3^ studied a one-particle gas that can be located on either side of a box, left (‘L') or right (‘R'), and noted that by isothermally compressing the gas or letting the gas expand, one can trade this one bit of information for *kT* ln 2 work, as depicted in Fig. 1a (in the presence of a heat bath at temperature *T*, and where *k* is Boltzmann's constant). Landauer and Bennett later realized that the information content of data stored in a memory register, independently of the nature of its physical representation, counts as thermodynamic entropy when considering thermodynamical operations on that register^4^,^5^,^6^,^7^,^8^,^9^,^10^,^11^,^12^,^13^. For example, given a bit in an unknown state, any operation that resets it to zero must dissipate at least *kT* ln 2 heat, and thus the corresponding amount of work must be supplied (this is known as Landauer's principle). This fact salvages the second law of thermodynamics and resolves the paradox of Maxwell's demon.

More recently with the advent of quantum information, efforts were made to understand the laws of quantum thermodynamics from an information-theoretic viewpoint^14^,^15^,^16^,^17^,^18^, while the increasing technological ability to control and manipulate nanoscale systems^19^,^20^ has prompted the study of particular operational models and frameworks, leading to characterization of the work cost of various information-theoretic tasks such as erasure and work extraction^21^,^22^,^23^,^24^,^25^,^26^,^27^,^28^,^29^,^30^,^31^,^32^. For a more specific review of existing results, we refer to (Supplementary Note 1).

The aim of this work is to study thermodynamics in such generalized scenarios, where one may have knowledge about microscopic degrees of freedom, by resorting to modern tools of information theory^33^,^34^. We provide a fundamental lower bound to the work cost of a physical implementation of a logical process, discuss several examples and illustrate how traditional thermodynamics emerges from our micrsocopic result in the limit of macroscopic systems.

## Results

### The Framework

We determine a general expression for the minimal amount of work needed to carry out any given logical process 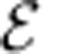. This can be for example an AND gate or any quantum or classical computation; most generally 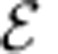 is defined as any completely positive, trace-preserving map from quantum states on an input Hilbert space 
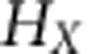
 to quantum states on an output Hilbert space 
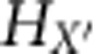
. We assume these spaces to be of finite dimension for simplicity; note that such a space can be a subspace of an infinite-dimensional Hilbert space in which the relevant computation or logical process takes place. The terminology ‘logical process' is meant to emphasize that the mathematical object 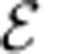 only specifies for each input state the corresponding output state and does not prescribe its physical realization, which would consist of a full description of a physical system including the parts of its environment that are relevant to determine its time evolution. Note that in performing a logical process one does not merely transform one quantum state into another; rather, the output must be related to the input in a precisely specified way. In the case where the input is a classical value, this means that the output depends on the particular input value received, and not only on the distribution of inputs. This might be checked in practice, for example, if one keeps a copy of the input as a reference system and observes the correlations between the output and the reference system.

There are, generally, many ways of actually realizing a logical process with an actual physical device. The device and its interactions with the environment (for example, a heat bath) may for example be described by a Hamiltonian or a Liouvillian. For our purposes, it is sufficient to specify the set of operations which the device is allowed to perform as well as the associated work cost. We then optimize the work expenditure over precisely those strategies, which realize the given logical process 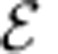. Observe that the more permissive our framework is, the more robust our bound will be. In our model, we shall be allowed to implement at no work cost any trace-preserving completely positive map that is unital, that is, which preserves the identity operator. Note that if we were to allow any logical process that is not unital to be performed for free, one could flagrantly violate the second law of thermodynamics on a macroscopic scale: in this sense, unital maps are the most permissive logical operation that we can allow for free. The model must also include a description of a ‘battery' that provides the energy required to drive the process. For this we resort to Bennett's idea of an ‘information fuel tape'^5^,^11^: such a battery consists of a large number of qubits with a degenerate Hamiltonian. Initially, a certain number *λ*_1_ of these qubits are in the maximally mixed state and the rest are pure. We may freely implement any joint unital map on the system and battery. At the end of the operation, the state of the battery consists of a possibly different number *λ*_2_ of qubits in a maximally mixed state, while the rest should be pure (The requirement that these *λ*_2_ qubits be maximally mixed is not a restriction, see Methods section.). We then count the amount of work consumed as *W*=*kT* ln 2·(*λ*_2_−*λ*_1_), which is the amount of work required to restore the battery system into its initial state. Indeed, a vast amount of literature has well underscored the correspondence between possessing a pure degenerate qubit, or storing *kT* ln 2 work, and vice versa^3^,^5^,^11^,^12^. The quantity *W* may be negative, indicating that work can be extracted from the battery when restoring it to its initial state. In addition, we assume that the input to the logical process 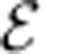 is encoded in a system whose initial Hamiltonian is degenerate. The same is assumed about the output system at the end of the computation. Note that this does not exclude making use of systems with nontrivial Hamiltonians during the implementation of the process. Also, this requirement is in practice not a limitation, as many other frameworks may be mapped to this setting^26^,^28^,^29^; indeed the assumption should rather be regarded as a technicality to ensure a clean way of accounting for work.

To obtain physically relevant results, we also have to exclude overwhelmingly unlikely events from our considerations. This is actually quite common in thermodynamics and is usually done implicitly. For example, consider a stone lying on the ground. There is a very small chance that by thermal fluctuation the stone spontaneously jumps in the air. However, this event is so disproportionately unlikely that in a physical theory we may safely choose to ignore this possibility. Within our framework, we do this more explicitly. That is, we consider a parameter that specifies the total probability of all events we want to exclude. In the quantum regime, where events are generally not well-defined, this idea is captured by 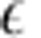-approximations: the stone has a very small amplitude of being found in the air, but its state is 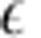-close to a state completely located on the ground. Analogously, we study the work requirement of logical processes that are 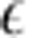-approximations of the desired logical process. This is a standard procedure in information theory^33^,^34^, and is justified by the fact that an 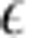-approximation cannot be distinguished from the original logical process with probability greater than 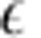.

### The main result

**Figure 1:**
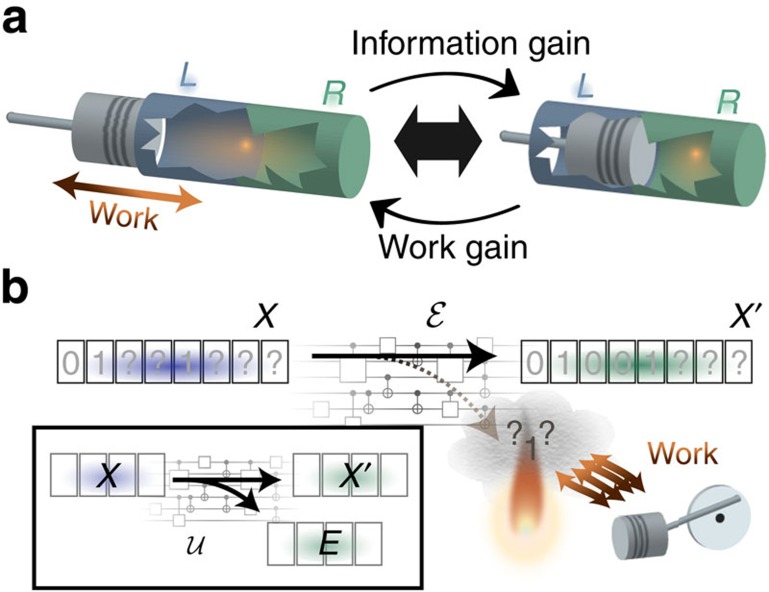
Work and information are related by physical processes. (**a**). A gas formed by a single particle can either be on the left (‘L') or the right (‘R') side of the cylinder (known as a Szilard box^3^). This one bit of information can be reversibly traded for *kT* ln 2 work by isothermally compressing the gas with a piston or letting the gas isothermally expand. This illustrates that discarding 1 bit of entropy (or uncertainty) requires *kT* ln 2 work. (**b**) An implementation of the logical process 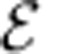 mapping a system *X* to an output *X*′ interacts with the thermal bath may discard information and in general costs work. The logical process 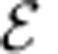 may be written as part of a global unitary 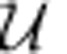 on an additional hypothetical system *E*, which represents the discarded information (inset). Our main result states that the minimum work required in a physical implementation of 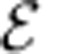 is the amount of discarded information, which the implementation has to dump into the environment.

To formulate our main claim, we represent the logical process 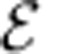 by its Stinespring dilation^35^. This is an isometry 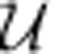 (which can be seen as part of a unitary) that maps *X* onto *X*′ as well as an extra system *E* such that the original map 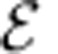 is retrieved by ignoring *E* (see Fig. 1b). Our main result asserts that 
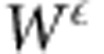
, the work one needs to supply to execute the operation up to an 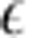-approximation, is lower bounded by


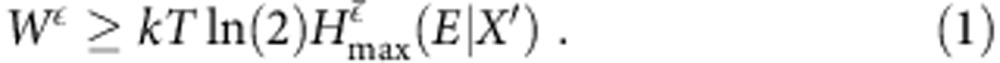


The right hand side is the smooth max-entropy of *E* conditioned on *X*′ and may be interpreted as a measure for the irreversibility of the logical process. More precisely, the smooth max-entropy is an information-theoretic measure defined in the Methods section, and quantifies the uncertainty one has about *E* when given access to *X*′. The parameters 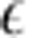 and 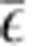 are related by 
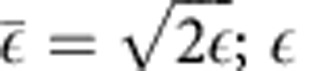
 may be chosen arbitrarily. We stress that the system *E* is an abstract mathematical concept used to represent the logical map 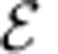, and can be interpreted as the information discarded by the mapping. In particular, our bound is independent of the choice of this representation.

The form of the bound (1) naturally expresses our intuition that the amount of work that needs to be provided corresponds to the amount of information that is logically discarded, and which therefore has to be dumped into the environment. This consideration is done from the viewpoint of the observer who has completed the computation, and thus has access to *X*′, explaining the occurrence of the conditional entropy. Also, if *E* is classical, the max-entropy has the operational interpretation of being the amount of memory space needed to compress the information contained in *E* when possessing knowledge of *X*′ (ref. 36) (In the fully quantum case, it corresponds to quantum state merging^37^.).

The proof of our main result proceeds by first considering the special case in which 
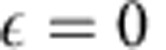
. The bound one then obtains is


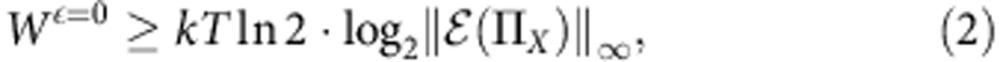


where Π_*X*_ is the projector onto the support of the input state. This expression proves particularly useful for calculating some simple practical examples.

The proof of this special case, and its generalization to the regime where 
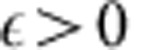
, is presented in the Methods section. An alternative proof, using techniques from majorization, is given in (Supplementary Note 5).

### Classical mappings and dependence on the logical process

**Figure 2:**
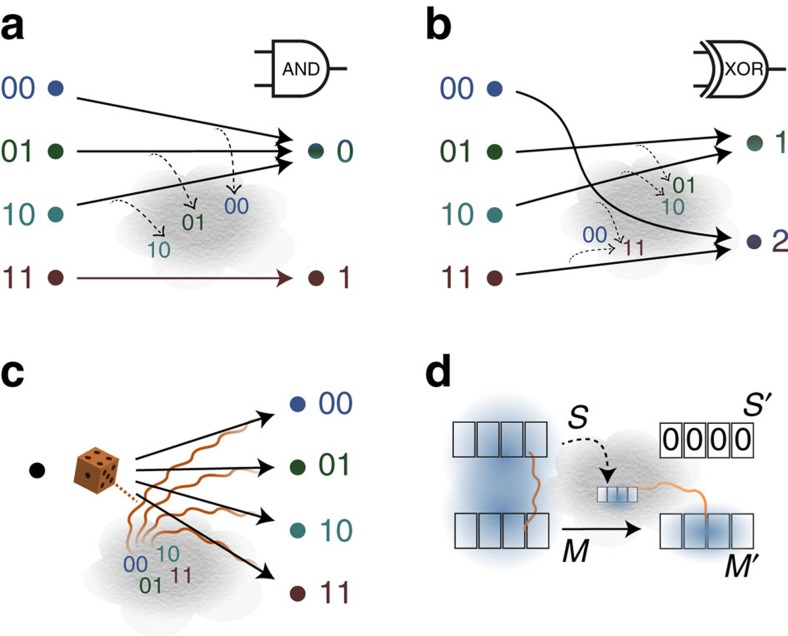
Examples of logical processes. (**a**) The AND gate is one of the building blocks of computers. Our result implies that any successful implementation of this logically irreversible gate requires at least work log_2_ 3·*kT* ln 2≈1.6 *kT* ln 2 due to the entropy of the discarded information (dotted arrows). (**b**) The XOR gate only requires *kT* ln 2 work, as it discards less entropy per output event than the AND gate. (**c**) Work can be extracted if randomness is being produced: the discarded information is entangled with the output (orange wavy lines), and the conditional entropy on the right hand side of (1) is negative. (**d**) The erasure of a quantum system *S* with access to a quantum memory *M* must transfer the content of *S* into the system *E* containing the discarded information, while preparing *S*′ in a pure state and mapping *M* to *M*′ identically. The corresponding minimal work cost is 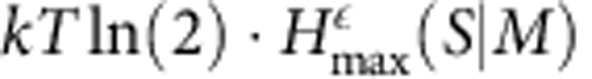; this can be achieved using the procedure of del Rio *et al*.^25^ If the system is entangled with the memory, this quantity is negative and work may be extracted.

Our result, which is applicable to arbitrary quantum processes, applies to all classical computations as a special case. Classically, logical processes correspond to stochastic maps, of which deterministic functions are a special case. As a simple example, consider the AND gate. This is one of the elementary operations computing devices can perform, from which more complex circuits can be designed. The gate takes two bits as input, and outputs a single bit that is set to 1 exactly when both input bits are 1, as illustrated in Fig. 2a.

The logical process is manifestly irreversible, as the output alone does not allow to infer the input uniquely. If one of the inputs is zero, then the logical process effectively has to reset a three-level system to zero, forgetting which of the three possible inputs 00, 01 or 10 was given; this information can be viewed as being discarded, and hence dumped into the environment. We can confirm this intuition with our main result, using the fact that a general classical mapping is given by the specification of the conditional probability *p*(*x*′|*x*) of observing *x*′ at the output if the input was *x*. Embedding the classical probability distributions into the diagonals of quantum states, the infinity norm in expression (2) becomes simply


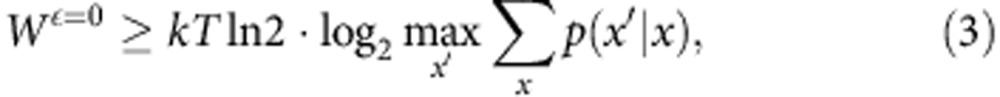


where the sum ranges only over those *x* that have a non-zero probability of occurring. In the case of deterministic mappings *p*(*x*′|*x*)∈{0,1}, this corresponds to the maximum number of input states that map to a same output state. For the AND gate, provided all four states 00, 01, 10 and 11 have non-negligible probability of occurring, there are three input states mapping to the same output state, so (3) gives us simply 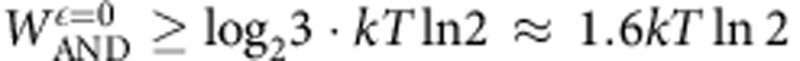. Also, in simple examples as considered here, the expression (3) is stable to considering an 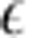-approximation (Supplementary Note 4); this quantity is thus physically justified.

Crucially, our result reveals that the minimal work requirement in general depends on the specific logical process, and not only on the input and output states. This contrasts with traditional thermodynamics for large systems, where the minimal work requirement of a state transformation can always be written as a difference of a thermodynamical potential, such as the free energy. For example, the minimal work cost of performing specifically an AND gate may differ from that of another logical process mapping an input distribution (*p*_00_, *p*_01_, *p*_10_, *p*_11_) (with ∑_*i*_ *p*_*i*_=1) to the distribution (*p*′_0_, *p*′_1_)=(*p*_00_+*p*_01_+*p*_10_, *p*_11_) (Recall that the classical counterpart of a quantum state is a probability distribution.). To see this, consider the XOR gate, which outputs a 1 exactly when both inputs are different (see Fig. 2b). The minimal work cost requirement of this gate, as given by (3), is now only *kT* ln 2, as in the worst case, only a single bit of information is erased (again supposing that all four input states have non-negligible probability of occurring). Now, suppose that, for some reason, the input distribution is such that *p*_01_+*p*_10_=*p*_11_, that is, the input 11 occurs with the same probability as of either 01 or 10 appearing. Then, the XOR gate reproduces the exact same output distribution as the AND gate: in both cases, we have *p*′_0_=*p*_00_+*p*_10_+*p*_01_=*p*_00_+*p*_11_ and *p*′_1_=*p*_11_=*p*_01_+*p*_10_. In other words, both logical processes have the same input and output state, yet the XOR gate only requires work *kT* ln 2 compared with the AND gate, which requires 1.6*kT* ln 2. Furthermore, we point out that this difference, which appears small in this case, may be arbitrarily large in certain scenarios (Supplementary Note 4).

On the one hand, we are by definition interested in the work cost of a given logical process, so one might have expected that this work cost should not only depend on the input and output states. On the other hand, it might seem contradictory that the full logical process matters even though we have fixed an input state *σ*_*X*_. However, this makes sense if we consider preparing the input state as part of a pure state on the input system and a reference system. In this case, the logical process that is implemented influences the (in principle detectable) correlations between the output and the reference system, even if the reduced state on the input is the fixed state *σ*_*X*_.

We emphasize that the phenomenon observed here is fundamentally different from the notion of thermodynamic irreversibility. Here we always consider the optimal procedure for implementing the logical process, whereas a thermodynamically irreversible process is in fact an ‘inefficient' physical process that could be replaced by a more efficient, reversible one. In our framework, the thermodynamically irreversibile processes are those physical implementations that do not achieve the bound (1). A longer discussion with examples is provided in (Supplementary Note 2).

### Work extraction

While erasure requires work, it is well known that in a wide range of frameworks one can in general extract work with the reverse logical process, which corresponds to taking a register of bits that are all in the zero state and making them maximally mixed^3^,^5^. Our result intrinsically reproduces this fact: the Stinespring dilation 
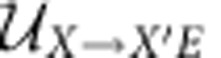
 of a logical process that generates randomness in fact creates entanglement between the output *X*′ and *E* (see Fig. 2c). The conditional entropy 
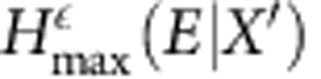
 then becomes negative, such that the bound (1) allows work to be extracted. We remark that, even if the logical process 
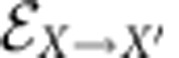
 is classical, the relevant state for the entropic term in (1) is entangled, and thus all but classical; this is due to the construction of *E* as a purifying system for the logical process.

### Erasure with a quantum memory and tightness of our bound

Recently, del Rio *et al*.^25^ have constructed an explicit procedure capable of resetting a quantum system *S* to a pure state using an erasure mechanism assisted by a quantum memory *M*, and doing so at a work cost of approximately


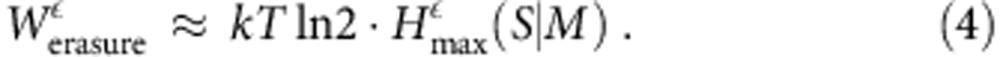


The approximation holds up to terms of the order of the logarithm of 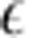 and are negligible in typical scenarios (Supplementary Note 4).

Our main result implies that their procedure is nearly optimal (Fig. 2d). Indeed, consider the total system 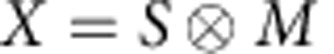, in the initial state *σ*_SM_, with the logical process 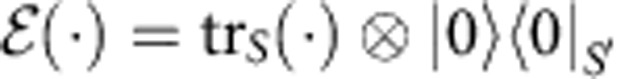, denoting symbolically with a prime the output system *S*′ (The state on *M* remains unchanged.). One then straightforwardly sees that the resulting joint state on *E* and the output 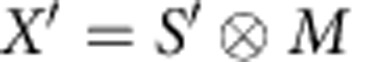 is obtained from the initial state on *S* and *M* by isometrically ‘transferring' the *S* part to *E* and replacing it by a fixed pure state. The entropy term in our bound (1) then becomes 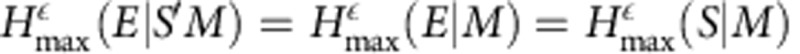, the latter entropy being evaluated on the input state. This matches the term in (4).

Conversely, this optimal erasure procedure can be used to show that for any arbitrary logical process, the minimal amount of work our result associates to it can be in principle achieved to good approximation. Given a logical process 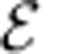 and an input state *σ*_*X*_, calculate its Stinespring dilation 
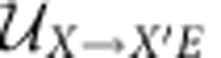
 as explained above, and consider an ancillary system *A*_*E*_ of the same dimension as *E*. This ancilla system is initialized in a pure state 
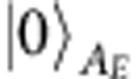
. One can then carry out a unitary 
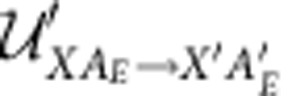
 on *X* and *A*_*E*_, chosen such that


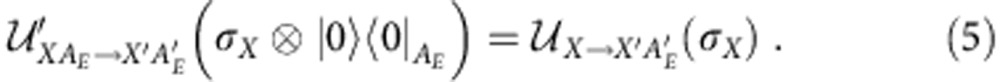


In effect, *A*′_*E*_ impersonates the abstract system *E* while we perform a unitary corresponding to the Stinespring dilation of 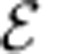 (see inset of Fig. 1b). This unitary operation can be implemented at no work cost because it is reversible. The aforementioned optimal erasure procedure can then be used to restore the ancilla *A*′_*E*_ to its original pure state, using the output system *X*′ as the quantum memory, at a work cost of approximately 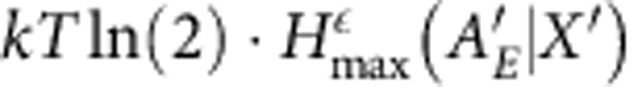. As *A*′_*E*_ corresponds to *E*, this matches our bound (1) and therefore proves its tightness.

### The work requirement of a quantum measurement

**Figure 3:**
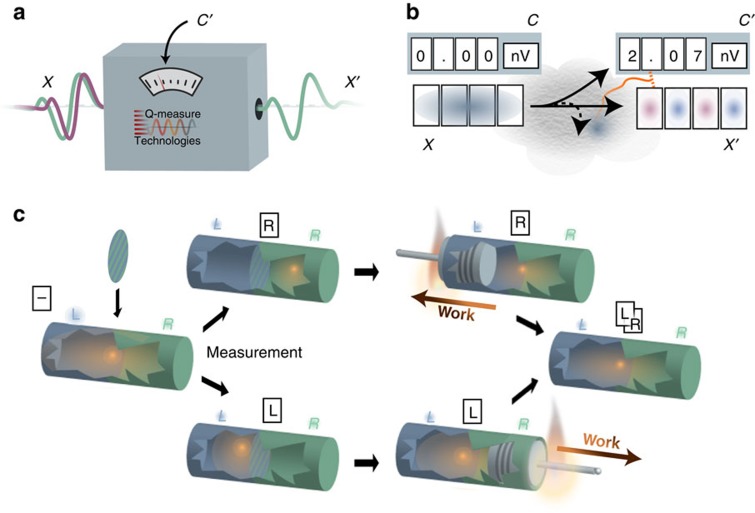
The work cost of quantum measurements. (**a**) A quantum measurement may be thought of as a device which produces a post-measurement state *X*′ and a classical reading *C*′ from an input state *X*. (**b**) The corresponding logical process maps the input system *X* and classical register *C* to a classical outcome on the output register *C*′ and a post-measurement state on *X*′. The initial register *C* is prepared in a pure state. Our main result implies that the measurement costs no work in principle. (**c**) Maxwell's demon with a Szilard box, as proposed by Bennett^5^. A measurement detects on which side of the inserted separator the particle is, and extracts work with a piston in either case. The cylinder is left in its original state, apparently creating a perpetuum mobile with net work gain. However, the measurement outcome (represented by ‘L' or ‘R') had to be stored in a memory register, which was initially in some pure state (represented by ‘—') and the work cost of resetting it to a pure state again compensates the work gain. The register could have been reset using the post-measurement state at no work cost, but the latter was consumed during work extraction.

The problem of determining the amount of work needed to carry out a quantum measurement has been the subject of much literature^38^,^39^,^40^, especially in the context of Maxwell's demon^5^,^6^,^12^,^41^. A quantum measurement is a logical process (depicted in Fig. 3a) acting on a system *X* to be measured and a classical register *C* initially set to a pure state, and outputting systems *C*′ and *X*′, with *C*′ containing the measurement result and *X*′ the quantum post-measurement state. We will consider a projective measurement for simplicity, treating the more general case in (Supplementary Note 4). The logical process corresponding to the measurement described by a complete set of projectors {*P*_*i*_}_*i*_ takes the form


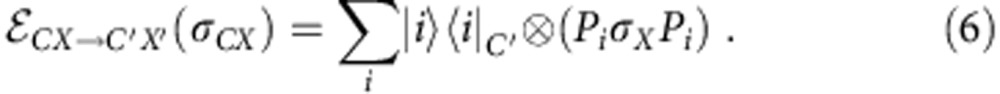


Our bound (2) for this map is at most zero (since 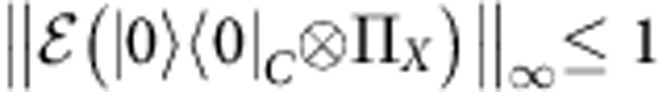), implying that the measurement can be carried out in principle at no work cost, as was already stated by Bennett^5^. Note that a work cost is required if the classical register *C* was not initially pure^40^.

A related question is the work cost of erasing the information contained in the register *C*′ after the measurement. Doing so would allow us to construct a cycle. The cost of this erasure can be reduced using the post-measurement state as a quantum memory, by employing the procedure presented above, to 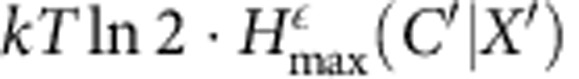. But because *C*′ and *X*′ may only be classically correlated, no work may be extracted in this way^25^. In some cases this work cost may be zero, for example for projective measurements on a maximally mixed state (Supplementary Note 4). This might seem to save Maxwell's demon from Bennett's information-theoretic exorcism, which argues that the demon must pay work to reset its memory^5^ (see Fig. 3c). However, the key point is to notice that the demon cannot use the post-measurement state to both extract work and to reset its internal memory register.

## Discussion

Our main result exposes various features of thermodynamics in the microscopic regime that are not present in the standard setting of large systems. In particular, as argued above, the minimum work cost of a logical process cannot be given in terms of a state function, such as the entropy or the free energy in thermodynamics.

Traditional thermodynamics is concerned with macroscopic systems, and we may retrieve this limit by considering logical processes that consist of many individual operations. Under appropriate independence assumptions and using typicality arguments^42^, one can show that the average minimal work cost per process as determined by (1) simply takes the form *kT* ln (2)·[*H*(*X*)−*H*(*X*′)], where *H*(*X*)=−tr(*ρ*_*X*_ log_2_ *ρ*_*X*_) is the usual von Neumann entropy (see Methods section): the minimal work requirement is now given by a function of state *H*(*X*), and no longer depends on the logical process that maps *X* to *X*′ (see Methods).

**Figure 4:**
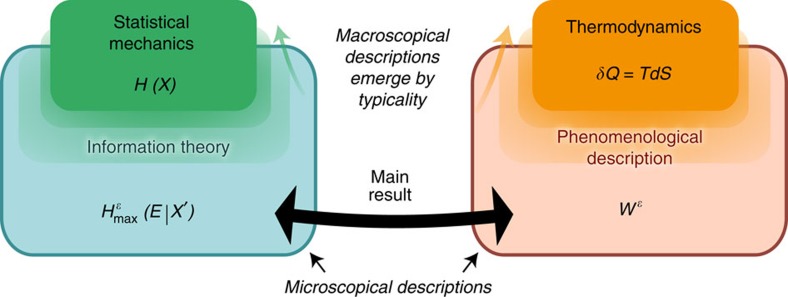
Relation between information-theoretic and thermodynamic quantities. Our result relates two quantities that depend on the microscopic details of the system: the information-theoretic entropy 
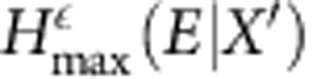
 that quantifies the amount of information discarded by the logical process, and the amount of work 
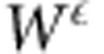
 needed to carry out a logical process on the microscopic level. Standard thermodynamics is obtained in the limit of macroscopic systems. In this limit, it follows from typicality arguments that the entropic measure 
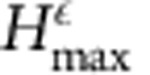
 converges to the von Neumann entropy *H*(*X*), which may thus be seen as an emergent quantity. Furthermore, in this regime, the minimum amount of work 
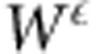
 used by a process corresponds to the heat *Q* that is reversibly transferred to the environment, which in turn is related to the thermodynamic entropy, *S*, as defined by Clausius. Our result thus permits the identification of the information-theoretic entropy *H*(*X*) for a macroscopic observer, that is, the entropy considered in statistical mechanics, with the thermodynamic entropy *S*.

Our result thus provides the following fresh view on the macroscopic regime. Thermodynamics can be seen as a general framework, in which the second law postulates the existence of a state function, the thermodynamic entropy, which relates to the heat flow in processes. Many standard results of thermodynamics follow from that starting point. It is now the role of a microscopic theory to construct a state function with this property, based on the microscopic dynamics of the particular system. In textbook statistical mechanics, this construction is given for several physical setups, such as gases or lattices; one usually considers, for example, the configuration entropy, or an appropriately normalized Shannon or von Neumann entropy of the density of the statistical ensemble. Our result generalizes this construction and clarifies when it is justified: the state function, in general, appears whenever the inherent fluctuations due to the microscopic stochastic nature of the process vanish by typicality. The existence of an entropy state function is therefore not a property of the microscopic system; it is rather an emergent quantity that appears whenever the full system is typical, such as in the limit of macroscopic processes (Fig. 4).

Finally, one should note that the system in consideration need not be large for the typicality arguments to apply. For example, if one considers the work requirement of performing many independent repetitions of a single given logical process (seen as one big joint process), then the work requirement 
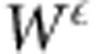
 per repetition converges to the average work requirement as calculated via statistical mechanics, even if the individual system is small: in this case, the entropy function emerges. This further justifies the usage of the von Neumann entropy in statistical mechanics even for small systems. Conversely, a large system does not necessarily display typicality; such is the case for systems out of thermodynamic equilibrium. An explicit example is provided in (Supplementary Note 4).

In summary, our main result quantifies the minimal required work to perform a logical process on the microscopic level. On the conceptual level, our result shows how, for macroscopic systems, the information-theoretic von Neumann entropy emerges as a state function and can thus be strictly identified with the thermodynamic entropy.

## Methods

### Mathematical formulation and proof of the main result

The task is to implement the logical process 
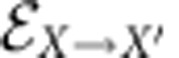
. Recall the framework allows for the implementation of any unital map, that is, 
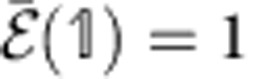
, to be performed on the systems at hand. We first adapt a well-known classical result about doubly stochastic and doubly sub-stochastic matrices^43^ to relate unital quantum maps to so-called subunital maps, that is, maps 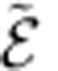 that satisfy 
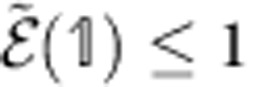
. Note also that the composition of two unital maps is unital, and similarly the composition of two subunital maps is subunital. We will need the following proposition, which we prove in (Supplementary Note 6) as Prop. 17.

*Proposition I (dilation of a subunital map)*. Let 
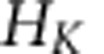
 and 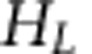 be finite dimensional Hilbert spaces, and let 
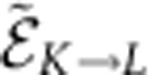
 be a completely positive, trace-nonincreasing, subunital map. Then there exists finite dimensional Hilbert spaces 
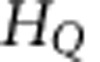
 and 
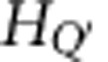
, and a completely positive, trace-preserving, unital map 
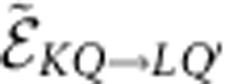
 such that


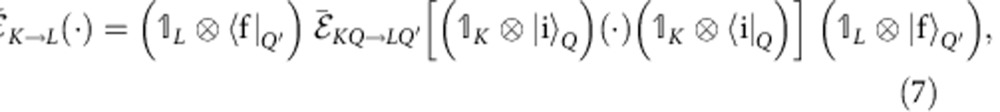


for some pure states |i〉_*Q*_, |f〉_*Q*′_. In addition, dim (
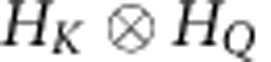
)= dim (
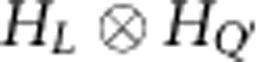
).

Let's now denote by *A* the ‘information battery' system, which is the physical system that tracks how much work we have used or extracted. The system *A* may be as large as we might wish (but finite) and starts in a state 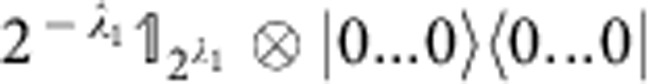 with some given number of mixed qubits *λ*_1_. The system *X* starts in a given state *σ*_*X*_, and we assume that the Hamiltonians of *X* and *A* vanish at the beginning and at the end of the physical process.

Our framework specifies that we are allowed to perform any sequence of joint unital operations on any subsystems of *X* and *A*. The final state on 
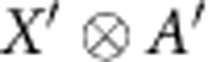
 should be a product state, with the state on *A*′ of the form 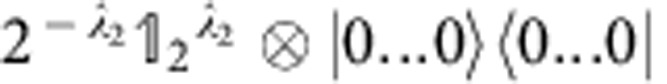. Note that the structure imposed on this state is not a restriction: if the final state on *A*′ is not of this form, an additional unital map can be applied on the support of the final state on *A*′ to replace the latter by a maximally mixed state on its support. However, this condition does assume that there is no way to extract work while transforming a state *ρ* to a maximally mixed state of the same rank, or, equivalently, that the worst-case erasure cost of a state *ρ* is *kT* ln 2 log_2_ rank *ρ*. This can usually be seen as a consequence of the choice of framework, and is in line with the findings of refs 28, 29. Alternatively, given a state *ρ*, let *m* be its rank, *p*_min_ its smallest non-zero eigenvalue and Π the projector on its support. The state *ρ* may be written as a statistical mixture of 
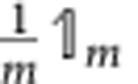
 with probability *m*·*p*_min_ and some state (*ρ*−*p*_min_Π)/(1−*m p*_min_) with probability 1−*m*·*p*_min_. In the event where the system is prepared in the maximally mixed state of rank *m*, the work requirement for erasure is deterministic because the state is uniform, and equals *kT* ln 2 log_2_ *m* (refs 3, 5, 11, 12); it follows that the work required for erasing *ρ* with certainty is at least *kT* ln 2 log_2_ rank *ρ*.

Observe that our framework is equivalent to allowing the agent to perform a single unital operation on the whole of *X* and *A*, leaving both systems in the state 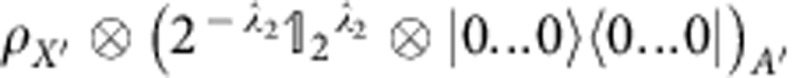: indeed the composition of unital maps is unital, and extending a unital map by an identity map still yields a unital map.

Even though we have presented our results while hinting that *X* and *X*′ represent the same system, and are thus of the same dimension, this need not be the case: our results are valid for arbitrary finite dimensions of *X* and *X*′. However, we will assume that one can bring in ancillas of arbitrary finite dimension in pure states and dispose of ancillas restored to a pure state for free. Henceforth, we will assume that such ancillas are counted as part of the pure systems composing the work storage systems *A* and *A*′ (The systems *A* and *A*′ hence need not be of same dimension.).

We must in addition require that the physical process implement the logical process 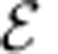. Let |*σ*〉_*XR*_ be a purification of *σ*_*X*_ on a system *R*. If one applies the physical process to *X* while leaving *R* untouched, then the state on 
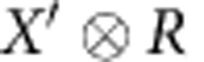
 that results from the physical process must be equal to the state *ρ*_*X*′*R*_ that would result by applying the mapping 
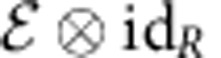
 on *σ*_*XR*_, that is, 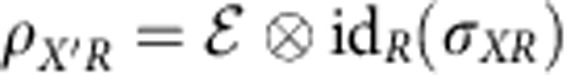. Observe that this constraint is equivalent to requiring the logical mapping corresponding to the physical process to be exactly 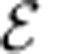 on the support of *σ*_*X*_, due to the Choi-Jamiołkowski isomorphism. So, even with a fixed given input state *σ*_*X*_, the full information about the mapping can be observed in the resulting state on 
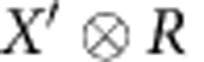
, by keeping a purification of *σ*_*X*_: in other words, the full information about the mapping and the input state is one-to-one encoded in the bipartite state *ρ*_*X*′*R*_.

Let's now state a formal version of our problem, in the case where we do not yet consider an 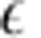-approximation. The task is to find the minimal *kT* ln2·(*λ*_2_−*λ*_1_), such that there exists a unital, trace-preserving, map 
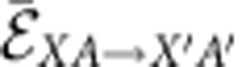
 satisfying


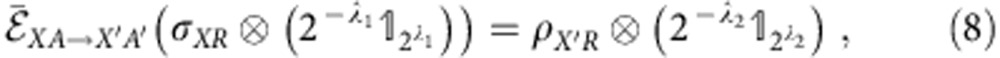


where 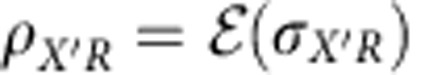 and where an identity mapping on *R* is implicitly understood (We henceforth omit the pure states on system *A*, that is, the factors ‘
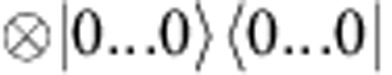
' above, for readability.).

At this point, note that whenever for given *λ*_1_, *λ*_2_, there is such a unital map, then there is also a subunital map achieving the same logical process and vice versa. Let's write this as a proposition:

*Proposition II*. Let *λ*_1_, *λ*_2_⩾0 and let 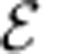 be given. Then are equivalent

(1) For a large enough *A*, and corresponding *A*′, there exists a trace-preserving unital map 
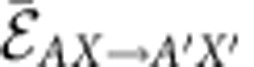
 such that


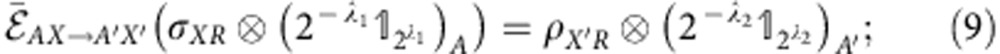


(2) For a large enough *B*, and large enough *B*′, there exists a trace-nonincreasing subunital map 
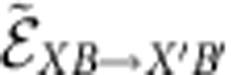
 such that


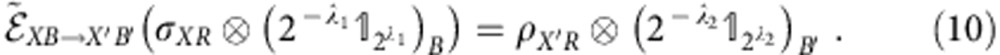


*Proof.* The forward direction is straightforward, as a unital map is in particular subunital. For the converse, we will dilate the given subunital map 
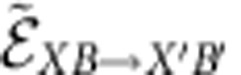
 to a unital map using Prop. 1, with 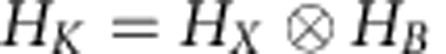 and 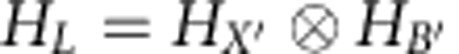: let 
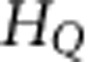
, 
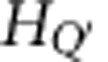
 and 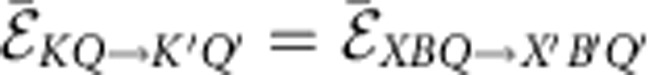 be given by the Proposition. Now define 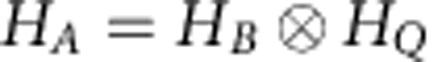 and 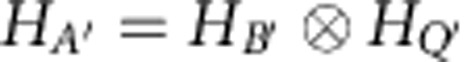. We would like to show that 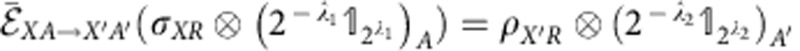, where we have defined 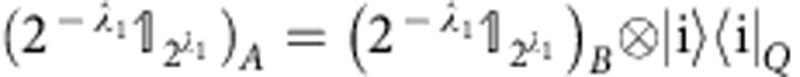 and 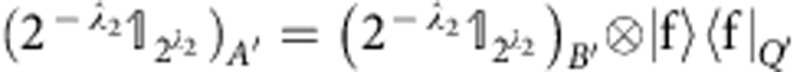 (as pure states, |i〉_*Q*_ and |f〉_*Q*′_ do not alter the amount of work stored in the work storage systems *A* and *A*′). Define also the shorthand 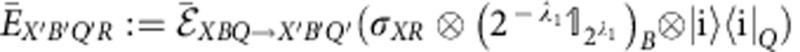. By construction, and using (7), we have


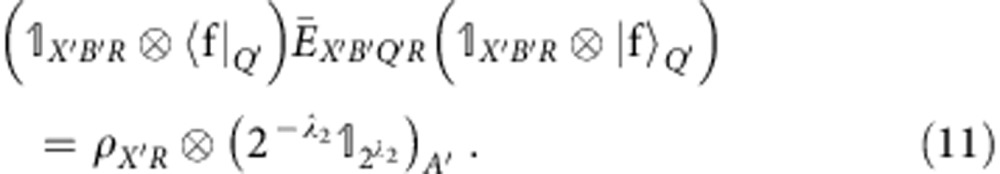


Since 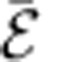 is trace-preserving, we have tr (
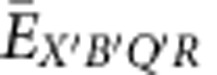
)=1 and


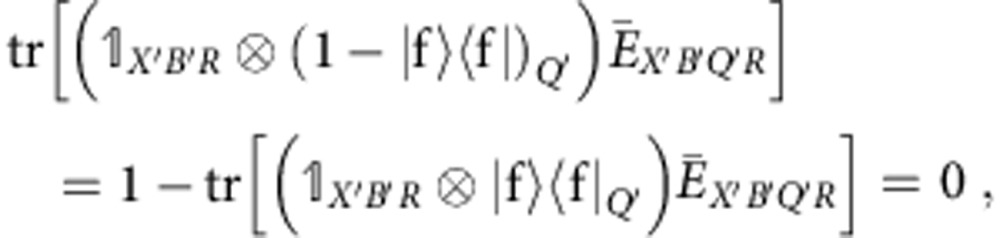


as the expression in (11) has unit trace. It follows that 
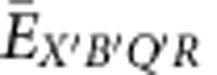
 lies in the support of 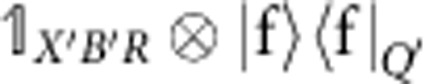, and from (11) we conclude as requested that


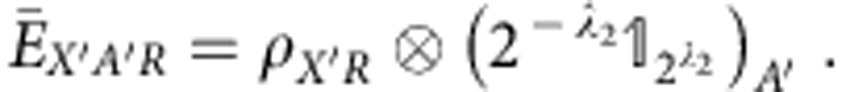


We can now characterize the allowed operations in our framework and their work costs with the following proposition.

*Proposition III*. Let *σ*_*X*_, 
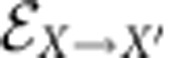
 be given. Choose system *B* big enough and let be given integers *λ*_1_, *λ*_2_⩾0. Then are equivalent:

(1) There exists a trace-nonincreasing subunital map 
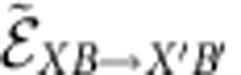
 such that


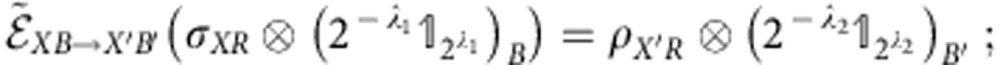


(2) There exists a trace-nonincreasing map 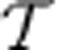, mapping linear operators on 
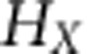
 to linear operators on 
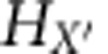
, such that 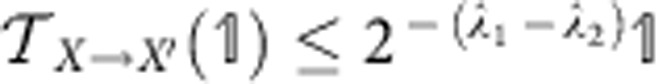, and 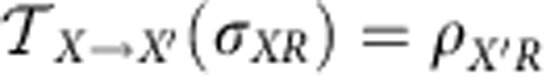;

(3) The map 
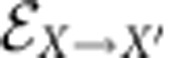
 satisfies 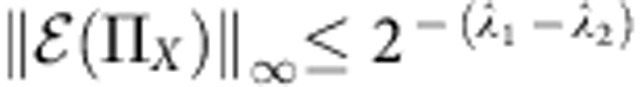, where Π_*X*_ is the projector onto the support of *σ*_*X*_.

*Proof.* (i)⇒(ii): Define 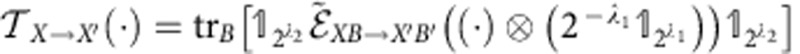. Then, 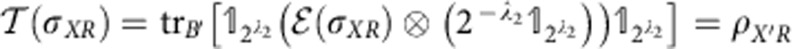. Also, 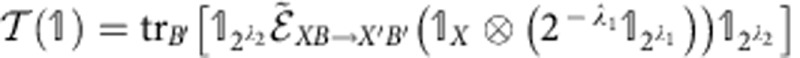 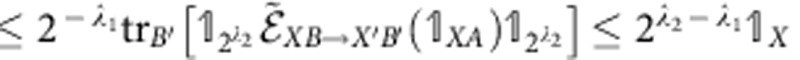, because 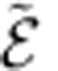 is subunital.

(ii)⇒(iii): We have 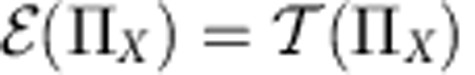 because the maps are equal on the support of *ρ*_*X*_ (alternatively, operate tr_*R*_[(·)*ρ*_*R*_^−1^] on both sides of 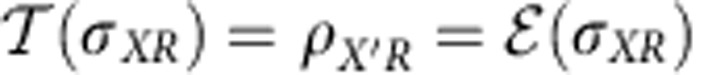 noting that *ρ*_*R*_=*σ*_*R*_); then because Π_*X*_≤_*X*_, we have 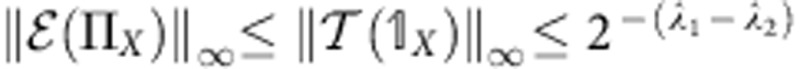.

(iii)⇒(i): Let 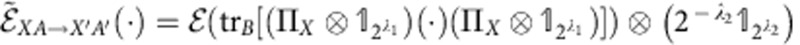. Observe that 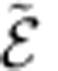 is subunital: 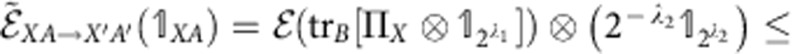 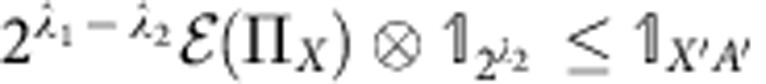. Also, 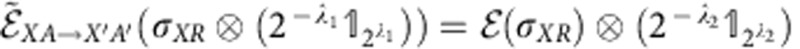, because the input to 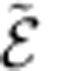 is inside the support of 
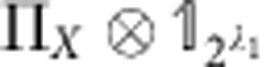
. Hence, 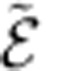 satisfies the conditions of (i).

With these propositions, we can calculate straightforwardly and explicitly the minimization in the formulation of the main problem. It now reduces to the simple question of minimizing *λ*_2_−*λ*_1_ subject to 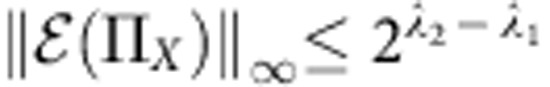; we have thus proven (2).

### Entropic form of the bound

Some basic facts about the smooth entropy framework are necessary to understand the rest of this section. For a more complete introduction on the smooth entropy framework, we refer to (Supplementary Note 3).

An equivalent definition of the Rényi-zero conditional entropy, also known as alternative max-entropy, for a bipartite state *ρ*_*AB*_, is given as


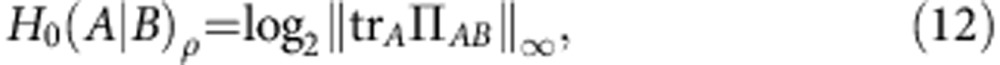


where Π_*AB*_ is the projector on the support of *ρ*_*AB*_. For consistency with the standard literature, we will express our final result in terms of the max-entropy, which is related to the Rényi-zero entropy up to factors logarithmic in 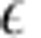 (ref. 34). The non-smooth conditional max-entropy can be defined as


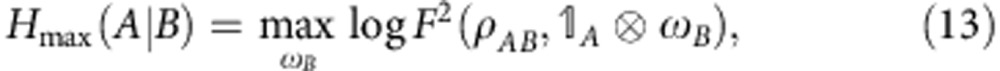


where 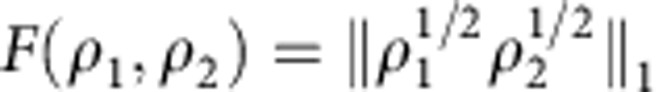 is the fidelity between two quantum states^35^, and where the optimization ranges over density operators on *B*. The smooth conditional max-entropy is defined by ‘smoothing' the max-entropy on states that are 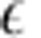-close to *ρ*_*AB*_ in fidelity distance:


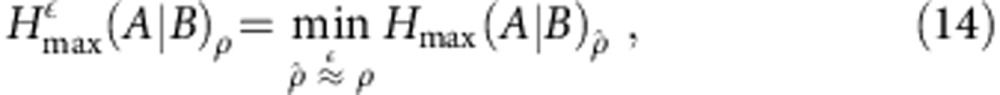


where the minimization ranges over all 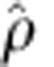 such that 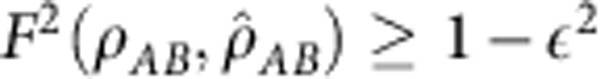.

Let's now return to our bound (2). Consider the Stinespring dilation of 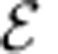, given by an isometry *V*_*X*→*X*′*E*_ including an additional system 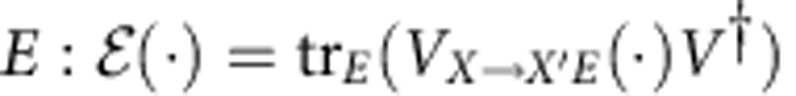. Defining the pure state *ρ*_*X*′*ER*_=*Vσ*_*XR*_*V*^†^ is obviously compatible with our previous definition of *ρ*_*X*′*R*_, as 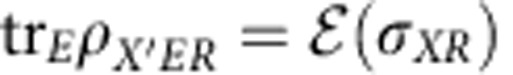. It follows that *V*Π_*X*_*V*^†^=Π_*X*′*E*_, where Π_*X*′*E*_ is the projector on the support of *ρ*_*X*′*E*_. Recalling (12), we have


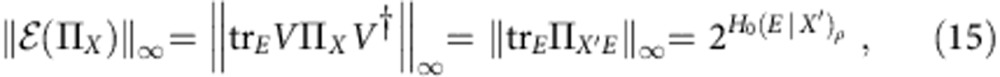


and our bound (2) takes the form


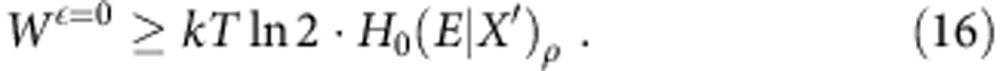


### Considering an 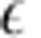 -approximation

A ‘smooth' version of the result is straightforward to obtain. In this case, we allow the actual process to not implement precisely 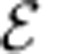, but only approximate it well. The best strategy to detect this inexactness is to prepare |*σ*〉_*XR*_ and send *σ*_*X*_ into the process, and then perform a measurement on *ρ*_*X*′*R*_. To ensure that the approximate process is not distinguishable from the ideal process with probability greater than 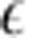, we require that the trace distance between the ideal output of the process *ρ*_*X*′*R*_ and the actual output 
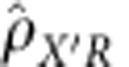
 must not exceed 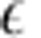. We can apply our main result to the approximate process that brings σ to 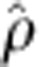, and lower bound the work cost of that process by


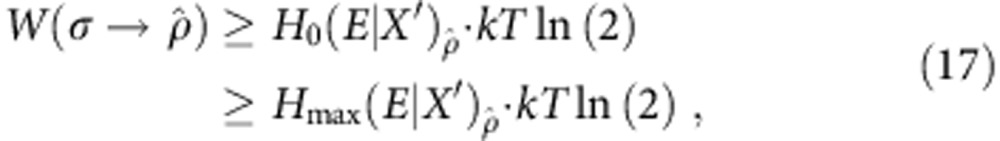


where the second inequality is shown in ref. 44 This relaxation of *H*_0_ to *H*_max_ is done for the sake of presentation and consistency with other results within the smooth entropy framework. When smoothing with a parameter 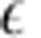, there is no significant difference with this relaxation: indeed, the two quantities are equivalent up to adjustment of the 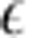 parameter and up to a logarithmic term in 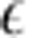 (Lemma 18 of ref. 44).

If we optimize (17) over all possible maps 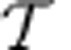 that output such 
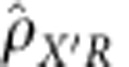
, we obtain a bound on the work requirement of the 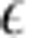-approximation,


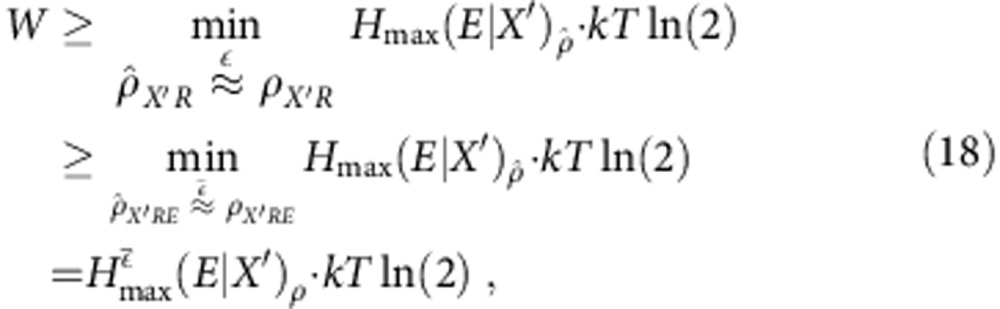


where the first optimization ranges over all 
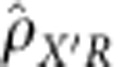
 such that the trace distance 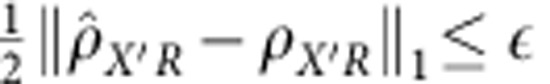, and where the second optimization ranges over all 
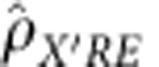
 such that 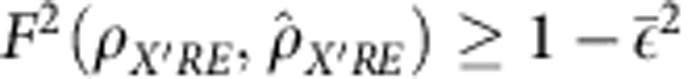, with 
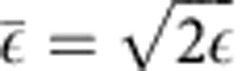
, where 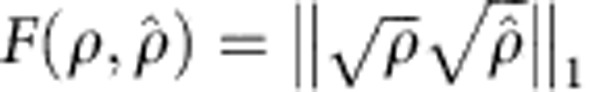 is the fidelity between the quantum states *ρ* and 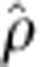.^35^

### Macroscopic limit: many independent repetitions

As we have seen in the introduction, considerable previous work has focused on the limit cases where many i.i.d. systems are provided. In such a case, the process 
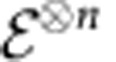
 is applied on *n* independent copies of the input 
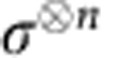
, and outputs 
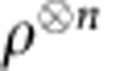
. A smoothing parameter 
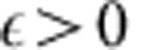
 is chosen freely. We may simply apply our (smoothed) main result to get an expression for our bound on the work cost,


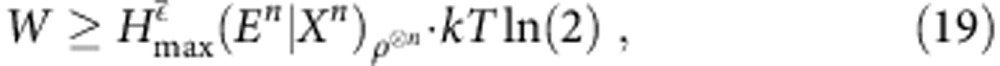


However, it is known that the smooth entropies converge to the von Neumann entropy in the i.i.d. limit^42^,


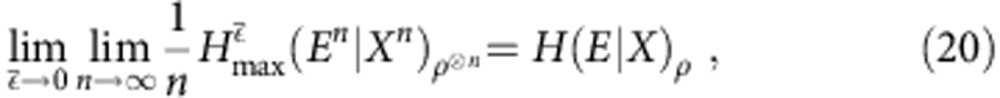


which allows us to simplify the expression of the work cost per particle, or per repetition of the process, to


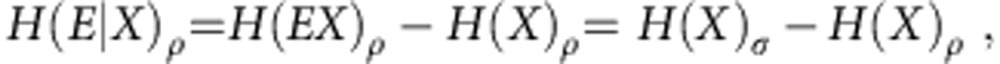


where the last equality holds because *ρ*_*EX*_ and *σ*_*X*_ have the same spectrum being both purifications of the same *ρ*_*R*_=*σ*_*R*_. We conclude that in the asymptotic i.i.d. case, the work cost is simply given by the difference of entropy between the initial and final state,


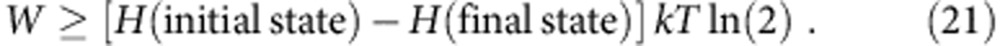


Here *W* is the average work cost per particle, or per repetition of the process. In the case for example of many independent particles undergoing a similar, independent process, the total work *W* required is obtained by considering the entropy of the full system of all particles in both terms in (21).

## Additional information

**How to cite this article**: Faist, P. *et al*. The minimal work cost of information processing. *Nat. Commun.* 6:7669 doi: 10.1038/ncomms8669 (2015).

## Supplementary Material

Supplementary Information Supplementary Figures 1-3, Supplementary Note 1-6 and Supplementary References
