# Supplementary material for: The minimal work cost of information processing
Source: Nat Commun. 2015 Jul 7;6:7669. doi: 10.1038/ncomms8669 (PMC4506503; doi:10.1038/ncomms8669)
Supplement: Supplementary Information — Supplementary Figures 1-3, Supplementary Note 1-6 and Supplementary References [file ncomms8669-s1.pdf]

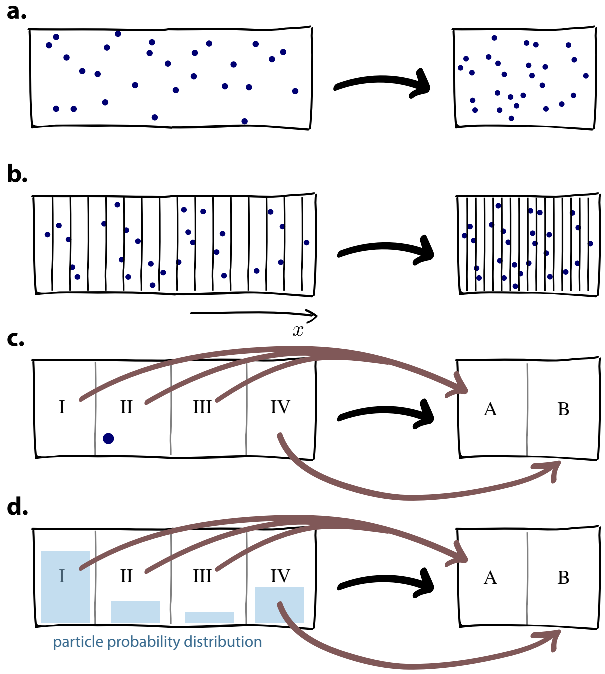

Supplementary Figure 1. *Thermodynamical* processes can be used to physically implement abstract *logical* processes. **a.** An isothermal compression implements the logical process corresponding to randomizing the position of the particles, within half the original volume. **b.** In this logical process, the X positions of the particles are mapped to half their value. This can be implemented by introducing many separators, resolving the position of the particles to a good enough precision, and then performing isothermal compression of each slice of gas. This procedure has same optimal work cost as the previous one, even though the *logical* processes are different. **c.** A single particle gas can be used to model an AND gate. The particle is supposed to be brought in output region A if it was originally in regions I, II or III, and to region B otherwise. The work cost fluctuates because of the probabilistic nature of the input state. **d.** If the probabilities that the particle initially resides within the different regions are not equal, the same amount of work as in the previous case is needed. However, if only the correct output state is to be reproduced, less work is required in some situations.

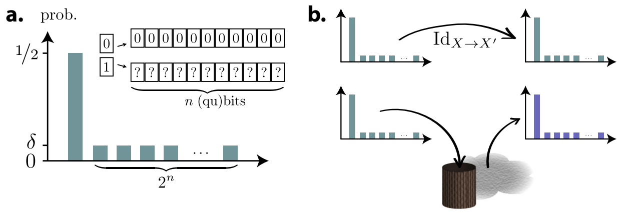

Supplementary Figure 2. Examples of large, non-typical distributions. **a.** The probability distribution (given by the spectrum of the state) of a classical system of one random qubit, along with n other qubits that are all 0 if the first qubit is 0, or uniformly random otherwise. **b.** Two different operations on this system may have the same input and output state, yet their work cost may differ arbitrarily. The first operation copies its input to its output (identity map), which costs no work. The second destroys the input and reproduces a fresh system at the output.

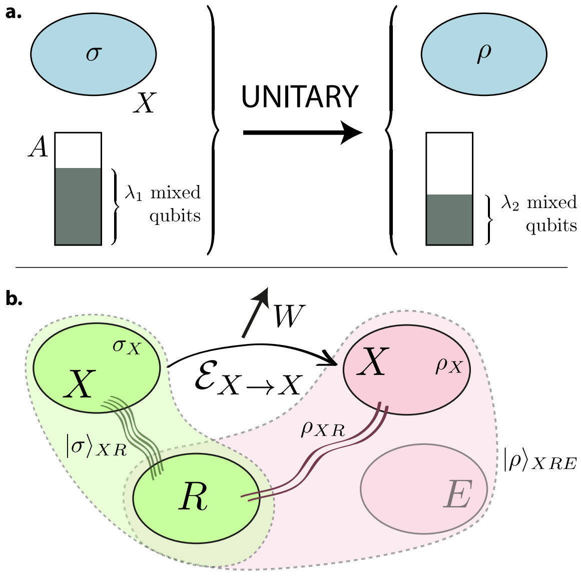

Supplementary Figure 3. **a.** Lambda-Majorization corresponds to absorbing a certain amount of randomness from an ancilla during a unitary operation. The system X starts in state σ, and the ancilla A in a state with λ_1 fully mixed qubits with the remaining qubits pure. The goal is to devise a global unitary that will bring the system X to the state ρ, while leaving the least possible number λ_2 of fully mixed qubits in A. The difference $\lambda = \lambda_1 - \lambda_2$, is the work extracted by the process; if the value is negative, it corresponds to a work cost. **b.** Our main result gives a fundamental lower bound on the work cost W of a process transforming a state σ_X (purified by a fictitious $|\sigma\rangle_{XR}$) into a new state $\rho_{X'R}$ obtained by applying a process $\mathcal{E}_{X\to X}$. We optimize the work cost of lambda-majorization operations that perform the process $\mathcal{E}$. The lower bound to the work cost is then given by the entropy of the information E that the process $\mathcal{E}$ has to discard (which purifies the state $\rho_{X'R}$), as measured by the Rényi-zero conditional entropy $H_0\left(E|X'\right)_\rho$.

SUPPLEMENTARY NOTE 1 – MOTIVATION. RELATION OF OUR RESULT TO PREVIOUS WORK.

The relation between thermodynamics and information, and in particular between the statistical Gibbs entropy and the information-theoretic entropy has been extensively studied from various perspectives. We give a short overview in this section; for a rather comprehensive discussion we suggest Ref. [1].

The fundamental question was raised by Maxwell in the 19th century, who imagined a perpetuum mobile on a gas divided into two chambers, whose net effect was the reduction of entropy of an isolated system: a small being could have knowledge of the microscopic degrees of freedom of the gas and operate a trap door in the splitting wall, which he could use to filter the cold molecules from the hot molecules. Szilard [2] realized that the crucial part of the problem was that the demon accessed microscopic degrees of freedom, which are not accessible normally in thermodynamics. He devised a thought experiment, the Szilard box (see main text), which illustrated the reversible conversion of $kT \ln 2$ work from or into well-defined accessible information. This suggested that the demon had to perform work to compensate for the entropy decrease of the gas. Scientists at the time were then led to believing that the measurement itself was a process that had to cost work, and some thought models were developed [3, 4]. It was Landauer in 1961 [5] who first associated work cost to the *logical irreversibility* of an operation, and who stated that the erasure of 1 bit of information had to cost $kT \ln 2$ work, and studied in particular the example of a particle in a double-V shaped potential [5, 6]. Bennett showed that computations can be made completely reversible and devised an explicit measurement apparatus which required no work. On the other hand, resetting the demon's memory back to its original state does cost work, effectively exorcising Maxwell's demon [7–11]. Landauer's principle has been criticized [12, 13], but became widely accepted as alternative proofs were proposed [14, 15] (cf. also [16]) and its conceptual importance clarified [9]. Various physical computational models were explored [17–20], while general considerations relating information and physics were discussed [21–24]. These efforts were lead in parallel to Jaynes showing the relevance of information theory for statistical mechanics and thermodynamics [25–28].

With the development of quantum information in the last decades, importance was given to generalizing Landauer's principle to the quantum regime [29–34], resulting in replacing the Gibbs, or Shannon, entropy with the quantum von Neumann entropy as relevant measure of information-theoretic entropy. These studies were motivated by the important technological advances making it possible to construct microscopic thermodevices [35–43]. Efforts were also undertaken to understand the laws of thermodynamics from an information-theoretic point of view [44–50].

While most information-theoretic approaches had studied averages over many independent repetitions of the same experiment, known as the i.i.d. regime, some effort was made to focus on single instances of information-theoretic tasks [51], where the natural entropy measures to consider are the *smooth entropies* [51–53]. In this regime, information erasure is also characterized using the smooth entropy framework [54–59].

The frameworks that have been considered for the study of the thermodynamics of information processing are extremely varying. While some studies have focused on explicit construction of physical systems, such as Szilard boxes [2, 55, 59], others have considered for example systems described by general Hamiltonians that are interacting, or for which we allow the modification of individual levels [14, 30, 46, 54, 56, 60–63]. Another very promising approach, from which the framework in this paper is largely inspired, is based on a resource theory of thermal operations, where the Gibbs states are for free [33, 57, 64–66]. The two approaches are equivalent [65].

Our result adds to the effort of relating information theory to thermodynamics, in the form of a general formula for the minimal work requirement of a logical process. The logical process can be any quantum physical evolution. This result may be seen as an information-theoretic result, which expresses the minimal size of an ancillary system needed to store the information discarded by the logical process, with a natural direct application to thermodynamics.

As a special case, our result allows us to study the work cost of quantum measurements. We start by noting that in the literature discussed above there is a slight ambiguity as to what a *measurement* is exactly, and, in particular, whether the memory register of the apparatus starts in a well-defined pure state or first needs initialization. If we include the memory initialization process, the measurement does cost work, whereas with a pure memory, the act of transferring information to the memory costs no work. This fact was also emphasized by Sagawa and Ueda [60, 67, 68], cf. also [69].

SUPPLEMENTARY NOTE 2 – SOME INITIAL REMARKS AND CLARIFICATIONS.

We wish to emphasize that in this entire work, and unless otherwise stated, by "process" we mean to denote a *logical* process, and not a thermodynamical process. Thermodynamical processes will come to play via the operations allowed by our framework.

Logical processes, or computations, are an abstract mathematical mapping of input states to output states. For example, and AND gate maps logical states `00`, `01` and `10` to the logical state `0` and `11` to the logical state `1`. Logical processes are defined completely independently of their physical implementation, in the same spirit of Shannon's abstraction of the unit of information. In the most general case, a logical process is specified by a completely

positive, trace-preserving map $\mathcal{E}$.

On the other hand, the logical information has to be stored on a physical system, and any logical operations have to be implemented through an appropriate time evolution of the physical system in interaction with a thermal bath and some control system(s). The specification of a logical process $\mathcal{E}$ contains no information about how much work was actually used to perform it—different strategies, different thermodynamical process or different levels of noise, losses or friction might cause the physical procedure use up very different amounts of work.

However, there is a fundamental limit on how much work will be *required* for the implementation of a logical process, or one could build a thermodynamic cycle with net work gain. In usual thermodynamics, this can simply be calculated as the difference in free energies between the final state and the initial state. In other words, the free energy, a state function, acts as a potential from which one can derive the minimal amount of work one needs in order to perform a transition from one thermodynamic state to another.

We derive the fundamental work requirement of implementing a logical process on the microscopic level. This is again the work cost of the *best possible thermodynamic process* that succeeds in implementing the given logical process. As mentioned in the main text, one of our main conclusions is that this minimal work requirement can no longer be given by a state function. In other words, it is not possible to define a "generalized free energy", a state function which would have the property of giving the minimal work requirement of a process as a difference between initial and final states of the computation. This is also in line with the conclusions of Lieb and Yngvason [70] as well as Horodecki *et al.* [57].

We wish to draw the attention of the reader to the fact that our conclusions have nothing to do with the statement that thermodynamic work itself is not a state function. Indeed, in standard thermodynamics and as mentioned above, the *minimal* work requirement for going from one state to another is still given by a state function, namely the free energy.

In order to further clarify the relation between logical and thermodynamical processes, consider an ideal gas of N particles in a box of volume $2V$, at temperature T. Let's consider bringing this gas to a new state with half the volume, given by the parameters (T, V, N) (see Supplementary Figure 1). The specification of a *logical* process goes beyond specifying the input and output states: indeed, one can require for example that the position of each particle be completely randomized at the end of the process (Supplementary Figure 1**a**), with no correlations between input and output; one can also require for example that a particle located at a position (x, y, z) be located at $(x/2, y, z)$ at the output (Supplementary Figure 1**b**). Both these logical processes have the same input and output state.

The first logical process can be physically implemented with a simple isothermal thermodynamical compression. The second logical process can be implemented similarly, by using a trick: first, we insert many separators in the box, resolving the positions of the particles to some acceptable precision, and then we perform an isothermal compression of each of those slices of the gas independently. Then each particle originally located at position x on the X-axis is now located at $x/2$ at the output and $kT \ln 2$ work was expended per particle. (Should the y and z coordinates also be required to be correlated between input and output, a grid of separators along the other axis should also be inserted, while the compression is performed in the X direction.) In these simple examples, both logical processes have the same minimal work cost (it is easy to see that the thermodynamical processes given above are optimal, for example using our main result). This is the illustration of our main result in the thermodynamic limit: in an i.i.d. setting, the minimal work requirement is simply given by the difference between final and initial entropy, *regardless of the specific logical process.* (Note that here entropy corresponds to free energy, since there is no change in internal energy.)

Note that both logical processes could have been performed with an irreversible thermodynamic process, for example a fast compression followed by a thermalization (of the whole gas in Supplementary Figure 1**a**, or of each slice in Supplementary Figure 1**b**). Then additional, irreversible work is required. However these irreversible processes are not the optimal thermodynamical processes that carry out the requested logical processes. The expression in our main result is given by the *optimal* thermodynamic implementation of a given logical process.

We further note that, in general, logically irreversible processes can be implemented in a thermodynamically reversible way: a Szilard box in a completely mixed state, for example, can be reset to a pure state by a simple reversible isothermal process. While the thermodynamic transformation is reversible, meaning that we can recover the initial mixed state and get back all the work invested in the erasure, the precise logical state the memory initially was in (whether the particle was on the left or the right side of the box) is irreversibly lost in the heat bath.(This reasoning does not contradict the results by Ladyman *et al.* [24], because the thermodynamical processes they show to be irreversible are the thermodynamic processes p_x "conditioned" on the particular initial logical state x of the device (using their notation).)

Consider now the example depicted in Supplementary Figure 1**c**: a single particle is in the box, and three partitions are inserted, subdividing the box into four even regions I, II, III and IV. We wish to perform the logical process that maps a particle in regions I, II and III to output region A, and input region IV to region B. (Upon appropriate relabeling of the regions, this is nothing else than an AND gate.) A thermodynamical operation that would carry out this logical mapping is an isothermal compression of the joint three first regions to one-third of their initial volume (removing the two intermediary separators). The work cost of doing so depends

on whether the particle is located in one of the regions I, II, III or not: with probability 3/4, $kT \ln 2 \cdot \log_2 3$ work is expended. (We assume that the isothermal process is done infinitely slowly, i.e. quasi-statically, such that the fluctuations have been reduced to zero; the fluctuations of the work cost of the logical process explained here are solely due to the probabilistic nature of the input state or the *logical* process, and not due to fluctuations of the *thermodynamical* process.) However, with probability 1/4, no work is expended as those regions are empty. In this case, the work cost is fluctuating due to the probabilistic nature of the input state. This procedure is again the best strategy we can devise if we require the process to succeed with almost certain probability (this is a consequence of our main result), and its worst-case work cost is $kT \ln 2 \cdot \log_2 3$.

If we had considered a large number of particles, then with large probability 3/4 of the particles would be in regions I, II, III, and thus the work yield would almost deterministically be the average value $3/4 \cdot kT \ln 2 \cdot \log_2 3 \approx 1.2\, kT \ln 2$, which one can check to be equal to the difference between initial and final Shannon entropy. This is again in agreement with our main result in the i.i.d. regime.

Now, let's consider again the AND gate with the one-particle gas, but where the particle has different probabilities of being in the different regions, as shown in Supplementary Figure 1**d**. Specifically, consider the example where the particle resides in region II or III with the same probability as it would be found in region IV, i.e. $p_{\mathrm{II}} + p_{\mathrm{III}} = p_{\mathrm{IV}}$. Again, we are required to perform the logical process mapping a particle in regions I, II or III to the region A, and mapping a particle in region IV to region B. Then, one can convince oneself that the optimal procedure is still to isothermally compress regions I, II, and III into the volume of region A, as before. However, as explained in the main text, it is possible to devise a strategy that will still reproduce the correct output state, $p_{\mathrm{A}} = p_{\mathrm{I}} + p_{\mathrm{II}} + p_{\mathrm{III}}$, $p_{\mathrm{B}} = p_{\mathrm{IV}}$, that will actually only cost $kT \ln 2$ work. This strategy corresponds to isothermally compressing regions II and III and merging them to region B, and isothermally compressing regions I and IV and merging them to region A (permuting regions and moving regions around does not cost any work, as these are unitary, reversible logical operations). This illustrates that the minimal work requirement of a logical process is not fully specified by the initial and final state. In particular, it cannot be given by a state function (such as the free energy in standard thermodynamics).

The important fact is that in macroscopic thermodynamics, one does not care about correlations between the input and the output, simply because for large i.i.d. systems (e.g. many independent particles, or large weakly interacting systems such as an ideal gas) those correlations do not matter. This is due to the system being in a typical microstate with overwhelming probability (see main text). We determine, for single quantum systems, the minimal work requirement of a logical process; our formula shows that this requirement is not simply given by a function of state. The key point is that if one goes to the thermodynamic limit, then our formula for the work cost of a logical process does become a function of state. This shows that our result is of a fundamentally different nature to the work loss of a process which is thermodynamically irreversible, which persists in the thermodynamical limit and is due to some avoidable irreversibility.

SUPPLEMENTARY NOTE 3 – THE SMOOTH ENTROPY FRAMEWORK.

The literature about information-theoretic tasks, pioneered by Shannon [71] has largely focused in the past on average resource costs of asymptotically many independent repetitions of a given task, such as the average communication rate needed to send the information output by a source generating independent messages according to a certain distribution.

Recently, frameworks were developed in order to characterize single instances of these tasks, such as determining how many bits are needed to compress a single message distributed according to some known distribution. The two major approaches are the information spectrum [72, 73] and the smooth entropy framework [51, 53], the two approaches being closely related [74].

We will focus here on the definition of some of the smooth entropies that are needed in this work and some of their properties. More information and proofs can be found in Refs. [51, 53, 75, 76].

In the remainder of this section, let A, B, C be quantum systems and let $|\rho\rangle_{ABC}$ be a pure tripartite state. We say that two states ρ_1 and ρ_2 are ε-close, denoted by $\rho_1 \approx_\varepsilon \rho_2$, if their *purified distance* as defined in Ref. [53, 75] is less than or equal to ε (for normalized states, the purified distance is defined via their fidelity [77]). We refer the reader to these papers for precise definitions of the purified distance, and for comprehensive discussions about optimization ranges over subnormalized states which will not be particularly relevant here.

Min and Max Entropies. The central quantities of the smooth entropy framework are the so-called *min-* and *max-entropies*. The conditional smooth min-entropy $H^{\varepsilon}_{\min}\left(A|B\right)_\rho$ is defined as follows:

$$H_{\min}\left(A|B\right)_{\rho|\sigma} = \max\left\{\lambda :\ 2^{-\lambda}\mathbb{1}_A \otimes \sigma_B \geqslant \rho_{AB}\right\}\ ; \quad (1a)$$

$$H_{\min}\left(A|B\right)_\rho = \max_{\substack{\sigma_B \geqslant 0 \\ \mathrm{tr}\,\sigma_B = 1}} H_{\min}\left(A|B\right)_{\rho|\sigma}\ ; \quad (1b)$$

$$H^{\varepsilon}_{\min}\left(A|B\right)_\rho = \max_{\hat\rho_{AB} \approx_\varepsilon \rho_{AB}} H_{\min}\left(A|B\right)_{\hat\rho}\ . \quad (1c)$$

Similarly, the smooth conditional max-entropy

$H^{\varepsilon}_{\max}(A|C)_\rho$ is defined by:

$$H_{\max}(A|C)_{\rho|\sigma} = \log F^2(\rho_{AC}, \mathbb{1}_A \otimes \sigma_C) \ ; \tag{2a}$$

$$H_{\max}(A|C)_\rho = \max_{\substack{\sigma_C \geqslant 0 \\ \operatorname{tr}\sigma_C = 1}} H_{\max}(A|C)_{\rho|\sigma} \ ; \tag{2b}$$

$$H^{\varepsilon}_{\max}(A|C)_\rho = \min_{\hat\rho_{AC} \approx_\varepsilon \rho_{AC}} H_{\max}(A|C)_{\hat\rho} \ . \tag{2c}$$

The conditional smooth entropies are invariant under local isometries. They also have clear operational interpretations [78]. For example, the min-entropy $H_{\min}(A|B)$ quantifies how many bits in A can be extracted that are uniformly random and uncorrelated to B; the max-entropy $H_{\max}(A|C)$ corresponds to the amount of bits needed to send to a third party who has access to C in order to reconstruct A.

Duality Relation. The min- and max-entropy obey the so-called *duality relation*. For ρ_{ABC} pure, one has

$$H^{\varepsilon}_{\min}(A|B)_\rho = -H^{\varepsilon}_{\max}(A|C)_\rho \ . \tag{3}$$

(The max-entropy may also be defined first by the duality relation, as originally done [78], and then (2) becomes a theorem.)

Classical-Quantum states. A state ρ_{AB} is classical-quantum (c-q) if it can be written in the form

$$\rho_{AB} = \sum_i p_i \, |i\rangle\langle i|_A \otimes \rho_B^{(i)} \ ,$$

for positive operators $\rho_B^{(i)}$ satisfying $\sum_i \operatorname{tr}\rho_B^{(i)} = 1$.

For such states, the conditional smooth entropies $H^{\varepsilon}_{\min}(A|B)_\rho$ and $H^{\varepsilon}_{\max}(A|B)$ are always positive.

Rényi-Zero Entropy. An additional entropy measure that will appear naturally in our calculations is the *Rényi entropy of order zero*, or the *Rényi-zero entropy* $H_0(A|C)_\rho$ [51, 79]. It is defined by

$$H_0(A|C)_\rho = \max_{\sigma_C} \operatorname{tr}[\Pi_{AC}\,\sigma_C] = \|\operatorname{tr}_A \Pi_{AC}\|_\infty \ , \tag{4}$$

where Π_{AC} is the projector onto the support of the state ρ_{AC}.

The Rényi-zero entropy is dual to a specific variant of the min-entropy: for ρ_{ABC} pure, one has

$$H_0(A|C)_\rho = -H_{\min}(A|B)_{\rho|\rho} \ . \tag{5}$$

The Rényi-zero entropy and this variant of the min-entropy have also been termed *alternative max-entropy* and *alternative min-entropy*, respectively [76].

When smoothed, the Rényi-zero entropy is closely related to the max entropy [76]. We have on one hand

$$H^{\varepsilon}_0(A|C)_\rho := \min_{\hat\rho \approx_\varepsilon \rho} H_0(A|C)_{\hat\rho} \geqslant H^{\varepsilon}_{\max}(A|C)_\rho \ , \tag{6}$$

and the two quantities are almost equal, up to an error term and a small adjustment $f(\varepsilon)$ to the smoothing parameter ε:

$$H^{f(\varepsilon)}_0(A|C)_\rho \leqslant H^{\varepsilon}_{\max}(A|C)_\rho + O\big(\log\tfrac{1}{\varepsilon}\big) \ . \tag{7}$$

Von Neumann entropy. Recall that the von Neumann entropy is defined as

$$H(X)_\rho = -\operatorname{tr}(\rho_X \log \rho_X) \ ; \tag{8a}$$

$$H(A|B)_\rho = H(AB)_\rho - H(B)_\rho \ . \tag{8b}$$

Asymptotic Equipartition Property. The smooth entropies all converge to the von Neumann entropy in the i.i.d. limit, a property which is known as *asymptotic equipartition* [52]. When considering n independent copies of the same state ρ, and consider large n, we have:

$$\lim_{\varepsilon\to 0}\lim_{n\to\infty} \frac{1}{n} H^{\varepsilon}_{\min}(A^n|B^n)_{\rho^{\otimes n}} = H(A|B) \ ; \tag{9a}$$

$$\lim_{\varepsilon\to 0}\lim_{n\to\infty} \frac{1}{n} H^{\varepsilon}_{\max}(A^n|B^n)_{\rho^{\otimes n}} = H(A|B) \ . \tag{9b}$$

In particular, any terms of order $\log\frac{1}{\varepsilon}$ disappear when taking the limit $n \to \infty$, such that the quantities $H^{\varepsilon}_0(A|B)_\rho$ and $H^{\varepsilon}_{\min}(A|B)_{\rho|\rho}$ also obey the asymptotic equipartition property.

SUPPLEMENTARY NOTE 4 – ADDITIONAL COMMENTS. APPLICATIONS OF OUR MAIN RESULT.

Our main result states that in our framework, the work cost of a physical process implementing the computation $\mathcal{E}$ exactly is lower bounded by the quantity $W^{\varepsilon=0}_{(\mathrm{bound})} = kT\ln 2 \cdot H_0(E|X')_\rho$. In the case where we consider an ε-approximation is tolerated, the bound takes the value $W^{\varepsilon}_{(\mathrm{bound})} = kT\ln 2 \cdot H^{\bar\epsilon}_{\max}(E|X')_\rho$, with $\bar\epsilon = \sqrt{2\varepsilon}$.

In the following sections, we further discuss the implications of our result and provide some examples.

In a slight abuse of notation, but in an effort to disencumber the mathematical expressions, a generic superscript ε on a work cost W or on an entropy measure will be understood to represent a "smoothing" of the quantity, in order to account for very unlikely events. It is understood that some expressions should actually contain variants of this quantity such as $\sqrt{2\varepsilon}$ to be technically correct, but our calculations being relatively simple, a technically complete version should be straightforward to obtain.

A. On the Tightness of the Minimal Work Bound.

Since the bound $W^{\varepsilon=0}_{(\mathrm{bound})}$ was obtained through a chain of equivalences from our original framework to the

expression of the bound, we know there exists a unital map over an "information battery" A and the system X which achieves this bound. However, it is not clear how to physically carry out a general unital operation at no work cost (remember: unital operations were precisely chosen for their being a very permissive framework, in order to obtain a more general bound). A convenient special case of unital operations are noisy operations [64]: these consist of a sequence of bringing in a maximally mixed ancilla, performing a global joint unitary, then tracing out the ancilla. However not all unital operations are of this kind [80, 81]. So this leaves open the question of whether our bound is tight.

We have seen in the main text that using the method proposed by del Rio *et al.* [54], we can construct an explicit process that carries out the required transformation, which fails with probability less than ε, and which costs work $kT\ln(2)\cdot\left[H^{\varepsilon}_{\max}\left(E|X'\right)_{\rho}+\Delta(\varepsilon)\right]$, where $\Delta(\varepsilon)$ is an error term of the order of $\log\left(1/\varepsilon\right)$. That is, we are capable of achieving the bound up to an error term of order $\log\left(1/\varepsilon\right)$.

In some interesting regimes, such as information coding, the error term may be negligible. Indeed, if we want to reset 1 MB of data with a smoothing parameter of at most $\varepsilon = 10^{-10}$, then the error term is of order of $\log\left(1/\varepsilon\right)\approx 30$ bits, which is small compared to the original $\sim 10^{7}$ bits. However, this error term can become overwhelming when considering small systems consisting of several qubits.

It is an open question to understand the significance of this gap, and to determine whether the bound can be exactly achieved. However, this type of error terms, which are clearly sublinear in the number of systems, are widespread in information and coding theory, and are typically associated with overheads such as encoding the word length itself, or the overhead of adapting the coding scheme. More specifically, they invariantly appear in random constructions of protocols, such as the one used in [54], on which our tightness proof is based.

B. Simple examples: the AND and XOR gates.

Consider the classical AND and XOR gates presented in the main text. We would now like to calculate how much work the best implementation of these gates would require. We can apply our main result, as given by Eq. (3) of the main text, to obtain

$$W^{(\text{bound})}_{\text{AND}} = kT\ln 2\cdot\log_2 3\approx 1.6\,kT\ln 2\ ; \tag{10a}$$

$$W^{(\text{bound})}_{\text{XOR}} = kT\ln 2\ . \tag{10b}$$

(Several quantum descriptions of these classical gates are possible in quantum mechanics; we assume for this example those which measure the input and prepare the appropriate output.)

As long as the input distribution does not have very small eigenvalues, no eigenvalues will be comparably small to ε, and all distributions that are ε-close to the initial one will have same rank. Thus the values (10) are exact also for not too large ε. (Note however that this differs with the expression in Eq. (1) of the main text, because the latter was obtained with an additional relaxation of H_0 to $H_{\max}$ for purposes of presentation.)

As mentioned in the main text, these gates illustrate the dependence of the minimal work requirement on the specific computation, and not only on the input and output states. More generally, it is worth noting that, although a specific input state is given, the observer can still distinguish the different possible logical processes, even though they give the same output state. Indeed, the observer can prepare a bipartite pure state on X and a reference R, with the reduced state on X matching the required input state. By keeping this way a purification of the input state, the observer can determine exactly which logical process was performed by appropriate measurements on the joint state $\rho_{X'R}$ of the output and the reference (note that $\rho_{X'R}$ is then the Choi-Jamiołkowski state of the logical process).

Observe also that the value (10a) differs from the average work requirement of the AND gate, which is given by the difference in von Neumann entropy between the input state and the output state (most previous work has focused on this regime). Assuming that the input is uniformly random, i.e. $\rho_X = \frac{1}{4}\mathbb{1}$, then one obtains

$$W_{\text{AND, avg.}} = kT\ln 2\cdot\left[H\left(X\right)-H\left(X'\right)\right]\approx 1.2\,kT\ln 2\ .$$

Additionally, the value (10b) happens to coincide with the average work requirement (calculated similarly) for a uniformly random input; however, if a different input is given, the two values will differ.

C. Arbitrarily large dependence on the computation, with same input and output states.

Consider the example provided in Supplementary Figure 2**a**,

$$\rho = \frac{1}{2}\left[|0\rangle\langle 0|\otimes|0\ldots 0\rangle\langle 0\ldots 0| + |1\rangle\langle 1|\otimes\frac{\mathbb{1}_{2^n}}{2^n}\right]\ .$$

Consider also the two logical processes depicted schematically in Supplementary Figure 2**b**. The first logical process $\mathcal{E}_1$ is the identity map, $\mathcal{E}_1 = \text{id}_{X\to X'}\left(\sigma\right) = \sigma$. The second logical process $\mathcal{E}_2$ resets its input and prepares a fresh copy of ρ, i.e. $\mathcal{E}_2\left(\sigma\right) = \text{tr}\left(\sigma\right)\,\rho$.

First note that both computations have exactly the same input and output states. The minimal work requirement of the identity mapping is zero, obviously, because it can be implemented by doing nothing, or also, because it is logically reversible. However, the analysis is different for $\mathcal{E}_2$. If we did nothing as for $\mathcal{E}_1$, then high correlations would remain between the input and the output, and we would not be implementing the computation $\mathcal{E}_2$ but

rather $\mathcal{E}_1$. Now the minimal work requirement that will be needed, if we want to be almost certain that the process succeeds, can be intuitively understood as follows: in the worst case, which happens with probability $1/2$, the input is in the state that is almost fully mixed, and one will first have to reset $\sim n$ bits, costing $\sim n\, kT \ln 2$ work. When preparing the output, we can decide randomly on whether to prepare the pure or the mixed state by extracting 1 bit of work from a Szilard box. However, in the worst case with probability $1/2$, we have to prepare the state $|0 \ldots 0\rangle$, and at worst only one bit of work can be extracted. The total worst-case work cost of this strategy is

$$W^{\varepsilon\approx 0}(\mathcal{E}_2, \rho) \approx n\, kT \ln 2\ . \tag{11}$$

This value can be calculated exactly as our example is a special case of subsection 4 G below; it turns out to be optimal. The approximation we made above (where we note '$\sim$' and '$\approx$') is simply that $\log(2^n + 1) \approx n$ and $n + 1 \approx n$. Also, we have assumed that $(1-\varepsilon)\, n \approx n$.

Note that the quantity (11) can become arbitrarily large, as it scales with the number of qubits n.

This distribution might seem very artificially constructed. We however provide here an example of a physical system which exhibits such behavior. Consider a particle detector, which we model in the following way: as long as no particle has shown up, the detector is initialized in a state $|0\rangle$. Once a particle hits the device, the state of the detector is changed to a very disordered state τ, which we may for the sake of the example choose as a uniformly mixed state of rank d: $\tau = \frac{1}{d}\mathbb{1}_d$. Suppose we wish to describe the state of the device, not knowing whether a particle has hit it or not. If the probability that a particle was detected is $1/2$, then the state of the detector is precisely ρ (with $d = 2^n$).

"Erasure" here simply means resetting the device to its initialized state: the logical process maps the distribution ρ to the pure state $|0\rangle$. The first logical processes given in Supplementary Figure 2**b** corresponds to not doing anything to the detector. The second logical process corresponds to resetting the detector, and then again sending a new particle in with probability $1/2$. Note that in this case, by looking at the detector after the process, we may not know the state of the detector at the input of the process.

D. Erasure of a Quantum System Using a Quantum Memory.

This scenario was studied in the main text; we repeat this derivation in more detail here.

Consider the setting proposed in [54], where a system S is correlated with a system M in a joint state σ_{SM}, and where our task is to erase S while preserving the reduced state on M and any possible correlations of M with other systems. Formally, given a purification σ_{SMR} of σ_{SM}, we are looking for a process that will bring this state to the state $\rho_{SMR} = |0\rangle\langle 0|_S \otimes \sigma_{MR}$, i.e. we require the process to preserve σ_{MR}. In [54] a process is proposed that performs this task at work cost

$$kT \ln(2)\, H^{\varepsilon}_{\max}\,(S|M)_{\sigma} \;+ O\big(\log \tfrac{1}{\varepsilon}\big)\ ,$$

where $H^{\varepsilon}_{\max}$ is the smooth max entropy [53, 75, 78].

The full process that is eventually performed can be written as

$$\mathcal{E}^{(\text{erasure})}_{SM\to SM}\,(\sigma) = |0\rangle\langle 0|_S \otimes \operatorname{tr}_S\,(\sigma)\ . \tag{12}$$

(It is straightforward to verify that this process preserves the reduced state σ_{MR}.) We can now apply our main result to this particular mapping, simply by considering X to be the joint system of S and the memory M, $\mathscr{H}_X = \mathscr{H}_S \otimes \mathscr{H}_M$. Note that we have $\rho_{SMR} = |0\rangle\langle 0|_S \otimes \sigma_{MR}$, purified by $|\rho\rangle_{SMRE} = |0\rangle_S \otimes |\rho\rangle_{MRE}$, where $|\rho\rangle_{MRE} = U_{S\to E}\,|\sigma\rangle_{SMR}$ and $U_{S\to E}$ is an isometry from S to E.

Then the bound on the work cost, including a smoothing parameter ϵ, is

$$\begin{aligned} W &\geqslant H^{\varepsilon}_{\max}(E|SM)_{\rho} \cdot kT \ln(2) \\ &= H^{\varepsilon}_{\max}(E|M)_{\rho} \cdot kT \ln(2) \\ &= H^{\varepsilon}_{\max}(S|M)_{\sigma} \cdot kT \ln(2)\ , \end{aligned} \tag{13}$$

where the first equality follows because ρ is pure on S and the second by reversing the isometry U. We can immediately conclude that, within our framework, any process that performs this erasure has to cost at least $kT \ln(2)\, H^{\varepsilon}_{\max}(S|M)_{\sigma}$ work. Thus, the process proposed by del Rio *et al.* is optimal up to logarithmic factors in ε. Note that if we take the memory M to be trivial i.e. a pure state, then we are in the standard scenario of Landauer erasure on a single system, and we have $W \geqslant H^{\varepsilon}_{\max}(S)$ which is achievable, recovering the result of [55].

E. Coherent Preparation of a State on a System with a Memory. "Reverse" of a Logical Process.

One may also wonder which process is the "reverse process" of erasure with a quantum memory. Specifically, starting off with a pure system S and some state ρ_M on a memory M, one might ask how much work is needed to prepare a given bipartite state σ_{SM} on these systems.

The process as such is not clearly defined, as we have not specified which correlations between the initial and final state on M are to be preserved, or, equivalently, which completely positive map $\mathcal{E}$ is to be applied for this preparation.

Let us first study the erasure mapping (12) a bit more closely. The output state of the erasure including the reference system R is given by the state $\rho_{X'R} = |0\rangle\langle 0|_S \otimes \rho_{MR}$, where $\mathscr{H}_{X'} = \mathscr{H}_S \otimes \mathscr{H}_M$ is the total output system. As mentioned earlier, the joint state on X' and R may be interpreted as the *process matrix* of the operation $\mathcal{E}$ on

σ: it can be thought of as a joint probability distribution giving the probability that we had $|k\rangle$ at the input and got $|k'\rangle$ at the output; also, $\rho_{X'}$ is the output state and ρ_R is the input state. This consideration gives us a natural way of *reversing* any process: a natural "reverse" process to the process $\rho_{X'R}$ is simply given by swapping the two systems, i.e. considering R as the output and X' as a purification of the input. Let us return to the case of the erasure. First, consider the purification of σ_{SM} into a system $\mathscr{H}_R = \mathscr{H}_{R_S} \otimes \mathscr{H}_{R_M}$ explicitly as

$$|\sigma\rangle_{SMR_SR_M} = \sigma_{SM}^{1/2}|\Phi\rangle_{SM|R_SR_M} \ , \tag{14}$$

where $|\Phi\rangle_{SM|R_SR_M} = \sum_{kl}|k\rangle_S|k\rangle_{R_S}|l\rangle_M|l\rangle_{R_M}$. Now applying the erasure process on SM gives us:

$$\begin{aligned}\rho_{SMR_SR_M} &= \mathcal{E}^{(\text{erasure})}_{SM\to SM}\left(\sigma_{SMR_SR_M}\right)\\ &= |0\rangle\langle 0|_S \otimes \operatorname{tr}_S\left(\sigma_{SMR_SR_M}\right) \ .\end{aligned} \tag{15}$$

It is thus natural, for the preparation scenario, to consider the process matrix

$$\rho^{(\text{prep})}_{SMR_SR_M} = |0\rangle\langle 0|_{R_S} \otimes \operatorname{tr}_{R_S}\left(\sigma_{SMR_SR_M}\right) \ . \tag{16}$$

(Recall that the input state is pure on S.)

This is obviously purified by a system E which contains the traced out information, i.e. given an isometry $U_{R_S\to E}$,

$$|\rho^{(\text{prep})}\rangle_{SMR_SR_ME} = |0\rangle_{R_S} \otimes \left(U_{R_S\to E}\,|\sigma\rangle_{SMR_SR_M}\right) \ . \tag{17}$$

If we then calculate the minimal work cost of performing this process according to our main result, we obtain

$$\begin{aligned}W^{(\text{prep})} &= kT\ln 2 \cdot H^{\varepsilon}_{\max}\left(E|SM\right)_{\rho^{(\text{prep})}}\\ &= kT\ln 2 \cdot H^{\varepsilon}_{\max}\left(R_S|SM\right)_{\sigma}\\ &= -kT\ln 2 \cdot H^{\varepsilon}_{\min}\left(R_S|R_M\right)_{\sigma}\\ &= -kT\ln 2 \cdot H^{\varepsilon}_{\min}\left(S|M\right)_{\sigma} \ .\end{aligned} \tag{18}$$

(We have used the fact that σ and ρ are related by an isometry between R_S and E, as well as the duality between min- and max-entropies.)

We notice that $kT\ln 2 \cdot H_{\min}\left(S|M\right)_{\sigma}$ work can be extracted in the reverse process of the original erasure process, which required $kT\ln 2 \cdot H_{\max}\left(S|M\right)_{\sigma}$ work. These values can be arbitrarily different; this gap is expected as we require both processes to succeed with high probability. We find that the gap is exactly the difference between the min- and the max-entropy, similarly to the single-shot irreversibility between distillation rate and formation rate of entangled pairs with LOCC operations [82–85].

F. The Minimal Work Cost of a Quantum Measurement.

Quantum measurements are special cases of quantum processes, and so may also be plugged into our main result. Note that first, we consider the measurement process to be given access to a pure memory register to store the measurement result. We then consider the minimal work cost of preparing the register in its pure state again for a future measurement.

The Measurement Process and its Work Cost.—Suppose that on the system S, in the state σ, we perform a measurement described by a POVM $\{Q_k\}$. Each outcome, labeled by the index k, occurs with probability $\operatorname{tr}\left(Q_k\sigma\right)$. The completely positive map associated with this measurement is,

$$\mathcal{E}_{S\to S'C}\left(\sigma\right) = \sum_k |k\rangle\langle k|_C \otimes \mathcal{E}^{(k)}_{S\to S'}\left(\sigma\right) \ , \tag{19}$$

where C is a classical register containing the outcome of the measurement (initially in a pure state), and $\mathcal{E}^{(k)}_{S\to S'}\left(\cdot\right) = \sum_i E^{(k)}_i\left(\cdot\right)E^{(k)\,\dagger}_i$ are trace-decreasing maps that map σ to its post-measurement state for the outcome k, which occurs with probability $\operatorname{tr}\left(Q_k\sigma\right) = \operatorname{tr}\mathcal{E}^{(k)}_{S\to S'}\left(\sigma\right)$. The Kraus operators $E^{(k)}_i$ are related to the POVM elements Q_k by $\sum_i E^{(k)\,\dagger}_i E^{(k)}_i = Q_k$. Equation (19) simply expresses that the output state of the measurement is a mixture of the possible post-measurement states corresponding to the different outcomes k. We emphasize that the register C must start off in a pure state; if this is not the case (as in a purity resource framework, for example), it should be initialized first, causing some work cost, before performing the map $\mathcal{E}$.

We first need to calculate the Stinespring dilation of the process $\mathcal{E}$, which is given by $\mathcal{E}_{S\to CS'}\left(\cdot\right) = \operatorname{tr}_E V_{S\to ECS'}\left(\cdot\right)V^{\dagger}$, where the isometry $V_{S\to ECS'}$ can be read off the operator-sum representation of (19), that is

$$\mathcal{E}_{S\to CS'} = \sum_{k,i}\left(|k\rangle_C \otimes E^{(k)}_i\right)\sigma\left(\langle k|_C \otimes E^{(k)\,\dagger}_i\right) \ , \tag{20}$$

as $V = \sum_{k,i}|k,i\rangle_E \otimes |k\rangle_C \otimes E^{(k)}_i$. This yields the post-measurement state including E

$$\rho_{ECS'} = V_{S\to ECS'}\ \sigma_S\ V^{\dagger} \ .$$

For convenience, let R be a purifying system for σ_S, i.e. let $|\sigma\rangle_{SR}$ such that $\operatorname{tr}_R|\sigma\rangle\langle\sigma|_{SR} = \sigma_S$. This allows us to write a full, pure, post-measurement state $|\rho\rangle_{ECS'R}$ as

$$|\rho\rangle_{ECS'R} = \sum_{k\,i}|k,i\rangle_E \otimes |k\rangle_C \otimes \left(E^{(k)}_i|\sigma\rangle_{SR}\right) \ . \tag{21}$$

Our main result asserts that the minimal work cost of

the measurement (19) is simply given by the quantity,

$$W^{\varepsilon}_{\text{meas,optimal}} \approx kT \ln 2 \cdot H^{\varepsilon}_{\max}\left(E|CS'\right) \ , \tag{22}$$

where the entropy measure is evaluated for the state $\rho_{ECS'R}$ given by (21). In the remainder of this section, all entropy measures are implicitly evaluated on this state, unless indicated otherwise. The '≈' symbol recalls that one can only actually approximately achieve the given bound (see Section 4 A). In the remainder of this section, when discussing optimal work costs, we will only consider the value of our bound; it is understood that a successful implementation is possible at a work cost close to the discussed bound in the sense of Section 4 A. It is also implied that all work costs are smoothed with a small but finite ε parameter. In this spirit, denote the value of the right-hand side of (22) by

$$W_{\text{meas}} = kT \ln 2 \cdot H^{\varepsilon}_{\max}\left(E|CS'\right) \ . \tag{23}$$

As we will see from some simple examples, the quantity (19) in its most general form as presented may take any value, from a work cost to a work yield. However, we will consider an important class of measurements: those for which the collapse operators $\mathcal{E}_k$ don't themselves need work. In general, we know that those processes that don't need work are sub-unital, i.e. they satisfy $\mathcal{E}^{(k)}\left(\mathbb{1}\right) \leqslant \mathbb{1}$. This is for example the case for projective measurements, or more generally if the $\mathcal{E}^{(k)}$'s only have a single Kraus operator. We also note that any general measurement in the form (19) can be written as a combination of a measurement with collapse superoperators that have each a single Kraus operator, followed by a partial erasure on the memory register C.

Proposition 1. *Let $\mathcal{E}_{S\to CS'}$ be a measurement process of the form (19), and assume that for all k, $\mathcal{E}^{(k)}\left(\mathbb{1}\right) \leqslant \mathbb{1}$. Then W_{meas} as defined by (23), satisfies $W_{meas} \leqslant 0$.*

Proof. Instead of proving that the entropic quantity (23) is negative, we will show that the full measurement process $\mathcal{E}$ itself is a sub-unital superoperator, which we know from our framework costs no work. This is straightforward to see:

$$\begin{aligned}\mathcal{E}_{S\to CS'}\left(\mathbb{1}_S\right) &= \sum_k |k\rangle\langle k|_C \otimes \mathcal{E}^{(k)}_{S\to S'}\left(\mathbb{1}_S\right) \\ &\leqslant \sum_k |k\rangle\langle k|_C \otimes \mathbb{1}_{S'} \leqslant \mathbb{1}_{CS'} \ . \qquad \square\end{aligned}$$

Resetting the Memory Register Containing the Measurement Outcome.—Let us now consider the task of resetting C to a pure state, after having performed the measurement process above. This resetting can obviously be performed directly, with a cost given by Landauer's principle as $H^{\varepsilon}_{\max}\left(C\right)_{\rho}$, which in turn depends on the number of possible outcomes the measurement had (if $\varepsilon > 0$, we would only consider the measurements that are not extremely unlikely). This procedure, however, is not optimal if we are allowed access for example the information contained in the post-measurement state on S'. Indeed, in the latter case, we may use the system S' as a memory as discussed in Sec. 4 D, and the optimal work cost is then

$$W_{\text{reset } C|S'} = kT \ln 2 \cdot H^{\varepsilon}_{\max}\left(C|S'\right)_{\rho} \ . \tag{24}$$

This work cost is always positive, or at best, zero, because $\rho_{CS'}$ is classical-quantum (c-q).

We will see that this work cost may be both less and larger than W_{meas} with some examples. Of course, this does not constitute a violation of the second law, as we will discuss.

Additionally, we could imagine a scenario where we have kept a purification of the input state $|\sigma\rangle_{SR}$ on an ancilla system R, in order to "remember" the state of the initial system S. We may then of course use this system also to reduce the work cost of erasing C, the latter being

$$W_{\text{reset } C|R} = kT \ln 2 \cdot H^{\varepsilon}_{\max}\left(C|R\right) \ .$$

It turns out that, if the collapse operators $\mathcal{E}^{(k)}$ all have a single Kraus operator, this work cost is always greater than the work yield of performing the measurement ($-W_{\text{meas}}$), and that the difference between both is precisely the difference between the max and min-entropy of the system C conditioned on R.

Proposition 2. *Let $\mathcal{E}_{S\to CS'}$ be a measurement process of the form (19), with for all k, $\mathcal{E}^{(k)}\left(\cdot\right) = E_k\left(\cdot\right)E_k^{\dagger}$. Let $\rho_{ECS'R}$ be defined as in (21). Then*

$$\begin{aligned}&W_{\text{meas}} + W_{\text{reset } C|R} \\ &\qquad = kT \ln 2 \cdot \left[-H^{\varepsilon}_{\min}\left(C|R\right) + H^{\varepsilon}_{\max}\left(C|R\right)\right]\end{aligned}$$

Additionally, if ε is not too large (such that $H^{\varepsilon}_{\max} \geqslant H^{\varepsilon}_{\min}$ [86]), this expression is always positive,

$$W_{\text{meas}} + W_{\text{reset } C|R} \geqslant 0 \ .$$

Proof. First notice that the state $\rho_{ECS'R}$ takes the form

$$|\rho\rangle_{ECS'R} = \sum_k |k\rangle_E |k\rangle_C \left(E_k|\sigma\rangle_{SR}\right) \ ,$$

and in particular, ρ is invariant under interchange of E and C systems. Then, using duality of the smooth entropies [75] (see Section 3), we have $H^{\varepsilon}_{\max}\left(E|CS'\right) = -H^{\varepsilon}_{\min}\left(E|R\right) = -H^{\varepsilon}_{\min}\left(C|R\right)$ and thus $W_{\text{meas}} = -kT \ln 2 \cdot H^{\varepsilon}_{\min}\left(C|R\right)$. Then recall that $W_{\text{reset } C|R} = kT \ln 2 \cdot H^{\varepsilon}_{\max}\left(C|R\right)$ and that the max-entropy is larger than the min-entropy for small ε. $\square$

The final (pure) state on E, C, S' and R, which is the output of the Stinespring isometry V applied on the S system of $|\sigma\rangle_{SR}$, is still given by the expression (21).

Some Examples of Measurement Processes.—Let us now focus on some examples of measurement processes, which

are all special cases of (19).

(I) *Measurement in the computational basis* $|0\rangle$, $|1\rangle$ *of a single qubit in a maximally mixed state* $\mathbb{1}_2/2$. The measurement process then simply yields the output state

$$\rho_{CS'} = \frac{1}{2}\,|0\rangle\langle 0|_C \otimes |0\rangle\langle 0|_{S'} + \frac{1}{2}\,|1\rangle\langle 1|_C \otimes |1\rangle\langle 1|_{S'} \ .$$

The input state on S is purified by a fully entangled state $|\phi\rangle_{SR}$ on R. The system E also purifies the measurement process, and as given by (21),

$$\begin{aligned} |\rho\rangle_{ECS'R} &= \sum_k |k\rangle_E |k\rangle_C \ (|k\rangle\langle k|_S |\phi\rangle_{SR}) \\ &= \frac{1}{\sqrt{2}}\left[|0\rangle_C|0\rangle_E|00\rangle_{SR} + |1\rangle_C|1\rangle_E|11\rangle_{SR}\right] \ . \end{aligned}$$

It is then evident that

$$\begin{aligned} W_{\text{meas}} &= kT\ln 2 \cdot H^{\varepsilon}_{\max}\left(E|CS'\right) = 0 \ ; \\ W_{\text{reset } C|S'} &= kT\ln 2 \cdot H^{\varepsilon}_{\max}\left(C|S'\right) = 0 \ ; \\ W_{\text{reset } C|R} &= kT\ln 2 \cdot H^{\varepsilon}_{\max}\left(C|R\right) = 0 \ , \end{aligned}$$

as the corresponding reduced states are all classically correlated.

(II) *Measurement of a trivial noisy POVM.* Consider a POVM in the extreme case where the state is left untouched, but a random outcome is generated according to a distribution $\{p_k\}$. The POVM effects are simply $Q_k = p_k\mathbb{1}$ and the post-measurement operators $\mathcal{E}^{(k)}(\sigma) = p_k\sigma$ are simply the identity superoperator weighted by the probability p_k.

Intuitively, this should be no different than rolling a die, or more generally, generating a random outcome with a specific distribution, which is a process that can yield work. Indeed, based on the explicit expression of the final state

$$|\rho\rangle_{ECS'R} = \Big(\sum_k \sqrt{p_k}\,|k\rangle_E|k\rangle_C\Big) \otimes |\sigma\rangle_{S'R} \ , \tag{25}$$

we may express of the work cost of the measurement using some basic properties of the smooth entropies, presented in Section 3. Using the duality of the min- and max-entropies,

$$\begin{aligned} W_{\text{meas}}/(kT\ln 2) &= H^{\varepsilon}_{\max}\left(E|CS'\right) = -H^{\varepsilon}_{\min}\left(E|R\right) \\ &= -H^{\varepsilon}_{\min}\left(E\right) = -H^{\varepsilon}_{\min}\left(C\right) \leqslant \log\|\rho_C\|_\infty \leqslant 0 \ , \end{aligned}$$

because R is not correlated to E, and ρ is invariant under exchange of E and C (both these statements can be seen in (25)), and by definition the min-entropy of C is given by $H_{\min}(C) = -\log\|\rho_C\|_\infty$. Also, smoothing the min-entropy can only increase the quantity by its definition (1c).

One can also calculate, because C is uncorrelated to both S' and R,

$$\begin{aligned} W_{\text{reset } C|S'} &= kT\ln 2 \cdot H^{\varepsilon}_{\max}\left(C|S'\right) = kT\ln 2 \cdot H^{\varepsilon}_{\max}\left(C\right) \ ; \\ W_{\text{reset } C|R} &= kT\ln 2 \cdot H^{\varepsilon}_{\max}\left(C|R\right) = kT\ln 2 \cdot H^{\varepsilon}_{\max}\left(C\right) \ . \end{aligned}$$

This means that the work we need to invest to reset C is always larger than what we gain from generating the random outcome. In fact, the gap is precisely the difference between the max- and the min-entropy, which is the same kind of irreversibility that is observed between the single-shot entanglement distillation and formation cost between two parties [87].

(III) *Projective measurement of a pure superposition state.* One may think that intuitively, for the measurement to yield work, the POVM must be noisy. Surprisingly enough, this is not the case. Even projective measurements can yield work for specific input states. For example, consider the state $|\sigma\rangle_S = |+\rangle := \frac{1}{\sqrt{2}}(|0\rangle + |1\rangle)$. Here R is a trivial system since σ is already pure. Now consider the usual projective measurement that measures σ in the computational basis $|0\rangle$, $|1\rangle$. The final state is

$$|\rho\rangle_{ECS'} = \frac{1}{\sqrt{2}}\left(|0\rangle_E|0\rangle_C|0\rangle_{S'} + |1\rangle_E|1\rangle_C|1\rangle_{S'}\right) \ .$$

We then evidently have

$$\begin{aligned} W_{\text{meas}} &= kT\ln 2 \cdot H^{\varepsilon}_{\max}\left(E|CS'\right) = -kT\ln 2 \ ; \\ W_{\text{reset } C|S'} &= kT\ln 2 \cdot H^{\varepsilon}_{\max}\left(C|S'\right) = 0 \ ; \\ W_{\text{reset } C|R} &= kT\ln 2 \cdot H^{\varepsilon}_{\max}\left(C|R\right) \\ &= kT\ln 2 \cdot H^{\varepsilon}_{\max}\left(C\right) = kT\ln 2 \ . \end{aligned}$$

We conclude that it is possible to extract one bit of work while performing the measurement, and that resetting the memory register can be done at no work cost using S' but needs one bit of work if we use the (trivial) reference system R.

Note that resetting the measurement register C using S' costs no work. This is not in violation of the second law of thermodynamics: we have not returned the post-measurement state back to the initial state, but rather we have consumed its purity.

(IV) *Measurement with erasure collapse operators.* It was noted above that if the collapse operators $\mathcal{E}^{(k)}$ were themselves maps that cost work, e.g. erasure channels, then the measurement would also possibly cost work. It is sufficient to consider the following extreme example: take a single-outcome measurement, i.e. a trivial measurement, with a the single collapse operator $\mathcal{E}_{k=0}(\cdot) = \mathrm{tr}(\cdot)\,|0\rangle\langle 0|$ being an erasure channel. Obviously this operation has to

cost work: performing operation $\mathcal{E}_{S\to CS'}$ is exactly the same as performing just the erasure $\mathcal{E}_{k=0}$, which costs work according to our main result (which is of course also in line with Landauer's principle).

(V) *Information Gain of a Measurement.* Existing literature [88–91] has studied and identified the amount of information that a quantum measurement provides about a system being measured. With the notation above, the information gain in the asymptotic, i.i.d. regime is defined in [90] as

$$\iota\left(\sigma_S, \mathcal{E}\right) = I\left(R : C\right)_\rho := H\left(R\right) - H\left(R|C\right) \ . \tag{26}$$

In our framework, information contained in a quantum system is represented by how much work we need in order to erase that system. Bearing this in mind, the natural way of defining the amount of information gained about the system using the measurement is then the difference in work costs of erasing S *before* and *after* the measurement. Since S was consumed by the measurement, this statement doesn't fully make sense, so we will rather consider erasing the system R instead, which is a purification of S. Our take at the information gain of the measurement is then

$$\iota' = H^\varepsilon_{\max}\left(R\right) - H^\varepsilon_{\max}\left(R|C\right) \ .$$

Notice that in the i.i.d. regime where the entropies converge to the von Neumann entropy, this definition coincides with the previous one (26).

G. State Transformation while Decoupling from the Reference System.

Let's return to another special case that we can derive as a corollary from our main result. Consider the process that erases its input and prepares the required output independently. This would occur if we required the output state to be completely uncorrelated to the reference system R: $\rho_{X'R} = \rho_{X'} \otimes \rho_R$. This corresponds to a replacement map. Any third party R that would have been correlated to the input is now completely uncorrelated to the output.

Again, we may simply apply our main result with the additional condition that $\rho_{X'R} = \rho_{X'} \otimes \rho_R$. In this case, the purification of $\rho_{X'R}$, $\rho_{X'RE}$, takes a special form due to the tensor product structure, with the E system split into two E_R and $E_{X'}$ systems ($E = E_R \otimes E_{X'}$),

$$|\rho\rangle_{X'RE} = |\psi\rangle_{X'E_{X'}} \otimes |\phi\rangle_{RE_R} \ , \tag{27}$$

where $|\psi\rangle_{X'E_{X'}}$ and $|\phi\rangle_{RE_R}$ are purifications of $\rho_{X'}$ and ρ_R, respectively.

The (smooth) lower bound on the minimal work cost W, given by our main result, then reads

$$\begin{aligned} &W \geqslant kT \ln 2 \cdot H^\varepsilon_{\max}\left(E|X'\right)_\rho \\ &= kT \ln 2 \cdot H^\varepsilon_{\max}\left(E_R\right)_{|\phi\rangle} + kT \ln 2 \cdot H^\varepsilon_{\max}\left(E_{X'}|X'\right)_{|\psi\rangle} \ . \end{aligned}$$

Now, the spectrum of ρ_{E_R} is exactly the same as the spectrum of ρ_R by the Schmidt decomposition of $|\phi\rangle$. This in turn has the same spectrum as σ_X also by the Schmidt decomposition of σ_{XR} and because $\rho_R = \sigma_R$. It follows that $H^\varepsilon_{\max}(E_R)_\rho = H^\varepsilon_{\max}(X)_\sigma$. Also, by duality of the min- and max-entropies, we have $H^\varepsilon_{\max}\left(E_{X'}|X'\right)_{|\psi\rangle} = -H^\varepsilon_{\min}\left(E_{X'}\right)_\rho = -H^\varepsilon_{\min}\left(X'\right)_\rho$. In consequence,

$$W \geqslant kT \ln 2 \cdot \left[H^\varepsilon_{\max}\left(X\right)_\sigma - H^\varepsilon_{\min}\left(X\right)_\rho\right] \ . \tag{28}$$

That is, to transform a state σ to ρ while completely decorrelating ρ from the input, then one has to erase σ to a pure state (at cost $H^\varepsilon_{\max}\left(X\right)_\sigma$), and then prepare ρ (extracting work $H^\varepsilon_{\min}\left(X'\right)_\rho$).

H. Example: Erasing Part of a W State. Again, the Importance of Correlations Between the Input and the Output.

Consider the W state on a system S, a memory M and a reference system R given by

$$|W\rangle_{SMR} = \frac{1}{\sqrt{3}}\left[|001\rangle + |010\rangle + |100\rangle\right]_{SMR} \ . \tag{29}$$

The reduced states on SM and M are respectively given by $\sigma_{SM} = \frac{1}{3}|00\rangle\langle 00| + \frac{2}{3}|\Psi^+\rangle\langle\Psi^+|$ and $\sigma_M = \frac{2}{3}|0\rangle\langle 0| + \frac{1}{3}|1\rangle\langle 1|$, where $|\Psi^+\rangle = \frac{1}{\sqrt{2}}\left(|01\rangle + |10\rangle\right)$. By symmetry of the W state, the reduced state on any two or one qubit(s) have the same form.

By actions on S and M, we would like to erase S, leading to the final state on S and M given by $\rho_{SM} = |0\rangle\langle 0| \otimes \sigma_M$. Let us consider two processes that achieve this goal: the first one will preserve correlations with R but will cost work, the second will not cost work but will modify those correlations.

We may directly apply the special case above concerning the erasure of a system conditioned on a memory: the fundamental work cost of such an erasure, if one preserves correlations with a reference system R, is given by $H_0\left(S|M\right)_\sigma$. In this case we have $H_0\left(S|M\right)_\sigma = \log\frac{2}{3} \approx 0.59$ (which we calculate below) and thus this process must cost at least this amount of work. Because of the small system size, we may not assert the achievability of this erasure at this work cost (the error terms discussed in Section 4 A become overwhelming). However we can safely exclude the possibility of performing this operation at no work cost, a statement which suffices for our purposes here.

Observe now that both σ_{SM} and σ_M have the same spectrum $\{2/3, 1/3\}$. This means that there exists a uni-

tary U that performs the erasure simply as $|0\rangle\langle 0|\otimes\sigma_M = U\sigma_{SM}U^\dagger$, and this unitary process by definition does not cost any work. Note though that the correlations with R are not preserved. Indeed, the unitary sends $|00\rangle$ to $|01\rangle$ and $|\Psi^+\rangle$ to $|00\rangle$, so one explicitly calculates that the state after the process is given by $\rho_{SMR} = U\sigma_{SMR}U^\dagger = \frac{1}{\sqrt{3}}\left[|011\rangle + \sqrt{2}|000\rangle\right] = |0\rangle\otimes\frac{1}{\sqrt{3}}\left[|11\rangle + \sqrt{2}|00\rangle\right]$. We notice that the reduced state on M and R is now pure and differs from initial one, given by $\sigma_{MR} = \frac{1}{3}|00\rangle\langle 00| + \frac{2}{3}|\Psi^+\rangle\langle\Psi^+|$.

As before, this is an example where one can transform the input state into the output state at no work cost a priori, but if correlations are to be preserved between the memory and a reference system or, equivalently, if the exact erasure process (12) is to be performed, then a physical implementation of this operation would require work.

It remains to calculate $H_0\left(S|M\right)_\sigma$. Written out explicitly in the basis $\{|0\rangle, |1\rangle\}$, the state σ_{SM} and the projector Π_{SM} on its support take the form

$$\sigma_{SM} = \begin{pmatrix} 1/3 & & & \\ & 1/3 & 1/3 & \\ & 1/3 & 1/3 & \\ & & & 0 \end{pmatrix} \quad ; \quad \Pi_{SM} = \begin{pmatrix} 1 & & & \\ & 1/2 & 1/2 & \\ & 1/2 & 1/2 & \\ & & & 0 \end{pmatrix} .$$

(Empty entries are zero.) We then see that

$$\mathrm{tr}_S\,\Pi_{SM} = \begin{pmatrix} 3/2 & \\ & 1/2 \end{pmatrix} ,$$

such that

$$H_0\left(S|M\right)_\sigma = \log\left\|\mathrm{tr}_S\,\Pi_{SM}\right\|_\infty = \log\frac{3}{2} .$$

SUPPLEMENTARY NOTE 5 – ALTERNATIVE PROOF USING LAMBDA-MAJORIZATION.

For completeness, we provide an alternative proof of our main result, based on techniques of majorization and semidefinite programming. This proof (historically, the original one) ventures via the study of possible state transitions, regardless of the logical process, but then imposes that the resulting logical process be the one required.

A. The Framework. Work cost or yield as generating or absorbing randomness.

Framework.—Consider a quantum mechanical system X in an initial state described by the density operator σ. Our task is to bring the system X to another state ρ, while attempting to maximize some kind of notion of "extracted" work in the process.

We postulate a restricted set of operations as possible physical processes which we may carry out. Throughout this paper we assume that the system starts and ends with a fully degenerate Hamlitonian upon each application of an allowed operation. There is no further restriction, however, on how each of the allowed operations themselves are implemented—they might require a time-dependent Hamiltonian for example.

We first postulate two basic operations of thermodynamical nature, involving a heat bath at temperature T: the erasure of a single qubit to a pure state at $kT\ln(2)$ work cost, and the corresponding reverse process which extracts $kT\ln(2)$ work by transforming a pure state into a fully mixed state. Here k is the Boltzmann constant. These operations are motivated by the variety of explicit physical thermodynamical frameworks in which they can be performed, for example using Szilard boxes [2, 55] or by isothermally manipulating energy levels of Hamiltonians [30, 54, 56]. Crucially, we assume the second law of thermodynamics, and require that there exist no operation that would allow us to form a cycle for which the net effect would be the extraction of work. This justifies that no other work extraction procedure can yield more work than $kT\ln(2)$ from a pure qubit, or else a cycle with net work gain could be formed by appending an erasure process, itself only costing $kT\ln(2)$.

Apart from this constraint on the set of allowed operations, it is natural to also allow usual quantum information processing. Since our Hamiltonians are degenerate, we can allow all global unitaries and they cost no work. We do not need to use the fact that these unitaries are implementable by a device operating in contact with a heat bath, since expanding the class of allowable operations actually strengthens the bound we derive. In practice, one has very crude local control over the operations, and the acting agent does not know which unitary is being implemented, however, this is actually not an obstacle for implementation [65, 92]. In addition to unitaries, we will allow pure ancillas to be added to the system, which permits more general computation. Crucially, ancillas will have to be exactly restored to their initial pure state, so that it is not possible to "hide" a work cost in an ancilla that was left in a mixed state.

The following framework is motivated by the above considerations. The processes we allow are (finite) combinations of the following elementary operations:

(a) Bring n qubits (of the system X or an ancilla A) from any state to a pure state ('erasure') at cost $n\,kT\ln 2$ work;

(b) Bring n qubits (of the system X or an ancilla A) from a pure state to a fully mixed state while extracting $n\,kT\ln 2$ work;

(c) Add and remove ancillas in a pure state at no work cost, as long as all the ancillas have been restored to their initial pure state when they are restored;

(d) Perform arbitrary unitaries (over X and any added ancillas) at no work cost.

Operations (a) and (b) are those of thermodynamical nature, and may be carried out in a wide range of existing

frameworks as mentioned above. One may view these operations as *defining* a quantity which we call "work". We note that these operations can be performed quasistatically in a thermodynamically reversible fashion (as long as operation (a) acts on a fully mixed state, which in fact will turn out to be sufficient for our purposes).

On the other hand, operations (c) and (d) are purely information-theoretical. They allow us to perform any quantum information processing circuit, since we allow pure ancillas to be added. However, there is the condition that "randomness" may not be disposed of for free, namely that ancillas have to be restored to their initial pure states at the end of the process.

We emphasize that these operations are *allowed* operations, but they are not necessarily always optimal. For example, a pure state need not require $n\,kT\ln 2$ for its erasure, as given by operation (a). However, any attempt to allow operation (a) for any state (or even just for a mixed state) at any lower cost than $n\,kT\ln 2$ would result in a macroscopic violation of the second law of thermodynamics.

Lambda-Majorization.—We will now provide a simple mathematical characterization of all operations allowed in our framework.

First, note that the operations (a)–(d) allow the use of so-called *noisy operations* [64], which correspond to adding an ancilla system N in a fully mixed state, performing a joint unitary, and removing the ancilla. Specifically, a noisy operation is composed in our framework of first an operation of type (c) (adding a pure ancilla of n qubits), followed by an operation of type (b) (extracting $n\,kT\ln 2$ work from the ancilla making it fully mixed), then one of type (d) (performing the necessary unitary to carry out the noisy operation), and finally an operation of type (a) (erasing the ancilla back to its pure state at a work cost $n\,kT\ln 2$). (It can be assumed without loss of generality that the ancilla is left in a fully mixed state after the noisy operation; indeed, this is the case for the construction of the noisy operation given by Ref. [64], which is capable of performing an equivalent transformation to any other noisy operation.) The total process has a work balance of zero. This means that we may thus carry out noisy operations for free within our framework and use them as building blocks for more complex processes. In the following, we deal implicitly with the ancilla N and it should not be confused with further ancillas that will be added.

The following result by Horodecki *et al.* [64] relates noisy operations to the mathematical notion of majorization [93–95].

Noisy Operations and Majorization. *The transition on system X from state σ to state ρ is possible by noisy operation if and only if $\sigma \succ \rho$.*

Majorization between two (normalized) states $\sigma \succ \rho$ captures the fact that ρ is "more mixed" than σ, or that the eigenvalues of ρ can be written as a "mixture" of the eigenvalues of σ. Formally, majorization can be characterized by the existence of a unital, trace-preserving completely positive map that brings σ to ρ [96–99]. A map $\mathcal{E}$ is *trace-preserving* if $\mathcal{E}^\dagger(\mathbb{1}) = \mathbb{1}$ and *unital* if $\mathcal{E}(\mathbb{1}) = \mathbb{1}$.

Proposition 3. *Two positive matrices σ and ρ satisfy $\sigma \succ \rho$ if and only if there exists a trace-preserving, unital, completely positive map $\mathcal{E}$ satisfying $\mathcal{E}(\sigma) = \rho$.*

The notion of majorization is discussed in more detail in Section 6.

We will now provide some background insight for our new concept of lambda-majorization, which is a generalization of majorization inspired by other majorization variants [57, 59, 100–103]. The idea is to characterize "how well" a state σ majorizes a state ρ. Suppose that we have a system X in state σ_X and we want to bring it to the state ρ_X, where $\sigma_X \succ \rho_X$. In this case, one can simply carry out a noisy operation as described above. Suppose now that we have an ancilla A that is in a fully mixed state, $\frac{\mathbb{1}_A}{|A|}$, and suppose that we are fortunate enough for $\sigma_X \otimes \frac{\mathbb{1}_A}{|A|} \succ \rho_X \otimes |0\rangle\langle 0|_A$ to also hold (for some pure state $|0\rangle_A$ on A). Then by applying a joint noisy operation on both systems, this would correspond to actually erasing the system A "for free" during the transition $\sigma \to \rho$. We could then say that the randomness of the ancilla A was "transferred" into system X. We will view this type of transition as *work extraction* on system X during a transition $\sigma_X \to \rho_X$. Indeed, work can be extracted in an initial stage of the process by starting with a pure ancilla and making it maximally mixed; the operation described above costs no work and the ancilla can then be restored in its pure final state.

In another situation, it might be that $\sigma_X \not\succ \rho_X$. However, in that case, for a large enough ancilla A the majorization $\sigma_X \otimes |0\rangle\langle 0|_A \succ \rho_X \otimes \frac{\mathbb{1}_A}{|A|}$ will hold. The corresponding noisy operation then leaves us with a mixed ancilla that started off pure and thus requires work to restore; we will view such a transition on system X as *costing work.*

Such operations can be performed within our framework, using operations (a)–(d). In particular, the relation to work is given by elementary erasure and work extraction (operations (a) and (b)) applied to the ancilla A after the transition to restore it to its initial state.

In general, the ancilla A may start with λ_1 mixed qubits and end up with λ_2 mixed qubits after a noisy operation; we consider in this case to have extracted $(\lambda_1 - \lambda_2)\,kT\ln(2)$ amount of work. This situation is depicted in Supplementary Figure 3**a**. Both considerations above about work cost and work extraction are encompassed, simply because we count the difference in the "amount of randomness" present in the ancilla before and after the process. This is the idea behind the concept of lambda-majorization, whose definition we can now state.

Lambda-Majorization. *For two density operators σ_X, ρ_Y on two systems X and Y, we will say that σ_X λ-majorizes ρ_Y, denoted by $\sigma_X \xrightarrow{\lambda} \rho_Y$, if there exists a*

(large enough) ancilla system A, as well as $\lambda_1, \lambda_2 \geqslant 0$ with $\lambda = \lambda_1 - \lambda_2$, such that

$$2^{-\lambda_1}\mathbb{1}_{2^{\lambda_1}} \otimes \sigma_X \succ 2^{-\lambda_2}\mathbb{1}_{2^{\lambda_2}} \otimes \rho_X \ ,$$

where $2^{-\lambda_1}\mathbb{1}_{2^{\lambda_1}}$ and $2^{-\lambda_2}\mathbb{1}_{2^{\lambda_2}}$ are fully mixed states on λ_1 (respectively λ_2) qubits of A, and where the remaining qubits of A in each case are pure.

An expression for "by how much" a state majorizes another was originally introduced in [58] and used in [59], in the context of work extraction games from Szilard boxes. Their measure, the "relative mixedness" between σ and ρ, corresponds to the optimal λ such that $\sigma \xrightarrow{\lambda} \rho$.

Lambda-majorization captures all the possible processes that are allowed in our framework. Indeed, if $\sigma \xrightarrow{\lambda} \rho$, then one has $2^{-\lambda_1}\mathbb{1}_{2^{\lambda_1}} \otimes \sigma \succ 2^{-\lambda_2}\mathbb{1}_{2^{\lambda_2}} \otimes \rho$ for some λ_1, λ_2 with $\lambda = \lambda_1 - \lambda_2$. Hence, there exists a noisy operation (itself a combination of operations (a)–(d) with zero total work cost) that performs the transition from $2^{-\lambda_1}\mathbb{1}_{2^{\lambda_1}} \otimes \sigma$ to $2^{-\lambda_2}\mathbb{1}_{2^{\lambda_2}} \otimes \rho$. The λ_1 mixed qubits that we have appended to σ can be created by appending a large pure ancilla (operation (c)), and using operation (b) to extract $\lambda_1\, kT\ln(2)$ work from λ_1 qubits, rendering them fully mixed. At the end of the process, after the noisy operation, we need to restore the ancilla in a pure state; we thus need to erase (operation (a)) the remaining λ_2 qubits, costing $\lambda_2\, kT\ln(2)$ work. The total extracted work is then $(\lambda_1 - \lambda_2)\, kT\ln(2) = \lambda\, kT\ln(2)$. Conversely, each individual operation (a)–(d), individually transforming some state σ into a state ρ and costing work W, implies the lambda-majorization $\sigma \xrightarrow{\lambda} \rho$ with $W = -\lambda\, kT\ln(2)$. This is clear for operations (c) and (d). For operations (a) and (b), this follows from results derived in Section 6 C. Furthermore, the composition of lambda-majorizations is again a lambda-majorization (Section 6).

The ancilla system above may be viewed as some kind of "information battery", as was proposed by Bennett [7] who suggested using a blank memory tape as "fuel" to extract work. In this case, the ancilla can be used as a storage of "purity" (or as a storage for "mixedness" or "randomness" which we would like to get rid of), which is increased or decreased by processes like the ones suggested above. Equivalently, a two level system, or *work bit* can be used [57].

It turns out that one can characterize lambda-majorization by the existence of a completely positive map satisfying some special normalization conditions, analogously to Proposition 3.

Proposition 4. *Let $\lambda \in \mathbb{R}$. Two normalized density matrices σ_X and ρ_Y on two systems X and Y satisfy $\sigma_X \xrightarrow{\lambda} \rho_Y$ if and only if there exists a completely positive map $\mathcal{T}_{X\to Y}$ satisfying $\rho_Y = \mathcal{T}_{X\to Y}(\sigma_X)$, such that $\mathcal{T}^{\dagger}_{X\to Y}(\mathbb{1}_X) \leqslant \mathbb{1}_Y$ and $\mathcal{T}_{X\to Y}(\mathbb{1}_X) \leqslant 2^{-\lambda}\mathbb{1}_Y$.*

A map $\mathcal{T}_{X\to Y}$ that satisfies the two last conditions will be referred to as a *lambda-majorization map.*

Furthermore, although the map $\mathcal{T}$ is not directly a physical mapping (it can be, for example, trace-decreasing), it can always be viewed as part of a unital channel $\bar{\mathcal{E}}$, in the sense that $\mathcal{T}$ can be obtained by projection onto specific subspaces and tracing out the ancilla A of the map $\bar{\mathcal{E}}$ (see Section 6 B). In turn, unital channels are a (strict [80]) superset of the noisy operations. Recall that our task is to find a lower bound on the work cost of all possible processes allowed in our framework, which we will do by optimizing the work cost over all processes that perform a given state transition. However, instead of considering only the unital channels $\bar{\mathcal{E}}$ that are noisy operations, we will relax this last condition and consider all unital maps $\bar{\mathcal{E}}$, and thus allow the optimization to range over all $\mathcal{T}$ that satisfy the conditions of the above proposition. This will make our lower bound even stronger, by showing that the lower bound still holds even if we relax somewhat the assumptions in our framework.

B. The Main Result.

1. Formulation and Proof Sketch.

Formulation of the Main Result.—We are now ready to derive our main result. Consider a system X in the state σ_X. This system can always be purified by a reference system, R, in a pure joint state $|\sigma\rangle_{XR}$.

Allowing actions defined by our framework on X, we will study the transition of this state to a state ρ_{XR}, by applying a process $\mathcal{T}_{X\to X'}$. The systems are depicted in Supplementary Figure 3**b**.

The task we would like to solve is the following. Given σ_X and a logical process $\mathcal{E}_{X\to X'}$, and given a purification $|\sigma\rangle_{XR}$ of σ_X and an output state $\rho_{X'R} = \mathcal{E}(\sigma_{XR})$, we would like to find the least amount of work W one has to pay for any process in our framework that implements the action of $\mathcal{E}$ on σ. As we have seen in the previous section, we can formulate within our framework all possible processes as lambda-majorizations, so our task is actually to find the best λ such that $\sigma_X \xrightarrow{\lambda} \rho_{X'}$, with the corresponding lambda-majorization map $\mathcal{T}$ from Prop. 4 satisfying $\mathcal{T}(\sigma_{XR}) = \rho_{X'R}$.

Our main result gives an upper bound on the optimal amount of work that can be extracted by this transition, or equivalently, a lower bound on the minimum amount of work that will have to be paid in order to perform the transition. The main result follows directly from following technical proposition.

We are given an input state σ_X and a process $\mathcal{E}_{X\to X'}$. Let $|\sigma\rangle_{XR}$ be a purification of σ_X, and let $\rho_{X'R} = \mathcal{E}_{X\to X'}(\sigma_{XR})$. Let also $\rho_{X'RE}$ be a purification of $\rho_{X'R}$ in a system E.

Proposition 5. *The λ-majorization $\sigma_X \xrightarrow{\lambda} \rho_{X'}$ holds, with the corresponding map $\mathcal{T}_{X\to X'}$ from Prop 4 satisfying $\mathcal{T}(\sigma_{XR}) = \rho_{X'R}$, if and only if $\lambda \leqslant -H_0(E|X')_\rho$.*

Main Result. *Any procedure in our framework acting on system X that implements the map $\mathcal{E}$ when given input σ_X (or equivalently, that brings the state σ_{XR} to the state $\rho_{X'R}$) has a work cost W which is at least*

$$W \geqslant kT \ln(2) \cdot H_0 \left(E|X'\right)_\rho \ . \tag{30}$$

In other words, *the minimal work cost of a process $\mathcal{E}$ mapping σ to ρ is given by the amount of (information-theoretic) entropy discarded, and thus dumped into the environment, conditioned on the output of the computation.* This is precisely the quantitative generalization to correlated quantum systems of the original Landauer's principle [5].

The Main Result follows from Prop. 5 because, as we have noted above, lambda-majorization is equivalent to our original framework of operations (a)–(d).

It is worth noting that instead of specifying the map $\mathcal{E}$, we may also simply specify the output state $\rho_{X'R}$, which completely determines the process (on the support of σ_X) since it is the Choi-Jamiołkowski state corresponding to $\mathcal{E}$ rescaled by σ_X ($\rho_{X'R} = \mathcal{E}\left(\sigma_{XR}\right)$). One can thus understand the input to the problem to actually be a bipartite state $\rho_{X'R}$, such that $\rho_{X'}$ is the required output, ρ_R is the input that will be fed into the process, and any correlations between X' and R specify parts of the output that we wish be preserved and not be modified, or thermalized, by the process.

We have kept above the notation X' consistently to remember that we are talking about the output of the computation on X. However, X' could be a different system entirely. It could even have a different dimension than X, however in this case there are some clarifications needed: whenever the system dimension increases, pure ancillas have been brought in and haven't necessarily been restored to their pure state since they are part of the output; however this operation need not have cost any work (in contrast to other noisy operations resource theories, where purity is costly [64, 65]). However, whenever the system dimension decreases, then any ancilla that was removed had to be reset to a pure state first before being disposed of, which may have cost work. In other words, we adhere to the convention where purity can be brought in for free, but where disposing of randomness is costly; this is equivalent to the other approach where purity is costly but disposing of mixed states is done for free. Our choice is *a priori* arbitrary but possesses the advantage of well integrating into our mathematical framework with simple mathematical descriptions in terms of subunital maps and weak sub-majorization (see Section 6).

The full proof of Prop. 5 is provided in Section 5 B 2. We provide the general idea of the proof in the following.

Proof Sketch of the Main Result.—The main idea of the proof is to write the optimization problem as a semidefinite program for the variables $\alpha = 2^{-\lambda} \geqslant 0$, $T_{XX'} \geqslant 0$ (the Choi-Jamiołkowski representation of $\mathcal{T}_{X\to X'}$). Let $(\cdot)^{t_X}$ denote the partial transpose operation on X. Consider the state transformation $\sigma \to \rho$. An upper bound on the extracted work λ in the lambda-majorization $\sigma \xrightarrow{\lambda} \rho$, while ensuring that the map $\mathcal{T}$ from Prop. 4 performs the same logical operation as $\mathcal{E}$, is given by the following semidefinite program (see [104, 105] for a introduction to SDPs in a style similar to what we use here.):

Primal

`minimize:` α

`subject to:`

$$\mathcal{T}_{X\to X'}\left(\mathbb{1}_X\right) \leqslant \alpha \mathbb{1}_{X'}$$
$$\mathcal{T}^\dagger_{X\leftarrow X'}\left(\mathbb{1}_{X'}\right) \leqslant \mathbb{1}_X$$
$$\mathcal{T}_{X\to X'}(\sigma_{XR}) = \rho_{X'R}\ .$$

The optimal value $\alpha = 2^{H_0(E|X')_\rho}$ is achieved (see Section 5 B 2) by the completely positive map $\mathcal{T}_{X\to X'} = \mathrm{tr}_E\left[V_{X\to X'E}\left(\cdot\right)V^\dagger\right]$, where $V_{X\to X'E}$ is the partial isometry with minimal support relating σ_{XR} to $\rho_{X'ER}$ (both being purifications of the same $\sigma_R = \rho_R$).

While it is clear from the formulation of our problem that $\mathcal{T}$ is already completely determined on the support of σ_X (expressed by the condition $\mathcal{T}\left(\sigma_{XR}\right) = \rho_{X'R}$), the optimization over $\mathcal{T}$ is done in order to (at least formally) find the optimal action on the complement of the support of σ_X.

Also, the formulation of a lambda-majorization problem as a semidefinite program is a more general toolbox that could be used in the case where the mapping is not completely determined and where arbitrary additional semidefinite conditions can be imposed at will. For example, instead of fixing the process with $\mathcal{T}\left(\sigma_{XR}\right) = \rho_{X'R}$, one may have instead required that $\mathcal{T}\left(\sigma_X\right) = \rho_{X'}$ for given σ_X and $\rho_{X'}$, not specifying and optimizing over what happens to correlations between the input and the output (or, equivalently, one could optimize over $\rho_{X'R}$ with fixed reductions $\rho_{X'}$ and ρ_R). In that case, the semidefinite program can be used to obtain bounds to the optimal value. This also implies that the "relative mixedness" introduced in [59] can be formulated as a semidefinite program. However it is not clear if the result in this case can be written in terms of an entropy measure.

2. *Proof of the Main Result. Formulation as a Semidefinite Program.*

Let $\mathscr{H}_X$ be a quantum system in the state σ_X. Let $\mathscr{H}_R$ be an additional quantum system and let $|\sigma\rangle_{XR}$ be a purification of σ_X.

Suppose we want to perform the computation $\mathcal{E}_{X\to X'}$ on system X, bringing its initial state σ_{XR} into a given state $\rho_{X'R}$ with a lambda-majorization. Here $\rho_{X'R}$ is not necessarily pure; giving the joint state with R allows us to specify which correlations we want to preserve, equivalently specifying the computation $\mathcal{E}$ on the support of σ_X. The task is then the following.

Task. Find the best (maximal) λ, such that there exists a completely positive, $2^{-\lambda}$-subunital, trace-nonincreasing map $\mathcal{T}_{X\to X'}$ satisfying $\mathcal{T}_{X\to X'}(\sigma_{XR}) = \rho_{X'R}$.

In other words, we would like to find the trace nonincreasing map that satisfies $\mathcal{T}_{X\to X'}(\sigma_{XR}) = \rho_{X'R}$, that has the smallest possible $\|\mathcal{T}_{X\to X'}(\mathbb{1}_X)\|_\infty$.

This problem can be formulated as a semidefinite program in terms of the variables $\alpha \geqslant 0$ (defined as $\alpha = 2^{-\lambda}$) and $\mathcal{T}_{X\to X'}$ (through its Choi-Jamiołkowski map $T_{XX'} \geqslant 0$), and the dual variables $\omega_{X'} \geqslant 0$, $X_X \geqslant 0$ and $Z_{X'R} = Z_{X'R}^\dagger$.

Primal

`minimize:` α

`subject to:`

$$\mathrm{tr}_X\left[T_{XX'}\right] \leqslant \alpha \mathbb{1}_{X'} \quad : \omega_{X'} \tag{32a}$$

$$\mathrm{tr}_{X'}\left[T_{XX'}\right] \leqslant \mathbb{1}_X \quad : X_X \tag{32b}$$

$$\mathrm{tr}_X\left[T_{XX'}\sigma_{XR}^{t_X}\right] = \rho_{X'R} \quad : Z_{X'R} \tag{32c}$$

Dual

`maximize:`

$$\mathrm{tr}\left(Z_{X'R}\,\rho_{X'R}\right) - \mathrm{tr}\,X_X$$

`subject to:`

$$\mathrm{tr}\,\omega_{X'} \leqslant 1 \tag{33a}$$

$$\mathrm{tr}_R\left[\sigma_{XR}^{t_X} Z_{X'R}\right] \leqslant \mathbb{1}_X \otimes \omega_{X'} + X_X \otimes \mathbb{1}_{X'} \ . \tag{33b}$$

Note that since the map does not act on σ_R, we must necessarily have $\sigma_R = \rho_R$. Let E be a system that purifies the output state as $\rho_{X'RE}$. As two purifications with the same reduced state on R, the two states σ_{XR} and $\rho_{X'RE}$ must be related by an isometry $V_{X\to X'E}$ as $\rho_{X'RE} = V_{X\to X'E}\,\sigma_{XR}\,V^\dagger$. Note that this is an equivalent construction of the Stinespring dilation of the original computation $\mathcal{E}_{X\to X'}$. We can choose here $V_{X\to X'E}$ to be a partial isometry such that $VV^\dagger = \hat{\Pi}_{X'E}$, the projector on the support of $\rho_{X'E}$, and $V^\dagger V = \Pi_X$, the projector on the support of σ_X.

Now, define $\mathcal{T}$ by its Stinespring dilation

$$\mathcal{T}_{X\to X'}(\cdot) = \mathrm{tr}_E\left[V_{X\to X'E}\,(\cdot)\,V^\dagger\right] \ , \tag{34}$$

and let $\alpha = \|\mathcal{T}(\mathbb{1}_X)\|_\infty$. In fact, $\mathcal{T}$ is simply a projection on the support of σ_X followed by the mapping $\mathcal{E}$. We will show that this choice of variables is feasible and optimal, and will derive a more explicit value of α.

Condition (32a) is satisfied by definition and (32b) because V is a partial isometry. Also, verifying condition (32c),

$$\mathcal{T}_{X\to X'}(\sigma_{XR}) = \mathrm{tr}_E\left[V_{X\to X'}\,\sigma_{XR}V^\dagger\right] = \mathrm{tr}_E\,\rho_{X'RE} = \rho_{X'R} \ . \tag{35}$$

Now calculate

$$\alpha = \|\mathcal{T}(\mathbb{1}_X)\|_\infty = \|\mathrm{tr}_E\,VV^\dagger\|_\infty = \|\mathrm{tr}_E\,\hat{\Pi}_{X'E}\|_\infty = \max_{\tau_{X'}} \mathrm{tr}\left[\hat{\Pi}_{X'E}\,\tau_{X'}\right] = 2^{H_0(E|X')_\rho} \ . \tag{36}$$

We will now show that this value is optimal by exhibiting a solution to the dual problem that achieves the same value. Let $\omega_{X'} = \tau_{X'}$ be the optimal $\tau_{X'}$ for the definition of $H_0(E|X')$ as in (36), let $Z_{X'R} = \sigma_R^{-1} \otimes \omega_{X'}$ and let $X_X = 0$. This choice is feasible since condition (33a) is automatically satisfied and condition (33b) becomes

$$\begin{aligned}\mathrm{tr}_R\left[\sigma_{XR}^{t_X} Z_{X'R}\right] &= \mathrm{tr}_R\left[\sigma_{XR}^{t_X}\cdot \rho_R^{-1}\otimes\omega_{X'}\right] \\ &= \mathrm{tr}_R\left[\Phi_{X|R}^{t_X}\otimes\omega_{X'}\right] \\ &= \Pi_X^{t_X}\otimes\omega_{X'} \leqslant \mathbb{1}_X\otimes\omega_{X'} \ ,\end{aligned} \tag{37}$$

where $\Phi_{X|R}$ is an unnormalized maximally entangled state on the supports of σ_X and σ_R. Let $\rho_{X'RE}$ and $V_{X\to X'E}$ be defined as before. The value achieved by this choice of dual variables is then

$$\mathrm{tr}\left[Z_{X'R}\,\rho_{X'R}\right] = \mathrm{tr}\left[\sigma_R^{-1}\otimes\omega_{X'}\cdot\rho_{X'R}\right] \tag{38}$$

$$= \mathrm{tr}\left[\sigma_R^{-1}\otimes\omega_{X'}\cdot V_{X\to X'E}\,\sigma_{XR}V^\dagger\right] \tag{39}$$

$$= \mathrm{tr}\left[\omega_{X'}\cdot V_{X\to X'E}\,\Phi_{X|R}V^\dagger\right]$$

$$= \mathrm{tr}\left[\omega_{X'}\hat{\Pi}_{X'E}\right] = 2^{H_0(E|X')_\rho} \ . \tag{40}$$

From this, we conclude that the optimal λ for this problem is

$$\lambda_{\mathrm{opt}} = -H_0(E|X')_\rho \ . \tag{41}$$

where $\rho_{X'RE}$ is a purification of $\rho_{X'R}$.

SUPPLEMENTARY NOTE 6 – FORMAL TOOLS FROM MAJORIZATION AND LAMBDA-MAJORIZATION

A. Preliminaries and Main Definition

Let $\mathscr{H}_X$, $\mathscr{H}_Y$ be two subspaces of a finite-dimensional Hilbert space $\mathscr{H}_Z$, and let $\mathscr{H}_A$, $\mathscr{H}_B$ be two subspaces of a finite-dimensional Hilbert space $\mathscr{H}_C$. Let $d_{(\cdot)}$ denote the dimensions of the various Hilbert spaces $\mathscr{H}_{(\cdot)}$ and specifically let $d = d_Z = \dim\mathscr{H}_Z$. Denote by $\mathscr{L}(\mathscr{H})$ the set of linear hermitian operators on $\mathscr{H}$, by $\mathscr{P}(\mathscr{H})$ the set of positive semidefinite operators on $\mathscr{H}$, and by $\mathscr{S}_=(\mathscr{H})$ those operators in $\mathscr{P}(\mathscr{H})$ that have unit trace. Let also $\lambda_i(\rho)$ denote the i-th eigenvalue of ρ (the order doesn't matter), and $\lambda_i^\downarrow(\rho)$ denote the i-th eigenvalue of ρ taken in decreasing order.

Majorization is discussed in detail in Refs. [94, 95, 106].

Majorization. *A matrix $\sigma \in \mathscr{P}(\mathscr{H}_Z)$ is said to majorize $\rho \in \mathscr{P}(\mathscr{H}_Z)$, denoted by $\sigma \succ \rho$, if for all k,*

$\sum_{i=1}^{k}\lambda_i^{\downarrow}(\sigma) \geqslant \sum_{i=1}^{k}\lambda_i^{\downarrow}(\rho)$, *and if* $\operatorname{tr}\sigma = \operatorname{tr}\rho$.

The notion of majorization defines a (partial) order relation on $\mathscr{P}(\mathscr{H}_Z)$. When considering the set of density matrices $\mathscr{S}_=(\mathscr{H}_Z)$, there is a "least" element: the fully mixed state, $\frac{1}{d}\mathbb{1}_Z$.

Weak Submajorization. *A matrix* $\sigma \in \mathscr{P}(\mathscr{H}_X)$ *is said to* weakly submajorize $\rho \in \mathscr{P}(\mathscr{H}_Y)$, *denoted by* $\sigma \succ_w \rho$, *if for all* k, $\sum_{i=1}^{k}\lambda_i^{\downarrow}(\sigma) \geqslant \sum_{i=1}^{k}\lambda_i^{\downarrow}(\rho)$.

Remark that if $\sigma, \rho \in \mathscr{S}_=(\mathscr{H}_Z)$, then the concept of weak submajorization is equivalent to regular majorization simply because the traces of these matrices are already equal to unity.

Doubly Stochastic Matrix. *A* $d\times d$ *matrix* S *is* doubly stochastic *if* ${S_i}^j \geqslant 0$, $\sum_i {S_i}^j = 1\ \forall\, j$ *and* $\sum_j {S_i}^j = 1\ \forall\, i$.

Doubly Substochastic Matrix. *A* $n \times m$ *matrix* B *is* doubly substochastic *if* ${B_i}^j \geqslant 0$, $\sum_i {B_i}^j \leqslant 1\ \forall\, j$ *and* $\sum_j {B_i}^j \leqslant 1\ \forall\, i$.

The following theorem is due to Hardy, Littlewood and Pólya [93].

Theorem 6 (Hardy, Littlewood, and Pólya, 1929)**.** *Let* $\sigma, \rho \in \mathscr{P}(\mathscr{H}_Z)$. *Then* $\sigma \succ \rho$ *if and only if there exists a* $d \times d$ *doubly stochastic matrix* ${S_i}^j$ *such that* $\lambda_i(\rho) = \sum_j {S_i}^j \lambda_j(\sigma)$.

A similar theorem is obtained for weak submajorization and doubly substochastic matrices [94].

Proposition 7. *Let* $\sigma \in \mathscr{P}(\mathscr{H}_X)$ *and* $\rho \in \mathscr{P}(\mathscr{H}_Y)$. *Then* $\sigma \succ_w \rho$ *if and only if there exists a* $d_Y \times d_X$ *doubly substochastic matrix* ${B_i}^j$ *such that* $\lambda_i(\rho) = \sum_j {B_i}^j \lambda_j(\sigma)$.

Majorization defines a partial order on states and has a "smallest" element, the fully mixed state. Also, a pure state majorizes any other state.

Proposition 8. *Majorization is preserved by direct sums and tensor products, i.e. if* $\sigma \succ \rho$ *and* $\sigma' \succ \rho'$, *then* $\sigma \oplus \sigma' \succ \rho \oplus \rho'$ *and* $\sigma \otimes \sigma' \succ \rho \otimes \rho'$. *The same holds for weak submajorization.*

A proof for the direct sum of two vectors can be found in [94, Cor. II.1.4]. We provide here an alternative proof along with the tensor product case.

Proof. Let S_i^j and $S_i'^j$ be doubly stochastic matrices such that $\lambda_i(\rho) = \sum_j S_i^j \lambda_j(\sigma)$ and $\lambda_i(\rho') = \sum_j S_i'^j \lambda_j(\sigma')$. Then $S \oplus S'$ is also doubly stochastic and satisfies $\lambda_i(\rho \oplus \rho') = \sum_j (S \oplus S')_i^j \lambda_j(\sigma \oplus \sigma')$, because the vectors of eigenvalues of the direct sum are simply the direct sums of the individual vector of eigenvalues. This shows that $\sigma \oplus \sigma' \succ \rho \oplus \rho'$.

Analogously, $S \otimes S'$ satisfies $\lambda_{ii'}(\rho \otimes \rho') = \lambda_i(\rho)\lambda_{i'}(\rho') = \sum_{jj'} S_i^j \lambda_j(\sigma) S_{i'}^{j'} \lambda_{j'}(\sigma') = \sum_{jj'} (S \otimes S')_{ii'}^{jj'} \lambda_{jj'}(\sigma \otimes \sigma')$. $S \otimes S'$ is doubly stochastic, $\sum_{ii'} (S \otimes S')_{ii'}^{jj'} = \sum_{ii'} S_i^j S_{i'}^{j'} = 1$ and $\sum_{jj'} (S \otimes S')_{ii'}^{jj'} = \sum_{jj'} S_i^j S_{i'}^{j'} = 1$.

The same proof holds for doubly substochastic matrices, so majorization may be replaced by weak submajorization in the proposition. ☐

We are now all set for a formal definition of lambda-majorization.

Let $\lambda \in \mathbb{R}$ and let $\lambda_1, \lambda_2 \geqslant 0$ such that $\lambda = \lambda_1 - \lambda_2$ and 2^{λ_1}, 2^{λ_2} are integers. (The case when 2^λ is irrational will be discussed later.) Take $\mathscr{H}_C$ of size greater than both 2^{λ_1} and 2^{λ_2} and let $\mathscr{H}_A$ and $\mathscr{H}_B$ be subspaces of $\mathscr{H}_C$ of respective dimensions 2^{λ_1} and 2^{λ_2}.

Lambda-Majorization. *For* $\sigma \in \mathscr{P}(\mathscr{H}_X)$ *and* $\rho \in \mathscr{P}(\mathscr{H}_Y)$, *we say that* σ λ-majorizes ρ, *denoted by* $\sigma \xrightarrow{\lambda} \rho$, *if there exists such* λ_1, λ_2 *such that* $2^{-\lambda_1}\mathbb{1}_A \otimes \sigma \succ_w 2^{-\lambda_2}\mathbb{1}_B \otimes \rho$. *Here* $\mathbb{1}_A$, $\mathbb{1}_B$ *are the projectors onto the respective subspaces* $\mathscr{H}_A$ *and* $\mathscr{H}_B$ *embedded in* $\mathscr{H}_C$, *of respective dimensions* 2^{λ_1}, 2^{λ_2}. *Likewise,* σ *and* ρ *are considered as living in* $\mathscr{H}_Z$ *by padding them with zero eigenvalues as necessary.*

We have assumed here that 2^λ is rational. If 2^λ is irrational, we say that σ λ-majorizes ρ if for all rational $2^{\lambda'}$ with $\lambda' < \lambda$, then $\sigma \xrightarrow{\lambda'} \rho$.

The following proposition guarantees that the definition above does not depend on the exact values of λ_1 and λ_2 but only on their difference. This is the same as saying that a fully mixed state cannot act as a catalyst.

Proposition 9. *For any* $\sigma, \rho \in \mathscr{P}(\mathscr{H}_Z)$, *and for any* n, *we have* $\sigma \succ_w \rho$ *if and only if* $\sigma \otimes \frac{\mathbb{1}_n}{n} \succ_w \rho \otimes \frac{\mathbb{1}_n}{n}$.

Proof. If $\sigma \succ_w \rho$, then the majorization passes over the tensor product, and thus proves the claim. Conversely, if $\sigma \otimes \frac{\mathbb{1}_n}{n} \succ_w \rho \otimes \frac{\mathbb{1}_n}{n}$, then in particular, for any $k \leqslant d$,

$$\sum_{i=1}^{n\cdot k} \lambda_i^{\downarrow}(\tfrac{\mathbb{1}_n}{n} \otimes \sigma) \geqslant \sum_{i=1}^{n\cdot k} \lambda_i^{\downarrow}(\tfrac{\mathbb{1}_n}{n} \otimes \rho)\ . \tag{42}$$

(d is the maximum rank of σ or ρ.) But $\lambda_{in}^{\downarrow}(\frac{\mathbb{1}_n}{n} \otimes \sigma) = \frac{1}{n}\lambda_i^{\downarrow}(\sigma)$ and thus

$$\sum_{i=1}^{k} \lambda_i^{\downarrow}(\sigma) \geqslant \sum_{i=1}^{k} \lambda_i^{\downarrow}(\rho)\ .$$

☐

The following proposition is a direct consequence of the definition of lambda-majorization, and just states that you can move around randomness into or out of the ancillas in the definition of lambda-majorization.

Proposition 10. *For any* $\sigma \in \mathscr{P}(\mathscr{H}_X)$, $\rho \in \mathscr{P}(\mathscr{H}_Y)$, *and for any* $\lambda \in \mathbb{R}$, $n > 0$, *we have*

$$\tfrac{1}{n}\mathbb{1}_n \otimes \sigma \xrightarrow{\lambda} \rho \ \Leftrightarrow\ \sigma \xrightarrow{\lambda + \log n} \rho$$

and

$$\sigma \xrightarrow{\lambda-\log n} \rho \;\Leftrightarrow\; \sigma \xrightarrow{\lambda} \tfrac{1}{n}\mathbb{1}_n \otimes \rho \ .$$

Similarly to Thm. 6 and to Prop. 7, it is possible to characterize lambda-majorization by the existence of a matrix relating the vector of eigenvalues that satisfies some specific normalization conditions.

Proposition 11. *Let $\sigma \in \mathscr{P}(\mathscr{H}_X)$ and $\rho \in \mathscr{P}(\mathscr{H}_Y)$. Then $\sigma \xrightarrow{\lambda} \rho$ if and only if there exists a $d_Y \times d_X$ matrix $T_i^{\ k}$ such that $\lambda_i(\rho) = \sum_k T_i^{\ k}\lambda_k(\sigma)$, satisfying $T_i^{\ k} \geqslant 0$, $\sum_i T_i^{\ k} \leqslant 1$, and $\sum_k T_i^{\ k} \leqslant 2^{-\lambda}$.*

Proof of Prop. 11. Suppose $2^{-\lambda_1}\mathbb{1}_A \otimes \sigma \succ_w 2^{-\lambda_2}\mathbb{1}_B \otimes \rho$ with $\lambda = \lambda_1 - \lambda_2$. Then there exists a doubly substochastic matrix $S_{bi}^{\ ak}$ such that

$$\lambda_{bi}\big(2^{-\lambda_2}\mathbb{1}_B \otimes \rho\big) = \sum_{ak} S_{bi}^{\ ak}\, \lambda_{ak}\big(2^{-\lambda_1}\mathbb{1}_A \otimes \sigma\big) \ ,$$

with $S_{bi}^{\ ak} \geqslant 0$, $\sum_{bi} S_{bi}^{\ ak} \leqslant 1$ and $\sum_{ak} S_{bi}^{\ ak} \leqslant 1$. (Indices a and b refer to the mixed ancillas of respective sizes 2^{λ_1} and 2^{λ_2}. Since we are considering weak submajorization, we can safely ignore all zero eigenvalues and consider only the subspaces (of different sizes on the left and right hand side of the majorization) on which σ, ρ, $\mathbb{1}_A$ and $\mathbb{1}_B$ have support, as in Prop. 7.)

Now we have

$$\begin{aligned}\lambda_i(\rho) &= \sum_b \lambda_{bi}\big(2^{-\lambda_2}\mathbb{1}_B \otimes \rho\big)\\ &= \sum_{a\,b\,k} S_{bi}^{\ ak}\, \lambda_{ak}\big(2^{-\lambda_1}\mathbb{1}_A \otimes \sigma\big)\\ &= \sum_k \left(\sum_{ab} 2^{-\lambda_1}\, S_{bi}^{\ ak}\right) \lambda_k(\sigma) \ ,\end{aligned}$$

so one can define

$$T_i^{\ k} = \sum_{ab} 2^{-\lambda_1}\, S_{bi}^{\ ak} \ ,$$

which fulfills $\lambda_i(\rho) = \sum_k T_i^{\ k}\, \lambda_k(\sigma)$. Because S is doubly substochastic, and using the fact that indices a (resp. b) range to 2^{λ_1} (2^{λ_2}), the matrix T satisfies

$$\sum_i T_i^{\ k} = \sum_{i\,a\,b} 2^{-\lambda_1} S_{bi}^{\ ak} = \sum_a 2^{-\lambda_1} \sum_{bi} S_{bi}^{\ ak} \leqslant 1 \ ,$$

as well as

$$\begin{aligned}\sum_k T_i^{\ k} = \sum_{k\,a\,b} 2^{-\lambda_1} S_{bi}^{\ ak} &= \sum_b 2^{-\lambda_1} \sum_{ak} S_{bi}^{\ ak}\\ &\leqslant \sum_b 2^{-\lambda_1} = 2^{-\lambda} \ .\end{aligned}$$

Additionally, $T_i^{\ k} \geqslant 0$ because $S_{bi}^{\ ak} \geqslant 0$.

Conversely, suppose that a matrix $T_i^{\ k}$ exists, with $T_i^{\ k} \geqslant 0$, $\sum_i T_i^{\ k} \leqslant 1$, $\sum_k T_i^{\ k} \leqslant 2^{-\lambda}$, and $\lambda_i(\rho) = \sum_k T_i^{\ k}\lambda_k(\sigma)$. Let λ_1, λ_2 such that $\lambda = \lambda_1 - \lambda_2$ and such that 2^{λ_1}, 2^{λ_2} are integers. Then let $S_{bi}^{\ ak} = 2^{-\lambda_2} T_i^{\ k}$ for all a, b. Then $S_{bi}^{\ ak} \geqslant 0$ and S satisfies

$$\sum_{ak} S_{bi}^{\ ak} = 2^{-\lambda_2} \sum_{ak} T_i^{\ k} \leqslant 2^{-\lambda_2}\Big(\sum_a 1\Big)\, 2^{-\lambda} = 1 \ ,$$

as well as

$$\sum_{bi} S_{bi}^{\ ak} = 2^{-\lambda_2} \sum_{bi} T_i^{\ k} \leqslant 2^{-\lambda_2}\Big(\sum_b 1\Big) = 1 \ .$$

The required weak submajorization for the desired lambda-majorization is provided by this doubly substochastic matrix,

$$\begin{aligned}\lambda_{bi}\big(2^{-\lambda_2}\mathbb{1}_B \otimes \rho\big) &= 2^{-\lambda_2}\lambda_i(\rho) = 2^{-\lambda_2} \sum_k T_i^{\ k}\lambda_k(\sigma)\\ &= 2^{-\lambda_2} \sum_k T_i^{\ k} \sum_a \lambda_{ak}\big(2^{-\lambda_1}\mathbb{1}_A \otimes \sigma\big)\\ &= \sum_{ak} S_{bi}^{\ ak} \lambda_{ak}\big(2^{-\lambda_1}\mathbb{1}_A \otimes \sigma\big) \ .\end{aligned}$$

□

B. Formulation of Lambda-Majorization in Terms of Maps

Majorization can also be characterized in terms of unital, trace-preserving completely positive maps [96–99].

Proposition 12. *Two positive semidefinite matrices σ and ρ satisfy $\sigma \succ \rho$ if and only if there exists a trace-preserving, unital, completely positive map $\mathcal{E}$ satisfying $\mathcal{E}(\sigma) = \rho$.*

Similarly, one can prove an analogous characterization of weak submajorization. The proof of this proposition will be given later.

Proposition 13. *Let $\sigma \in \mathscr{P}(\mathscr{H}_X)$ and $\rho \in \mathscr{P}(\mathscr{H}_Y)$. Then $\sigma \succ_w \rho$ if and only if there exists a completely positive map $\mathcal{E}_{X\to Y} : \mathscr{L}(\mathscr{H}_X) \to \mathscr{L}(\mathscr{H}_Y)$ such that $\mathcal{E}_{X\to Y}(\sigma) = \rho$, with $\mathcal{E}$ satisfying $\mathcal{E}_{X\to Y}(\mathbb{1}_X) \leqslant \mathbb{1}_Y$ and $\mathcal{E}^\dagger_{X\to Y}(\mathbb{1}_Y) \leqslant \mathbb{1}_X$.*

Let's say that $\mathcal{E}_{X\to Y}$ is *subunital* if $\mathcal{E}_{X\to Y}(\mathbb{1}_X) \leqslant \mathbb{1}_Y$. Then the two conditions on the structure of the map $\mathcal{E}_{X\to Y}$ in the above proposition require the map to be subunital and trace-nonincreasing.

A subunital trace-nonincreasing completely positive map can always be seen as part of a unital, trace-preserving completely positive map on a larger Hilbert space. This is analogous of the result that doubly substochastic matrices are submatrices of stochastic matrices [94].

In the following, let $\mathbb{1}_{X\to Z}$ (resp. $\mathbb{1}_{Y\to Z}$) denote the canonical embedding isometry, and define $\mathbb{1}_{Z\to X}$ (resp. $\mathbb{1}_{Z\to Y}$) as the canonical projection partial isometry. Let

also $\mathbb{1}_X$ (resp. $\mathbb{1}_Y$) be the projector onto the subspace $\mathscr{H}_X$ (resp. $\mathscr{H}_Y$) within $\mathscr{H}_Z$.

Proposition 14. *Let $\mathcal{E}_{Z\to Z}$ be a unital, trace-preserving completely positive map. Let $\mathscr{H}_X$ and $\mathscr{H}_Y$ be two subspaces of $\mathscr{H}_Z$. Then the map $\mathcal{E}'_{X\to Y}(\cdot) = \mathbb{1}_{Z\to Y}\mathcal{E}_{Z\to Z}(\mathbb{1}_{X\to Z}(\cdot)\,\mathbb{1}_{Z\to X})\,\mathbb{1}_{Y\to Z}$ is subunital and trace-nonincreasing.*

Conversely, let $\mathscr{H}_X$ and $\mathscr{H}_Y$ be two finite-dimensional Hilbert spaces and let $\mathcal{E}'_{X\to Y}$ be any trace-nonincreasing, subunital completely positive map. Then there exists a finite-dimensional Hilbert space $\mathscr{H}_Z$ with isometries $\mathbb{1}_{X\to Z}$, $\mathbb{1}_{Y\to Z}$ and partial isometries $\mathbb{1}_{Z\to X}$, $\mathbb{1}_{Z\to Y}$, and a completely positive, unital, trace-preserving map $\mathcal{E}_{Z\to Z}$, such that

$$\mathcal{E}'_{X\to Y}(\cdot) = \mathbb{1}_{Z\to Y}\,\mathcal{E}_{Z\to Z}(\mathbb{1}_{X\to Z}(\cdot)\,\mathbb{1}_{Z\to X})\,\mathbb{1}_{Y\to Z}\ . \tag{43}$$

The space $\mathscr{H}_Z$ and map $\mathcal{E}_{Z\to Z}$ may be chosen as $\mathscr{H}_Z = \mathscr{H}_X \oplus \mathscr{H}_Y$ and

$$\begin{aligned}\mathcal{E}_{Z\to Z}(\cdot) =&\\ &\mathbb{1}_{Y\to Z}\,\mathcal{E}'_{X\to Y}(\mathbb{1}_{Z\to X}(\cdot)\,\mathbb{1}_{X\to Z})\,\mathbb{1}_{Z\to Y}\\ &+\ \mathbb{1}_{X\to Z}\,\mathcal{E}'^{\dagger}(\mathbb{1}_{Z\to Y}(\cdot)\,\mathbb{1}_{Y\to Z})\,\mathbb{1}_{Z\to X}\\ &+\ \mathbb{1}_{Y\to Z}\sqrt{G_Y}\,\mathbb{1}_{Z\to Y}(\cdot)\,\mathbb{1}_{Y\to Z}\sqrt{G_Y}\,\mathbb{1}_{Z\to Y}\\ &+\ \mathbb{1}_{X\to Z}\sqrt{H_X}\,\mathbb{1}_{Z\to X}(\cdot)\,\mathbb{1}_{X\to Z}\sqrt{H_X}\,\mathbb{1}_{Z\to X}\ ,\end{aligned} \tag{44}$$

where $G_Y = \mathbb{1}_Y - \mathcal{E}'_{X\to Y}(\mathbb{1}_X)$ and $H_X = \mathbb{1}_X - \mathcal{E}'^{\dagger}(\mathbb{1}_Y)$.

The space $\mathscr{H}_Z$ may also be chosen to be any space bigger than $\mathscr{H}_X \oplus \mathscr{H}_Y$.

In order to generalize this concept to our lambda-majorization, let's introduce the concept of an α-subunital map. These generalize the notion of subunital maps to arbitrary normalizations.

α-subunital Maps. We'll call a map $\mathcal{T}_{X\to Y}$ *α-subunital* if it satisfies $\mathcal{T}_{X\to Y}(\mathbb{1}_X) \leqslant \alpha\mathbb{1}_Y$.

Proposition 15 (Composition of α-subunital maps)**.** *Let $\mathscr{H}_W \in \mathscr{H}_Z$ be another subspace of $\mathscr{H}_Z$ in addition to $\mathscr{H}_X$ and $\mathscr{H}_Y$, and let $\mathcal{T}_{X\to Y}$, $\mathcal{T}'_{Y\to W}$ be completely positive, trace-nonincreasing maps. Assume that $\mathcal{T}_{X\to Y}$ is α-subunital and that $\mathcal{T}'_{Y\to W}$ is β-subunital. Then their composition $[\mathcal{T}'\circ\mathcal{T}]_{X\to W}$ is $\alpha\cdot\beta$-subunital.*

Proof of Prop. 15. The composition of $\mathcal{T}_{X\to Y}$ and $\mathcal{T}'_{Y\to W}$ is trace-nonincreasing,

$$\mathcal{T}^{\dagger}\left(\mathcal{T}'^{\dagger}(\mathbb{1}_W)\right) \leqslant \mathcal{T}^{\dagger}(\mathbb{1}_Y) \leqslant \mathbb{1}_X\ .$$

Their composition is also $\alpha\cdot\beta$-subunital,

$$\mathcal{T}'_{Y\to W}(\mathcal{T}_{X\to Y}(\mathbb{1}_X)) \leqslant \mathcal{T}'_{Y\to W}(\alpha\,\mathbb{1}_Y) \leqslant \alpha\beta\,\mathbb{1}_W\ . \qquad \square$$

Remark 16. *Let $V_{X\to Y}$ be a partial isometry. Then the map $(\cdot)_X \longrightarrow V_{X\to Y}(\cdot)_X V^{\dagger}_{X\leftarrow Y}$ is trace-nonincreasing and subunital.*

In particular, if $\mathcal{T}_{Z\to Z}$ is a $2^{-\lambda}$-subunital, trace-nonincreasing map, then $\mathcal{T}_{X\to Y}$, defined by $\mathcal{T}_{X\to Y}(\cdot) = \mathbb{1}_{Z\to Y}\mathcal{T}_{Z\to Z}(\mathbb{1}_{X\to Z}(\cdot)\,\mathbb{1}_{Z\to X})\,\mathbb{1}_{Y\to Z}$, is also a trace-nonincreasing $2^{-\lambda}$-subunital map.

Proof of Prop. 14. The remark proves the first part of the proposition. To prove the converse, we will show that the expression (44) satisfies the conditions of the claim. Notice first that the channel $\mathcal{E}_{Z\to Z}$ is equal to its own adjoint, i.e. $\mathcal{E}_{Z\to Z} = \mathcal{E}^{\dagger}_{Z\to Z}$. Moreover, the map is unital:

$$\begin{aligned}\mathcal{E}_{Z\to Z}(\mathbb{1}_Z) &= \mathbb{1}_{Y\to Z}(\mathbb{1}_Y - G_Y)\,\mathbb{1}_{Z\to Y}\\ &\quad+\ \mathbb{1}_{X\to Z}(\mathbb{1}_X - H_X)\,\mathbb{1}_{Z\to X}\\ &\quad+\ \mathbb{1}_{Y\to Z}\,G_Y\,\mathbb{1}_{Z\to Y}\\ &\quad+\ \mathbb{1}_{X\to Z}\,H_X\,\mathbb{1}_{Z\to X}\\ &= \mathbb{1}_Y + \mathbb{1}_X = \mathbb{1}_Z\ ,\end{aligned} \tag{45}$$

which makes it automatically trace-preserving, the map being its own adjoint. Condition (43) follows from the definition of $\mathcal{E}_{Z\to Z}$ in (44).

If we choose $\mathscr{H}_Z$ to be any space larger than $\mathscr{H}_X\oplus\mathscr{H}_Y$, we can adapt the definition of $\mathcal{E}_{Z\to Z}$ in (44) and add a term $\mathbb{1}^{\perp}_{X\oplus Y}(\cdot)\,\mathbb{1}^{\perp}_{X\oplus Y}$ (the operator $\mathbb{1}^{\perp}_{X\oplus Y}$ projects onto the orthogonal subspace to $\mathscr{H}_X\oplus\mathscr{H}_Y$ in $\mathscr{H}_Z$). In this case $\mathcal{E}_{Z\to Z}$ would still satisfy all the required properties. $\square$

We now have all the necessary tools to prove Proposition I of the Main Text.

Proposition 17 (Proposition I of the Main Text)**.** *Let $\mathscr{H}_K$ and $\mathscr{H}_L$ be finite dimensional Hilbert spaces, and let $\mathcal{E}'_{K\to L}$ be a completely positive, trace-nonincreasing, subunital map. Then there exists finite dimensional Hilbert spaces $\mathscr{H}_Q$ and $\mathscr{H}_{Q'}$, and a completely positive, trace-preserving, unital map $\mathcal{E}_{KQ\to LQ'}$ such that*

$$\begin{aligned}\mathcal{E}'_{K\to L}(\cdot) = (\mathbb{1}_L\otimes\langle \mathrm{f}|_{Q'})\ \cdot&\\ \mathcal{E}_{KQ\to LQ'}\,[(\mathbb{1}_K\otimes|\mathrm{i}\rangle_Q)\,(\cdot)\,(\mathbb{1}_K\otimes\langle\mathrm{i}|_Q)]&\\ \cdot\ (\mathbb{1}_L\otimes|\mathrm{f}\rangle_{Q'})&\ ,\end{aligned} \tag{46}$$

for some pure states $|\mathrm{i}\rangle_Q$, $|\mathrm{f}\rangle_{Q'}$. In addition, $\dim(\mathscr{H}_K\otimes\mathscr{H}_Q) = \dim(\mathscr{H}_L\otimes\mathscr{H}_{Q'})$.

Proof. Apply the converse in Prop. 14 to dilate the subunital map $\mathcal{E}'_{K\to L}$ to a unital map $\mathcal{E}_{Z\to Z}$ (take $\mathscr{H}_X = \mathscr{H}_K$ and $\mathscr{H}_Y = \mathscr{H}_L$).

We may choose $\mathscr{H}_Z = \mathscr{H}_X\oplus\mathscr{H}_Y\oplus\mathscr{H}_W$, where $\mathscr{H}_W$ is a space whose dimension we haven't yet fixed. We would like the space $\mathscr{H}_Z$ to factorize as both

$$\mathscr{H}_Z = \mathscr{H}_K\otimes\mathscr{H}_Q\ ; \tag{47}$$

$$\mathscr{H}_Z = \mathscr{H}_L\otimes\mathscr{H}_{Q'}\ , \tag{48}$$

for some systems $\mathscr{H}_Q$ and $\mathscr{H}_{Q'}$. A necessary and sufficient condition for that is

$$\dim(\mathscr{H}_Z) = \dim(\mathscr{H}_K)\times\dim(\mathscr{H}_Q)\ ; \tag{49}$$

$$\dim(\mathscr{H}_Z) = \dim(\mathscr{H}_L)\times\dim(\mathscr{H}_{Q'})\ , \tag{50}$$

where $\dim(\mathscr{H}_Z) = \dim(\mathscr{H}_X) + \dim(\mathscr{H}_Y) + \dim(\mathscr{H}_W)$. We thus fix the dimension of $\mathscr{H}_Z$ to be

$$\dim(\mathscr{H}_Z) = m \times \operatorname{lcm}(\dim(\mathscr{H}_K), \dim(\mathscr{H}_L)) \ , \quad (51)$$

where $\operatorname{lcm}(\cdot,\cdot)$ designates the least common multiple of its arguments and m is some integer chosen such that $\dim(\mathscr{H}_Z) \geqslant \dim(\mathscr{H}_K) + \dim(\mathscr{H}_L)$. We have now fixed $\dim(\mathscr{H}_Z)$, and this in turn implicitly fixes the dimension of $\mathscr{H}_W$.

This choice of $\mathscr{H}_Z = \mathscr{H}_K \otimes \mathscr{H}_Q = \mathscr{H}_L \otimes \mathscr{H}_{Q'}$ fixes the embedding isometries $\mathbb{1}_{K\to Z}$, $\mathbb{1}_{L\to Z}$, and projective partial isometries $\mathbb{1}_{Z\to K}$, $\mathbb{1}_{Z\to L}$, as

$$\mathbb{1}_{X\to Z} = \mathbb{1}_{X\to K} \otimes |\mathrm{i}\rangle_Q \ ; \quad \mathbb{1}_{Y\to Z} = \mathbb{1}_{Y\to L} \otimes |\mathrm{f}\rangle_{Q'} \ ;$$
$$\mathbb{1}_{Z\to X} = \mathbb{1}_{K\to X} \otimes \langle\mathrm{i}|_Q \ ; \quad \mathbb{1}_{Z\to Y} = \mathbb{1}_{L\to Y} \otimes \langle\mathrm{f}|_{Q'} \ ,$$

for some fixed states $|\mathrm{i}\rangle_Q$, $|\mathrm{f}\rangle_{Q'}$.

The right hand side of (46) is

$$\begin{aligned}
&(\mathbb{1}_L \otimes \langle\mathrm{f}|_{Q'}) \ \cdot \\
&\qquad \mathcal{E}_{KQ\to LQ'} \left[(\mathbb{1}_K \otimes |\mathrm{i}\rangle_Q)(\cdot)(\mathbb{1}_K \otimes \langle\mathrm{i}|_Q) \right] \\
&\qquad\qquad \cdot \ (\mathbb{1}_L \otimes |\mathrm{f}\rangle_{Q'}) \\
&= \mathbb{1}_{Z\to Y} \ \mathcal{E}_{Z\to Z} \left[\mathbb{1}_{X\to Z} (\cdot) \mathbb{1}_{Z\to X} \right] \ \mathbb{1}_{Y\to Z} \\
&= \mathcal{E}'_{X\to Y}(\cdot) \ .
\end{aligned}$$

We have used the above definition of the embedding and projection (partial) isometries, and applied (43). This proves condition (46).

Note that any choice of $\mathcal{E}_{KQ\to LQ'}$ which would satisfy the conditions of the Proposition would require that $\dim(\mathscr{H}_K \otimes \mathscr{H}_Q) = \dim(\mathscr{H}_L \otimes \mathscr{H}_{Q'})$. Indeed, a unital, trace-preserving completely positive map may only exist if the dimensions of the input and output spaces match: with $\mathcal{E}(\mathbb{1}_{KQ}) = \mathbb{1}_{LQ'}$ and $\mathcal{E}$ trace-preserving we have that $\operatorname{tr}(\mathbb{1}_{KQ}) = \operatorname{tr}\mathbb{1}_{LQ'}$ and thus that the dimensions are equal. □

Proof of Prop. 13. By the weak submajorization condition, if $\operatorname{tr}\rho \neq \operatorname{tr}\sigma$, we must have $\operatorname{tr}\rho < \operatorname{tr}\sigma$. Consider an extension space $\mathscr{H}_{Y'} \in \mathscr{H}_Z$ (consider a larger $\mathscr{H}_Z$ if necessary) in which we extend ρ by many small eigenvalues such that $\operatorname{tr}\rho_{Y\oplus Y'} = \operatorname{tr}\sigma$, while still having $\sigma \succ_w \rho_{Y\oplus Y'}$. Now we have a (regular) majorization, $\sigma \succ \rho_{Y\oplus Y'}$, and can apply Prop. 12.

The obtained map, $\mathcal{E}_{Z\to Z}$, is then unital and trace-preserving. It can be restricted by projecting the input onto $\mathscr{H}_X$ and the output onto $\mathscr{H}_Y$,

$$\mathcal{E}_{X\to Y}(\cdot) = \mathbb{1}_Y \ \mathcal{E}_{Z\to Z}\left(\mathbb{1}_X (\cdot) \mathbb{1}_X\right) \mathbb{1}_Y \ .$$

This restricted operator, by Remark 16, is a valid trace-nonincreasing subunital map (take $\lambda = 0$).

Conversely, if $\mathcal{E}_{X\to Y}$ is a subunital trace-nonincreasing completely positive map with $\mathcal{E}_{X\to Y}(\sigma) = \rho$, then one can dilate it with Proposition 14 to a unital, trace-preserving completely positive map $\mathcal{E}_{Z\to Z}$ such that $\mathbb{1}_Y \mathcal{E}_{Z\to Z}(\sigma \oplus 0_Y) \mathbb{1}_Y = \rho$. Note also that the map $(\cdot) \mapsto \mathbb{1}_Y (\cdot) \mathbb{1}_Y + \mathbb{1}_X (\cdot) \mathbb{1}_X$ is a pinching [94, p. 50, Prob. II.5.5], so we have $\sigma \oplus 0_Y \succ \mathcal{E}_{Z\to Z}(\sigma \oplus 0_Y) \succ \mathbb{1}_X \mathcal{E}_{Z\to Z}(\sigma \oplus 0_Y) \mathbb{1}_X + \mathbb{1}_Y \mathcal{E}_{Z\to Z}(\sigma \oplus 0_Y) \mathbb{1}_Y \succ_w \mathbb{1}_X \mathcal{E}_{Z\to Z}(\sigma \oplus 0_Y) \mathbb{1}_X = \rho$. The last weak submajorization is because some eigenvalues were left out. □

In the same way as lambda majorization can be characterized with differently normalized doubly substochastic maps, it can also be characterized in terms of a differently normalized subunital map.

Proposition 18. *Let $\sigma \in \mathscr{P}(\mathscr{H}_X)$, $\rho \in \mathscr{P}(\mathscr{H}_Y)$ and $\lambda \in \mathbb{R}$. Then $\sigma \xrightarrow{\lambda} \rho$ if and only if there exists a completely positive map $\mathcal{T}_{X\to Y} : \mathscr{L}(\mathscr{H}_X) \to \mathscr{L}(\mathscr{H}_Y)$ such that $\mathcal{T}_{X\to Y}(\sigma) = \rho$, that is $2^{-\lambda}$-subunital and trace-nonincreasing.*

Proof of Prop. 18. **"⇒".** Assume first that $2^{-\lambda_1}\mathbb{1}_A \otimes \sigma \succ_w 2^{-\lambda_2}\mathbb{1}_B \otimes \rho$, with $\mathscr{H}_A$, $\mathscr{H}_B$ (of respective sizes 2^{λ_1} and 2^{λ_2}) being subsystems of an ancilla system $\mathscr{H}_C$, with $\lambda = \lambda_1 - \lambda_2$.

By Prop. 13, there exists a subunital trace-nonincreasing completely positive map $\mathcal{E}_{AX\to BY}$, such that

$$\mathcal{E}_{AX\to BY}(2^{-\lambda_1}\mathbb{1}_A \otimes \sigma) = 2^{-\lambda_2}\mathbb{1}_B \otimes \rho \ . \quad (52)$$

Now let the map $\mathcal{T}$ be defined by

$$\mathcal{T}_{X\to Y}(\cdot) = \operatorname{tr}_B\left[\mathcal{E}_{AX\to BY}\left(2^{-\lambda_1}\mathbb{1}_A \otimes (\cdot)\right)\right] \ . \quad (53)$$

This map is trace-nonincreasing,

$$\begin{aligned}
\mathcal{T}^{\dagger}_{X\leftarrow Y}(\mathbb{1}_Y) &= 2^{-\lambda_1}\operatorname{tr}_A\left[\mathcal{E}^{\dagger}_{AX\leftarrow BY}(\mathbb{1}_{BY})\right] \\
&\leqslant 2^{-\lambda_1}\operatorname{tr}_A(\mathbb{1}_{AX}) = \mathbb{1}_X \ ,
\end{aligned}$$

and $2^{-\lambda}$-subunital,

$$\begin{aligned}
\mathcal{T}_{X\to Y}(\mathbb{1}_X) = 2^{-\lambda_1}\operatorname{tr}_B\left[\mathcal{E}(\mathbb{1}_{AX})\right] &\leqslant 2^{-\lambda_1}\operatorname{tr}_B \mathbb{1}_{BY} \\
&= 2^{-\lambda}\mathbb{1}_Y \ .
\end{aligned}$$

The map $\mathcal{T}$ brings σ to ρ,

$$\begin{aligned}
\mathcal{T}_{X\to Y}(\sigma_X) &= \operatorname{tr}_B\left[\mathcal{E}\left(2^{-\lambda_1}\mathbb{1}_A \otimes \sigma_X\right)\right] \\
&= \operatorname{tr}_B\left(2^{-\lambda_2}\mathbb{1}_B \otimes \rho_Y\right) = \rho_Y \ ,
\end{aligned}$$

so that $\mathcal{T}$ satisfies all the claimed properties.

"⇐". To prove the converse, assume that a trace-nonincreasing, $2^{-\lambda}$-subunital map $\mathcal{T}_{X\to Y}$ exists, such that $\mathcal{T}_{X\to Y}(\sigma) = \rho$.

Choose λ_1, λ_2 such that $\lambda = \lambda_1 - \lambda_2$ and such that 2^{λ_1}, 2^{λ_2}, are integers. (Again, in case 2^{λ} is irrational, approximate 2^{λ} arbitrarily well by rational numbers $2^{\lambda'}$.) Choose $\mathscr{H}_C$ large enough to contain two subspaces $\mathscr{H}_A$ and $\mathscr{H}_B$ of respective dimensions 2^{λ_1} and 2^{λ_2}. Let

$$\mathcal{E}_{AX\to BY}(\cdot) = 2^{-\lambda_2}\mathbb{1}_B \otimes \mathcal{T}_{X\to Y}(\operatorname{tr}_A(\cdot)) \ . \quad (54)$$

This map is trace-nonincreasing,

$$\mathcal{E}^\dagger\left(\mathbb{1}_{BY}\right) = 2^{-\lambda_2}\mathbb{1}_A \otimes \mathcal{T}^\dagger\left(\operatorname{tr}_B \mathbb{1}_{BY}\right) = 2^{-\lambda_2}\mathbb{1}_A \otimes \mathcal{T}^\dagger\left(2^{\lambda_2}\mathbb{1}_Y\right) \leqslant \mathbb{1}_{AX}\ ,$$

and subunital,

$$\mathcal{E}\left(\mathbb{1}_{AX}\right) = 2^{-\lambda_2}\mathbb{1}_B \otimes \mathcal{T}\left(\operatorname{tr}_A \mathbb{1}_{AX}\right) = 2^{-\lambda_2}\mathbb{1}_B \otimes \mathcal{T}\left(2^{\lambda_1}\mathbb{1}_X\right) \leqslant \mathbb{1}_{BY}\ ,$$

since $\lambda = \lambda_1 - \lambda_2$ and $\mathcal{T}$ is $2^{-\lambda}$-subunital. Also,

$$\mathcal{E}\left(2^{-\lambda_1}\mathbb{1}_A \otimes \sigma_X\right) = 2^{-\lambda_2}\mathbb{1}_B \otimes \mathcal{T}\left(\operatorname{tr}_A\left(2^{-\lambda_1}\mathbb{1}_A \otimes \sigma_X\right)\right) = 2^{-\lambda_2}\mathbb{1}_B \otimes \mathcal{T}\left(\sigma_X\right) = 2^{-\lambda_2}\mathbb{1}_B \otimes \rho_Y\ .$$

By Prop. 13, we eventually have

$$2^{-\lambda_1}\mathbb{1}_A \otimes \sigma_X \succ_w 2^{-\lambda_2}\mathbb{1}_B \otimes \rho_Y\ . \qquad \square$$

Remark 19. *A trace-nonincreasing, $2^{-\lambda}$-subunital completely positive map $\mathcal{T}_{X\to Y}$ can always be written as in Eq. (53) for a sub-unital trace-nonincreasing completely positive map $\mathcal{E}_{AX\to BY}$, which itself can always be written as projections of a unital map $\mathcal{E}_{CZ\to CZ}$ (see text of the previous proof, and Prop. 14).*

Conversely, for any unital map $\mathcal{E}_{CZ\to CZ}$ with $\mathcal{E}\left(2^{-\lambda_1}\mathbb{1}\otimes\sigma_X\right) = 2^{-\lambda_2}\mathbb{1}\otimes\rho_Y$, in particular for any noisy operation in our framework, the map $\mathcal{T}$ obtained by Eq. (53) is trace-nonincreasing and $2^{-\lambda}$-subunital.

In particular, for our purposes of optimizing λ over all possible processes of our framework with an additional condition to the map carrying out the process (namely to preserve correlations between our system X and the reference system R), we may impose that condition directly on the map $\mathcal{T}$ to obtain an upper bound on λ.

C. Properties for quantum states

We will consider in this section some useful properties of lambda-majorization in the case where we consider normalized states σ, ρ. Here, weak majorization automatically implies (regular) majorization because $\operatorname{tr}\sigma = \operatorname{tr}\rho = 1$.

In this section, let $\sigma \in \mathscr{S}_=(\mathscr{H}_X)$ and $\rho \in \mathscr{S}_=(\mathscr{H}_Y)$.

Proposition 20 (Lambda-Majorizing a Pure State)**.** *For any pure state $|0\rangle \in \mathscr{H}_Z$, we have $\sigma \xrightarrow{\lambda} |0\rangle\langle 0|$ if and only if* $\operatorname{rank}\sigma \leqslant 2^{-\lambda}$ *(obviously λ has to be negative or zero). Equivalently, $\sigma \succ \frac{1}{n}\mathbb{1}_n$ if and only if* $\operatorname{rank}\sigma \leqslant n$.

Proof of Prop. 20. Assume first that $\sigma \xrightarrow{\lambda} |0\rangle\langle 0|$. Here $\mathscr{H}_Y$ is the one-dimensional space spanned by $|0\rangle$, and take $\mathscr{H}_X$ the subspace on which σ has its support. By Prop. 11 there exists a single-row matrix ${T_i}^k$ satisfying ${T_i}^k \geqslant 0$, $\sum_i {T_i}^k = T_{i=1}^k \leqslant 1\ \forall k$, $\sum_k {T_i}^k \leqslant 2^{-\lambda}$ such that $1 = \lambda_{i=1}(|0\rangle\langle 0|) = \sum_k T_{i=1}^k \lambda_k(\sigma)$. We also have $\lambda_k(\sigma) \neq 0$ because σ has nonzero eigenvalues in $\mathscr{H}_X$. Then $\sum_k T_{i=1}^k \lambda_k(\sigma) = 1 = \sum_k \lambda_k(\sigma)$ implies $T_{i=1}^k = 1\ \forall k$. That is, the condition $\sum_k T_{i=1}^k \leqslant 2^{-\lambda}$ forces $T_{i=1}^k$ to have at most $2^{-\lambda}$ elements, i.e. the rank of σ may not exceed $2^{-\lambda}$.

The converse holds because any state majorizes a uniform state of the same rank. $\square$

Proposition 21 (Condition on Support Sizes for Lambda-Majorization)**.** *If $\sigma \xrightarrow{\lambda} \rho$, then* $\operatorname{rank}\sigma \leqslant 2^{-\lambda}\operatorname{rank}\rho$.

Proof of Prop. 21. Notice that $\rho \succ \frac{1}{\operatorname{rank}\rho}\mathbb{1}_{\operatorname{rank}\rho}$, and thus $\sigma \xrightarrow{\lambda} \frac{1}{\operatorname{rank}\rho}\mathbb{1}_{\operatorname{rank}\rho}$. Then, by Prop. 10 we have

$$\sigma \xrightarrow{\lambda - \log\operatorname{rank}\rho} |0\rangle\langle 0|\ ;$$

it remains to apply Prop. 20. $\square$

Proposition 22 (Being Lambda-Majorized by a Pure State)**.** *Let the state ρ have maximum eigenvalue $\lambda_{\max}(\rho)$. For any pure state $|0\rangle$, we have $|0\rangle\langle 0| \xrightarrow{\lambda} \rho$ if and only if $\lambda_{\max}(\rho) \leqslant 2^{-\lambda}$. Equivalently, $\frac{1}{n}\mathbb{1}_n \succ \rho$ if and only if $\lambda_{\max}(\rho) \leqslant \frac{1}{n}$.*

Proof of Prop. 22. Let ${T_i}^k$ be as in Prop. 11. Note here k only takes value 1, because we consider $\mathscr{H}_Y$ being the one-dimensional space spanned by $|0\rangle$. Then $\lambda_i(\rho) = \sum_k {T_i}^k \lambda_k(|0\rangle\langle 0|) = {T_i}^{k=1}$ and thus ${T_i}^k = \lambda_i(\rho)$. Then $2^{-\lambda} \geqslant \sum_k {T_i}^k = T_i^{k=1} = \lambda_i(\rho)$ for all i. In particular, $2^{-\lambda} \geqslant \lambda_{\max}(\rho)$.

Conversely, if $\lambda_{\max}(\rho) \leqslant 2^{-\lambda}$, then let ${T_i}^{k=1} = \lambda_i(\rho)$. This matrix T satisfies the conditions in Prop. 11 and thus $|0\rangle\langle 0| \xrightarrow{\lambda} \rho$. $\square$

D. Optimal Lambda Majorization for Normalized States and Relation to Single-Shot Entropy Measures

Define the *absorbed randomness* (or *relative mixedness* [59]) of a transition from σ to ρ as the maximal amount of randomness that you can get rid of, or the minimal amount of randomness that you have to generate, in a noisy operation process:

$$R(\sigma \to \rho) = \sup\left\{\lambda : \sigma \xrightarrow{\lambda} \rho\right\}\ . \tag{55}$$

Recent work has shown that this measure is relevant for the amount of extractable work of processes acting on arrays of Szilard boxes [59].

The absorbed randomness has some tight relations to single-shot entropy measures, which we present here. These are reformulations of results shown in [55, 58].

Proposition 23. *The absorbed randomness defined above satisfies the following bounds.*

$$H_{\min}(\rho) - H_0(\sigma) \leqslant R(\sigma \to \rho) \leqslant H_0(\rho) - H_0(\sigma)\ .$$

Proposition 24. *If $|0\rangle$ denotes any pure state, then the following relations hold:*

$$R(|0\rangle \to \rho) = H_{\min}(\rho) \ , \tag{56}$$
$$R(\sigma \to |0\rangle) = -H_0(\sigma) \ . \tag{57}$$

Similar explicit values can be obtained in the case where either the initial state or the target state is mixed.

Proposition 25. *If $\frac{\mathbb{1}_n}{n}$ denotes the fully mixed state on $\log n$ qubits, then:*

$$R(\tfrac{\mathbb{1}_n}{n} \to \rho) = H_{\min}(\rho) - \log n \ , \tag{58}$$
$$R(\sigma \to \tfrac{\mathbb{1}_n}{n}) = \log n - H_0(\sigma) \ . \tag{59}$$

Proof of Prop. 23. Lower bound: Let $\lambda_1 = H_{\min}(\rho) = -\log \lambda_{\max}(\rho)$ and $\lambda_2 = H_0(\sigma) = \log \operatorname{rank} \sigma$. By Proposition 22, we have $2^{-\lambda_1}\mathbb{1}_{2^{\lambda_1}} \succ \rho$ and by Proposition 20, $\sigma \succ 2^{-\lambda_2}\mathbb{1}_{2^{\lambda_2}}$. The majorization carries over to the tensor product, $2^{-\lambda_1}\mathbb{1}_{2^{\lambda_1}} \otimes \sigma \succ 2^{-\lambda_2}\mathbb{1}_{2^{\lambda_2}} \otimes \rho$, and $\lambda_1 - \lambda_2$ is a valid maximization candidate for (55).

Upper bound: Let $\lambda = R(\sigma \to \rho)$ satisfying $\sigma \xrightarrow{\lambda} \rho$. Proposition 21 immediately yields $2^{\lambda} \leqslant \frac{\operatorname{rank}\rho}{\operatorname{rank}\sigma}$, and

$$R(\sigma \to \rho) = \lambda \leqslant \log \operatorname{rank} \rho - \log \operatorname{rank} \sigma \ .$$

Recalling the definition of the Rényi-0 entropy $H_0(\sigma) = \log \operatorname{rank} \sigma$ yields the required upper bound. □

Proof of Prop. 24. Equation (57) follows from the bounds of Proposition 23, which become tight in this special case. Equality (56) is a direct consequence of Prop. 22. □

Proof of Prop. 25. The bounds of Proposition 23 become tight for (59). Equality (58) is again a consequence of Prop. 22, recalling Prop. 10 which allows us to write $|0\rangle\langle 0| \xrightarrow{\lambda + \log n} \rho$ instead of $\frac{\mathbb{1}_n}{n} \xrightarrow{\lambda} \rho$. □

SUPPLEMENTARY REFERENCES

[1] Leff, H. S. & Rex, A. F. *Maxwell's Demon 2: Entropy, Classical and Quantum Information, Computing* (Taylor & Francis, 2010).

[2] Szilard, L. Über die Entropieverminderung in einem thermodynamischen System bei Eingriffen intelligenter Wesen. *Zeitschrift für Physik* **53**, 840–856 (1929).

[3] Brillouin, L. Maxwell's demon cannot operate: Information and entropy. I. *Journal of Applied Physics* **22**, 334 (1951).

[4] Gabor, D. IV light and information. In Wolf, E. (ed.) *Progress in Optics*, vol. 1 of *Progress in Optics*, 109–153 (Elsevier, 1961).

[5] Landauer, R. Irreversibility and heat generation in the computing process. *IBM Journal of Research and Development* **5**, 183–191 (1961).

[6] Keyes, R. W. & Landauer, R. Minimal energy dissipation in logic. *IBM Journal of Research and Development* **14**, 152–157 (1970).

[7] Bennett, C. H. The thermodynamics of computation—a review. *International Journal of Theoretical Physics* **21**, 905–940 (1982).

[8] Bennett, C. H. Logical reversibility of computation. *IBM Journal of Research and Development* **17**, 525–532 (1973).

[9] Bennett, C. H. Notes on Landauer's principle, reversible computation and Maxwell's demon. *Studies in History and Philosophy of Modern Physics* **34**, 501–510 (2003).

[10] Bennett, C. H. Notes on the history of reversible computation. *IBM Journal of Research and Development* **32**, 16–23 (1988).

[11] Maruyama, K., Nori, F. & Vedral, V. Colloquium: The physics of Maxwell's demon and information. *Reviews of Modern Physics* **81**, 1–23 (2009).

[12] Earman, J. & Norton, J. D. Exorcist XIV: The wrath of Maxwell's demon. Part I. from Maxwell to Szilard. *Studies In History and Philosophy of Science Part B: Studies In History and Philosophy of Modern Physics* **29**, 435–471 (1998).

[13] Earman, J. & Norton, J. D. Exorcist XIV: The wrath of Maxwell's demon. Part II. from Szilard to Landauer and beyond. *Studies In History and Philosophy of Science Part B: Studies In History and Philosophy of Modern Physics* **30**, 1–40 (1999).

[14] Piechocinska, B. Information erasure. *Physical Review A* **61**, 062314 (2000).

[15] Shizume, K. Heat generation required by information erasure. *Physical Review E* **52**, 3495–3499 (1995).

[16] Jacobs, K. Deriving Landauer's erasure principle from statistical mechanics. Preprint at `http://arxiv.org/abs/quant-ph/0512105` (2005).

[17] Landauer, R. & Woo, J. W. F. Minimal energy dissipation and maximal error for the computational process. *Journal of Applied Physics* **42**, 2301 (1971).

[18] Likharev, K. K. Classical and quantum limitations on energy consumption in computation. *International Journal of Theoretical Physics* **21**, 311–326 (1982).

[19] Landauer, R. Fundamental physical limitations of the computational process. *Annals of the New York Academy of Sciences* **426**, 161–170 (1984).

[20] Landauer, R. Dissipation and noise immunity in computation and communication. *Nature* **335**, 779–784 (1988).

[21] Zurek, W. H. Thermodynamic cost of computation, algorithmic complexity and the information metric. *Nature* **341**, 119–124 (1989).

[22] Landauer, R. The physical nature of information. *Physics Letters A* **217**, 188–193 (1996).

[23] Lloyd, S. Ultimate physical limits to computation. *Nature* **406**, 1047–54 (2000).

[24] Ladyman, J., Presnell, S., Short, A. J. & Groisman, B. The connection between logical and thermodynamic irreversibility. *Studies in History and Philosophy of Science Part B: Studies in History and Philosophy of Modern Physics* **38**, 58–79 (2007).

[25] Jaynes, E. T. Information theory and statistical mechanics. *Physical Review* **106**, 620–630 (1957).

[26] Jaynes, E. T. Information theory and statistical mechanics. II. *Physical Review* **108**, 171–190 (1957).

[27] Jaynes, E. T. Gibbs vs Boltzmann entropies. *American Journal of Physics* **33**, 391 (1965).

[28] Jaynes, E. T. The Gibbs paradox. In Smith, C. R., Erickson, G. & Neudorfer, P. O. (eds.) *Maximum Entropy and Bayesian Methods*, vol. 50 of *Fundamental Theories of Physics*, 1–21 (Springer Netherlands, 1992).

[29] Plenio, M. B. & Vitelli, V. The physics of forgetting: Landauer's erasure principle and information theory. *Contemporary Physics* **42**, 25–60 (2001).

[30] Alicki, R., Horodecki, M., Horodecki, P. & Horodecki, R. Thermodynamics of quantum information systems — hamiltonian description. *Open Systems & Information Dynamics* **11**, 205–217 (2004).

[31] Oppenheim, J., Horodecki, M., Horodecki, P. & Horodecki, R. Thermodynamical approach to quantifying quantum correlations. *Physical Review Letters* **89**, 180402 (2002).

[32] Janzing, D., Wocjan, P., Zeier, R., Geiss, R. & Beth, T. Thermodynamic cost of reliability and low temperatures: Tightening Landauer's principle and the second law. *International Journal of Theoretical Physics* **39**, 2717–2753 (2000).

[33] Janzing, D. *Computer science approach to quantum control*. Habilitation, Universität Karlsruhe, Karlsruhe (2006).

[34] Anders, J., Shabbir, S., Hilt, S. & Lutz, E. Landauer's principle in the quantum domain. *Electronic Proceedings in Theoretical Computer Science* **26**, 13–18 (2010).

[35] Scovil, H. E. D. & Schulz-DuBois, E. O. Three-level masers as heat engines. *Phys. Rev. Lett.* **2**, 262–263 (1959).

[36] Geusic, J. E., Schulz-DuBios, E. O. & Scovil, H. E. D. Quantum equivalent of the carnot cycle. *Phys. Rev.* **156**, 343–351 (1967).

[37] Alicki, R. On the entropy production for the Davies model of heat conduction. *Journal of Statistical Physics* **20**, 671–677 (1979).

[38] Howard, J. Molecular motors: structural adaptations to cellular functions. *Nature* **389**, 561–7 (1997).

[39] Geva, E. & Kosloff, R. On the classical limit of quantum thermodynamics in finite time. *The Journal of Chemical Physics* **97**, 4398 (1992).

[40] Hänggi, P. & Marchesoni, F. Artificial brownian motors: Controlling transport on the nanoscale. *Rev. Mod. Phys.* **81**, 387–442 (2009).

[41] Allahverdyan, A. E. & Nieuwenhuizen, T. M. Extraction of work from a single thermal bath in the quantum regime. *Phys. Rev. Lett.* **85**, 1799–1802 (2000).

[42] Feldmann, T. & Kosloff, R. Quantum lubrication: Suppression of friction in a first-principles four-stroke heat engine. *Phys. Rev. E* **73**, 025107 (2006).

[43] Baugh, J., Moussa, O., Ryan, C. A., Nayak, A. & Laflamme, R. Experimental implementation of heat-bath algorithmic cooling using solid-state nuclear magnetic resonance. *Nature* **438**, 470–3 (2005).

[44] Gemmer, J., Michel, M., Michel, M. & Mahler, G. *Quantum thermodynamics: Emergence of thermodynamic behavior within composite quantum systems* (Springer Verlag, 2009).

[45] Popescu, S., Short, A. J. & Winter, A. Entanglement and the foundations of statistical mechanics. *Nature Physics* **2**, 754–758 (2006).

[46] Linden, N., Popescu, S. & Skrzypczyk, P. How small can thermal machines be? the smallest possible refrigerator. *Physical Review Letters* **105**, 130401 (2010).

[47] Linden, N., Popescu, S., Short, A. J. & Winter, A. On the speed of fluctuations around thermodynamic equilibrium. *New Journal of Physics* **12**, 55021 (2010).

[48] Gogolin, C., Müller, M. P. & Eisert, J. Absence of thermalization in nonintegrable systems. *Physical Review Letters* **106**, 040401 (2011).

[49] Trotzky, S. *et al.* Probing the relaxation towards equilibrium in an isolated strongly correlated one-dimensional bose gas. *Nature Physics* **8**, 325–330 (2012).

[50] Hutter, A. & Wehner, S. Dependence of a quantum-mechanical system on its own initial state and the initial state of the environment it interacts with. *Physical Review A* **87**, 012121 (2013).

[51] Renner, R. *Security of Quantum Key Distribution*. Ph.D. thesis, ETH Zürich (2005). Security of Quantum Key Distribution. Preprint at `http://arxiv.org/abs/quant-ph/0512258` (2005).

[52] Tomamichel, M., Colbeck, R. & Renner, R. A fully quantum asymptotic equipartition property. *IEEE Transactions on Information Theory* **55**, 5840–5847 (2009).

[53] Tomamichel, M. *A Framework for Non-Asymptotic Quantum Information Theory*. Ph.D. thesis, ETH Zurich (2012). A Framework for Non-Asymptotic Quantum Information Theory. Preprint at `http://arxiv.org/abs/1203.2142` (2012).

[54] del Rio, L., Åberg, J., Renner, R., Dahlsten, O. & Vedral, V. The thermodynamic meaning of negative entropy. *Nature* **474**, 61–63 (2011).

[55] Dahlsten, O. C. O., Renner, R., Rieper, E. & Vedral, V. Inadequacy of von Neumann entropy for characterizing extractable work. *New Journal of Physics* **13**, 53015 (2011).

[56] Åberg, J. Truly work-like work extraction via a single-shot analysis. *Nature Communications* **4**, 1925 (2013).

[57] Horodecki, M. & Oppenheim, J. Fundamental limitations for quantum and nanoscale thermodynamics. *Nature Communications* **4**, 2059 (2013).

[58] Egloff, D. *Work Value of Mixed States*. Master's thesis, ETH Zürich (2010).

[59] Egloff, D., Dahlsten, O. C. O., Renner, R. & Vedral, V. Laws of thermodynamics beyond the von Neumann regime. Preprint at `http://arxiv.org/abs/1207.0434` (2012).

[60] Sagawa, T. & Ueda, M. Minimal energy cost for thermodynamic information processing: Measurement and in-

formation erasure. *Physical Review Letters* **102**, 250602 (2009).

[61] Skrzypczyk, P., Short, A. J. & Popescu, S. Extracting work from quantum systems. Preprint at http://arxiv.org/abs/1302.2811 (2013).

[62] Åberg, J. Catalytic coherence. *Physical Review Letters* **113**, 150402 (2014).

[63] Reeb, D. & Wolf, M. M. An improved Landauer principle with finite-size corrections. *New Journal of Physics* **16**, 103011 (2014).

[64] Horodecki, M., Horodecki, P. & Oppenheim, J. Reversible transformations from pure to mixed states and the unique measure of information. *Physical Review A* **67**, 062104 (2003).

[65] Brandão, F. G. S. L., Horodecki, M., Oppenheim, J., Renes, J. M. & Spekkens, R. W. Resource theory of quantum states out of thermal equilibrium. *Physical Review Letters* **111**, 250404 (2013).

[66] Brandão, F., Horodecki, M., Ng, N., Oppenheim, J. & Wehner, S. The second laws of quantum thermodynamics. *Proceedings of the National Academy of Sciences* **112**, 201411728 (2015).

[67] Sagawa, T. & Ueda, M. Information thermodynamics: Maxwell's demon in nonequilibrium dynamics. In Klages, R., Just, W., Jarzynski, C. & Schuster, H. G. (eds.) *Nonequilibrium Statistical Physics of Small Systems: Fluctuation Relations and Beyond*, 181–211 (Wiley-VCH, Weinheim, 2013).

[68] Sagawa, T. & Ueda, M. Fluctuation theorem with information exchange: Role of correlations in stochastic thermodynamics. *Physical Review Letters* **109**, 180602 (2012).

[69] Jacobs, K. Quantum measurement and the first law of thermodynamics: The energy cost of measurement is the work value of the acquired information. *Physical Review E* **86**, 040106 (2012).

[70] Lieb, E. H. & Yngvason, J. The entropy concept for non-equilibrium states. *Proceedings of the Royal Society. Mathematical, physical, and engineering sciences* **469**, 20130408 (2013).

[71] Shannon, C. E. A mathematical theory of communication. *The Bell System Technical Journal* **27**, 379–423 (1948).

[72] Han, T. S. & Verdu, S. Approximation theory of output statistics. In *Proceedings of the IEEE International Symposium on Information Theory*, 153–153 (IEEE, 1993).

[73] Han, T. S. *Information-Spectrum Methods in Information Theory*. Applications of Mathematics Series (Springer, 2002).

[74] Datta, N. & Renner, R. Smooth entropies and the quantum information spectrum. *IEEE Transactions on Information Theory* **55**, 2807–2815 (2009).

[75] Tomamichel, M., Colbeck, R. & Renner, R. Duality between smooth min- and max-entropies. *IEEE Transactions on Information Theory* **56**, 4674–4681 (2010).

[76] Tomamichel, M., Schaffner, C., Smith, A. & Renner, R. Leftover hashing against quantum side information. *IEEE Transactions on Information Theory* **57**, 5524–5535 (2011).

[77] Nielsen, M. A. & Chuang, I. L. *Quantum Computation and Quantum Information* (Cambridge University Press, 2000).

[78] König, R., Renner, R. & Schaffner, C. The operational meaning of min- and max-entropy. *IEEE Transactions on Information Theory* **55**, 4337–4347 (2009).

[79] Rényi, A. On measures of entropy and information. In *Proceedings of the Fourth Berkeley Symposium on Mathematical Statistics and Probability, Vol. 1: Contributions to the Theory of Statistics*, 547–561 (1960).

[80] Haagerup, U. & Musat, M. Factorization and dilation problems for completely positive maps on von Neumann algebras. *Communications in Mathematical Physics* **303**, 555–594 (2011).

[81] Landau, L. J. & Streater, R. F. On birkhoff's theorem for doubly stochastic completely positive maps of matrix algebras. *Linear Algebra and its Applications* **193**, 107–127 (1993).

[82] Bennett, C. H., Bernstein, H. J., Popescu, S. & Schumacher, B. Concentrating partial entanglement by local operations. *Phys. Rev. A* **53**, 2046–2052 (1996).

[83] Bennett, C. H. *et al.* Purification of noisy entanglement and faithful teleportation via noisy channels. *Physical Review Letters* **76**, 722–725 (1996).

[84] Bennett, C. H., DiVincenzo, D. P., Smolin, J. A. & Wootters, W. K. Mixed-state entanglement and quantum error correction. *Physical Review A* **54**, 3824–3851 (1996).

[85] Vidal, G. & Cirac, J. Irreversibility in asymptotic manipulations of entanglement. *Physical Review Letters* **86**, 5803–5806 (2001).

[86] Vitanov, A., Dupuis, F., Tomamichel, M. & Renner, R. Chain rules for smooth min- and max-entropies. *IEEE Transactions on Information Theory* **59**, 2603–2612 (2013).

[87] Hayden, P., Leung, D. W. & Winter, A. Aspects of generic entanglement. *Communications in Mathematical Physics* **265**, 95–117 (2006).

[88] Winter, A. "extrinsic" and "intrinsic" data in quantum measurements: Asymptotic convex decomposition of positive operator valued measures. *Communications in Mathematical Physics* **244**, 157–185 (2004).

[89] Wilde, M. M., Hayden, P., Buscemi, F. & Hsieh, M.-H. The information-theoretic costs of simulating quantum measurements. *Journal of Physics A: Mathematical and Theoretical* **45**, 453001 (2012).

[90] Buscemi, F., Hayashi, M. & Horodecki, M. Global information balance in quantum measurements. *Physical Review Letters* **100**, 210504 (2008).

[91] Berta, M., Renes, J. M. & Wilde, M. M. Identifying the information gain of a quantum measurement. *IEEE Transactions on Information Theory* **60**, 7987–8006 (2014).

[92] Schulman, L. J. & Vazirani, U. V. Molecular scale heat engines and scalable quantum computation. In *Proceedings of the thirty-first annual ACM symposium on Theory of computing - STOC '99*, STOC '99, 322–329. ACM (ACM Press, New York, New York, USA, 1999).

[93] Hardy, G. H., Littlewood, J. E. & Pólya, G. *Inequalities*. Cambridge Mathematical Library (Cambridge University Press, 1952).

[94] Bhatia, R. *Matrix Analysis*. Graduate Texts in Mathematics (Springer, 1997).

[95] Horn, R. A. & Johnson, C. R. *Matrix Analysis* (Cambridge University Press, New York, 1985).

[96] Uhlmann, A. On the Shannon entropy and related functionals on convex sets. *Reports on Mathematical Physics* **1**, 147–159 (1970).

[97] Uhlmann, A. Sätze über Dichtematrizen. *Wiss. Z. Karl-Marx-Univ. Leipzig, Math.-Naturwiss.* **20**, 633–637 (1971).

[98] Uhlmann, A. Endlich-dimensionale Dichtematrizen I. *Wiss. Z. Karl-Marx-Univ. Leipzig, Math.-Naturwiss.* **21**, 421–452 (1972).

[99] Uhlmann, A. Endlich-dimensionale Dichtematrizen II. *Wiss. Z. Karl-Marx-Univ. Leipzig, Math.-Naturwiss.* **22**, 139–177 (1973).

[100] Joe, H. Majorization and divergence. *Journal of Mathematical Analysis and Applications* **148**, 287–305 (1990).

[101] Ruch, E., Schranner, R. & Seligman, T. H. Generalization of a theorem by Hardy, Littlewood, and Pólya. *Journal of Mathematical Analysis and Applications* **76**, 222–229 (1980).

[102] Ruch, E., Schranner, R. & Seligman, T. H. The mixing distance. *The Journal of Chemical Physics* **69**, 386 (1978).

[103] Mead, C. A. Mixing character and its application to irreversible processes in macroscopic systems. *The Journal of Chemical Physics* **66**, 459 (1977).

[104] Barvinok, A. *A Course in Convexity*, vol. 54 of *Graduate Studies in Mathematics* (American Mathematical Society, 2002).

[105] Watrous, J. *Theory of Quantum Information—Lecture notes from Fall 2008* (Online Lecture Notes, 2008). `http://www.cs.uwaterloo.ca/~watrous/quant-info/`.

[106] Marshall, A. W., Olkin, I. & Arnold, B. C. *Inequalities: Theory of Majorization and Its Applications* (Springer, 2010).
